# How to Inactivate Human Ubiquitin E3 Ligases by Mutation

**DOI:** 10.3389/fcell.2020.00039

**Published:** 2020-02-04

**Authors:** Cristina Garcia-Barcena, Nerea Osinalde, Juanma Ramirez, Ugo Mayor

**Affiliations:** ^1^Department of Biochemistry and Molecular Biology, Faculty of Science and Technology, University of the Basque Country (UPV/EHU), Leioa, Spain; ^2^Department of Biochemistry and Molecular Biology, Faculty of Pharmacy, University of the Basque Country (UPV/EHU), Vitoria-Gasteiz, Spain; ^3^Ikerbasque – Basque Foundation for Science, Bilbao, Spain

**Keywords:** ubiquitin, E3, mutation, ligase, inactivation

## Abstract

E3 ubiquitin ligases are the ultimate enzymes involved in the transfer of ubiquitin to substrate proteins, a process that determines the fate of the modified protein. Numerous diseases are caused by defects in the ubiquitin-proteasome machinery, including when the activity of a given E3 ligase is hampered. Thus, inactivation of E3 ligases and the resulting effects at molecular or cellular level have been the focus of many studies during the last few years. For this purpose, site-specific mutation of key residues involved in either protein interaction, substrate recognition or ubiquitin transfer have been reported to successfully inactivate E3 ligases. Nevertheless, it is not always trivial to predict which mutation(s) will block the catalytic activity of a ligase. Here we review over 250 site-specific inactivating mutations that have been carried out in 120 human E3 ubiquitin ligases. We foresee that the information gathered here will be helpful for the design of future experimental strategies.

## Ubiquitination, the Ubiquitin Code and E3 Ligases

Ubiquitin is a 76-amino-acid protein, highly conserved among organisms (Zuin et al., 2014), used–through the ubiquitin-proteasome system- to regulate many cellular processes. Proteins are covalently modified on their Lys residues with ubiquitin via amide isopeptide linkages (Laney and Hochstrasser, 1999). Frequently, ubiquitinated proteins are targeted for degradation through the proteasomal system on an ATP hydrolysis-dependent manner (Hershko and Ciechanover, 1998; Komander and Rape, 2012). But protein ubiquitination participates in a plethora of additional cellular responses including regulation of gene expression, cell signalling, cell cycle, DNA repair and apoptosis (Pickart, 2001; Gilberto and Peter, 2017).

The ubiquitination reaction requires the coordinated action of three types of enzymes termed E1, E2, and E3. First, ubiquitin is activated with ATP in a process carried out by an activating E1 enzyme. Once ubiquitin is activated, it is transferred to the Cys on the active site of a conjugating E2 enzyme. Finally, ubiquitin is generally linked to a Lys of the target protein through an isopeptide bond, formed between the C-terminal carboxyl group of ubiquitin and the ε-amino group of the Lys. Substrate specificity in ubiquitination is attributed to E3 ligases, who are able to interact with both the ubiquitin-charged E2 and the substrates to be modified (Metzger et al., 2014). Like most post-translational modifications (PTMs), ubiquitination is reversible and deubiquitinating enzymes (DUBs) are responsible for hydrolysing the isopeptide bond between ubiquitin and substrate proteins or between ubiquitin molecules.

Proteins can be modified by ubiquitin in a wide range of manners. For instance, in addition to Lys, ubiquitin can be conjugated via a peptide bond to the N-terminal amino group of the substrates (Ciechanover and Ben-Saadon, 2004), as well as to Cys or Ser/Thr residues by thio- or oxy-ester bonds, respectively (Wang et al., 2012). Substrates can be mono-ubiquitinated, meaning modified in a single residue by only one ubiquitin. Multi-mono-ubiquitination occurs when several residues of a given protein are simultaneously modified with one ubiquitin each. Poly-ubiquitination occurs when the C-terminus of another ubiquitin associates to one of the seven Lys (Lys6, Lys11, Lys27, Lys29, Lys33, Lys48, and Lys63) or the N-terminal Met (Met1) on the previously added ubiquitin molecules. Consequently, a ubiquitin chain is formed on the target protein. Depending on how ubiquitin residues are bound together, different ubiquitin chain architectures can be formed: (i) homogenous, if the Lys used throughout the chain is the same (e.g., Lys48-linked chains), (ii) heterogeneous, if they alternate (e.g., Lys48-Lys11-linked chains) and (iii) branched, if multiple Lys of the same ubiquitin are modified at the same time. Altogether, ubiquitin can generate a huge amount of different types of modifications on any given protein (Komander and Rape, 2012). Consequently, ubiquitin-mediated cellular responses will depend not only on the specific residues of the substrate that are modified but also on the topology of the ubiquitin chains that are formed.

**FIGURE 1:**
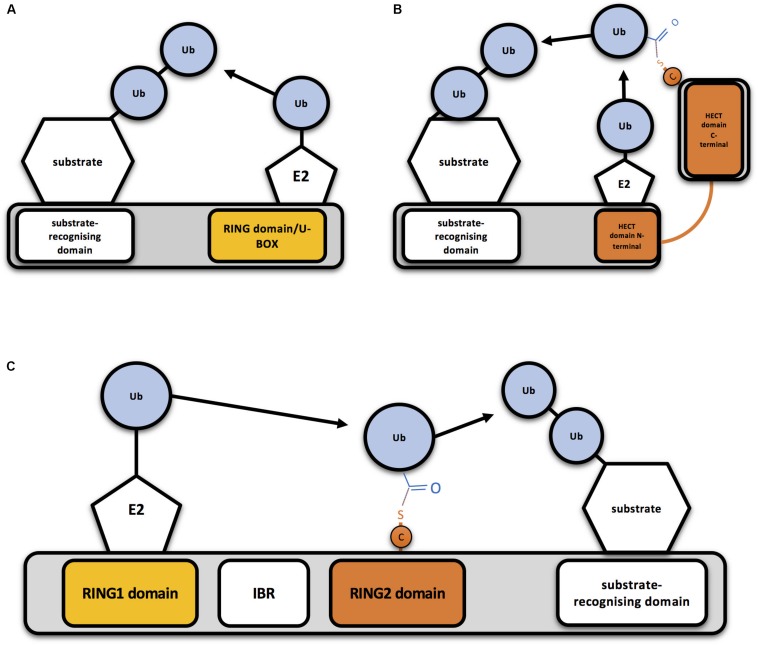
Mechanism of action of RING-, HECT- and RBR-type E3 ubiquitin ligases **(A)** Schematic representation of a RING-type ubiquitin E3 ligase. RING E3s bind both the E2-ubiquitin and the substrate to be ubiquitinated, so bringing them together allows direct conjugation of ubiquitin (Ub) on the substrate by the E2. A monomeric RING E3 ligase is shown for illustrative purposes. **(B)** Schematic representation of a HECT-type ubiquitin E3 ligase. Ubiquitin is transferred first to a cysteine (C) of the HECT domain through a thioester bond and then to the substrate. **(C)** Schematic representation of an RBR-type ubiquitin E3 ligase. Two RING domains are separated by an in-between-RING (IBR) domain. Ubiquitin is first transferred to a cysteine (C) of the second RING domain through a thioester bond and then to the substrate.

Eukaryotic cells express hundreds of ubiquitin E3 ligases, which can operate in different cellular contexts, respond to numerous cellular signals, and process diverse protein substrates (Zheng and Shabek, 2017). Ubiquitin E3 ligases have been classically classified in two different groups, based on conserved structural domains and the mechanism by which ubiquitin is transferred: RING (really interesting new gene)-type E3s and HECT (homologous to the E6AP carboxyl terminus)-type E3s. Whereas RING E3 ligases directly transfer the ubiquitin from the E2-ubiquitin complex to the substrate (Figure 1A), HECT-type E3s transfer ubiquitin to their own catalytic Cys before linking it to the substrate (Figure 1B; Deshaies and Joazeiro, 2009). Additionally, a third group of E3s, that combines features from both RING- and HECT-type E3 families, has been established: the RING between RING (RBR) family (Figure 1C). RBR and RING E3s share RING binding domains, but RBR family members have the ability to generate a thioester intermediate with ubiquitin, as HECT-type E3s do (Morreale and Walden, 2016).

Typically, one E3 ligase is able to modify several substrates, as well as to bind different E2s. The same protein can, therefore, be ubiquitinated by different E2/E3 combinations, which will lead to different ubiquitination patterns (Metzger et al., 2014). Substrate recognition by HECT-type E3 ligases depends on protein-protein interactions that are mediated by specific motifs typically located in the N-terminal of the HECT domain (Scheffner and Kumar, 2014). Substrate recognition by RING-type E3s is achieved either through regions of the E3 other than the RING domain, in the case of monomeric E3s, or through substrate recognition elements in other domains, in the case of multi-subunit RING E3s (Metzger et al., 2014). On the other hand, some studies have reported that substrate proteins have a short linear sequence, known as degron, important in the regulation of protein degradation rates. Not all degron are ubiquitin-dependent, but if they are, it appears that they facilitate the recognition of the substrate protein by the E3 ligase. Degrons can be modified by kinases and other enzymes. These modifications appear to be crucial for timing the interaction between E3 and substrate, even though they are not always necessary and many substrates of HECT-type E3s and CRLs are able to recognise their substrates in their native forms (Kanelis et al., 2001; Kamadurai et al., 2009; Rotin and Kumar, 2009; Fukutomi et al., 2014; Muńoz-Escobar et al., 2015). In order to increase the specificity toward their substrates, many E3 ligases, such as TRIMs, are able to form homo- and heterodimers and recognise multiple degrons located in the same substrate (Li et al., 2014). Moreover, the effect is summatory and a robust degron may have the same effect as two weak degrons (Welcker et al., 2013).

The role mediated by E3 ligases is so crucial, that their activity must be tightly controlled in order to ensure they solely act when necessary. Oligomerisation is one of the mechanisms that modulate the activity of HECT- and RING-type E3s. For instance, structural studies suggest that the trimeric arrangement of E6AP activates the ligase (Ronchi et al., 2014), whereas homodimerisation of the HECT domain of HUWE1 results in enzyme inactivity (Sander et al., 2017). RING-type E3s can act as independent enzymes, but most of them tend to form homo- or heterodimers, and even more complex multi-subunit assemblies in order to mediate ubiquitination (Metzger et al., 2014). For instance, RING E3 ligases cIAP, RNF4, BIRC7, IDOL, CHIP, and Prp19 homodimerize, and RING domains of both units interact with E2 proteins. By contrast, RING-type E3 ligases BRCA1-BARD1, Mdm2-MdmX, and RING1B-Bmi1 form heterodimers. While BRCA1 and Mdm2 have the ability to interact with E2 proteins, their partners do not. But they function as enhancers of ligase activity and interact with substrates (Brzovic et al., 2001; Joukov et al., 2001; Wang et al., 2004; Cao et al., 2005).

In this review we aim to provide a detailed description of mutations in ubiquitin E3 ligases, with the outlook that such detailed and structured catalog of mutants will provide a pattern to be considered by future researchers when designing new mutations on their E3 ligases.

## Mutations on Ring-Type E3 Ligases

**FIGURE 2:**
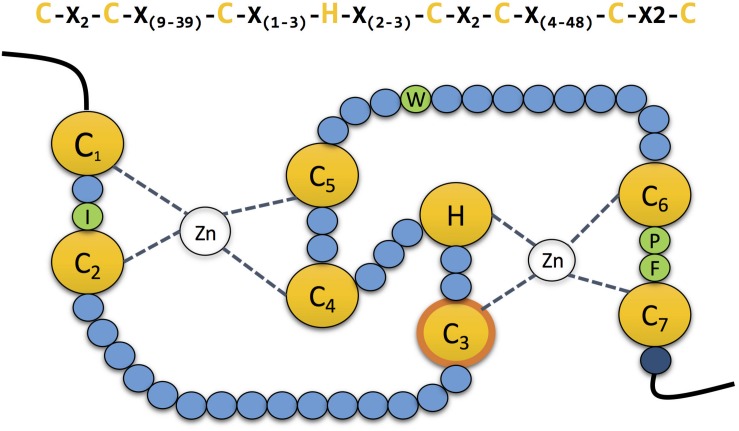
The zinc coordinating residues in RING domains. Schematic representation of the cross-brace” topology of RING domains. The RING domain contains seven conserved cysteines and one histidine (yellow) which are involved in the coordination of two atoms of zinc. The third cysteine mediates the ubiquitin transfer in the second RING domain in RBR E3 ubiquitin ligases (contour labelled in orange). Four conserved residues (green) guide the interaction with the E2 conjugating enzyme. Mutation of the last residue of the domain (dark blue), which is normally a positively charged arginine or lysine, compromises the stability of the adjacent cysteine, affecting the coordination of the zinc atom.

RING-type E3s are conserved from human to yeast. It is estimated that the human genome encodes above 600 different RING-type E3s. The RING domain was first characterised by Freemont et al. (1991). The canonical sequence for this 40–60 amino acid long domain is Cys-X_2_- Cys-X_(__9__–__39__)_-Cys-X_(__1__–__3__)_ - His- X_(__2__–__3__)_ -Cys-X_2_ -Cys- X_(__4__–__48__)_- Cys- X_2_-Cys. The conserved Cys residues (seven in total) and the single His are disposed in a “cross-brace” topology to coordinate two zinc ions and stabilise its structure (Figure 2; Deshaies and Joazeiro, 2009).

Initially, the role of RING domains was uncertain, although it was known they were involved in protein-protein interactions as well as in a wide range of cellular processes (Deshaies and Joazeiro, 2009). However, it was not until 1997 that the function of RING domains was elucidated by Bailly and co-workers (Bailly et al., 1997). Moreover, in 1999, Joazeiro and co-workers observed that the adapter protein c-Cbl bears two domains that act coordinately to mediate ubiquitination and subsequent degradation of substrates. Whereas the SH2 domain of c-Cbl served to recognize specific substrates, the RING domain was necessary to recruit and activate an ubiquitin-conjugating E2 (Joazeiro et al., 1999). After that, a similar role was conferred to a number of RING domain-containing proteins (Lorick et al., 1999). At present, it is accepted that the RING domain present in all RING E3s associates and activates E2-Ub conjugates promoting the direct transfer of ubiquitin from the E2 to the target protein (Figure 1A).

The interaction between the RING domain of E3 ligases and E2s was first elucidated with the crystal structure of Cbl’s RING domain bound to UbcH7 E2 (Zheng et al., 2000). The combination of many structural studies allowed the characterization of the four residues of each protein that play a crucial role in the interaction, those are shown in green in Figure 2. Located between Cys residues C_1_ and C_2_ of the RING domain, a hydrophobic residue (Ile, Leu or Val) interacts with two Pro residues from the E2. Those two prolines are localised in one of the two loops that compose the accessible surface of the E2 enzyme. Additionally, another hydrophobic residue (typically Trp, His or Leu) from the E3 interacts with a Phe and a Pro present on the second loop of the E2. Simultaneously, this Pro interacts with a Pro of the E3 located between Cys residues C_6_ and C_7_. Which in turn, is also connected to an Ala localised in the same loop of the E2. Finally, this same Ala of the E2 also interacts with a hydrophobic amino acid (typically Val, Phe or Ile) located straight after the Pro between C_6_ and C_7_ of the E3 (Deshaies and Joazeiro, 2009).

More recently, structural studies focused on RING-type E3:E2-Ub complexes have revealed the mechanism by which this class of ubiquitin ligases facilitates Ub transfer to substrate proteins. The E2-Ub complex has a flexible topology with multiple inter-domain configurations that are altered upon E3 binding (Pruneda et al., 2011). More precisely, binding of RING E3 reduces the dynamics of E2-Ub and stabilizes in an ensemble of closed conformations. This modification facilitates the reactivity for substrate Lys that can perform the corresponding nucleophilic attack (Pruneda et al., 2012; Soss et al., 2013). Studies carried out on dimeric E3s such as RNF4 or BIRC7 also support the same mechanism by showing that a positively charged residue (Arg or Lys) conserved in many RING E3s just straight after the last zinc-coordinating Cys supports the non-covalent interaction with the E2-Ub complex (Dou et al., 2012; Plechanovov et al., 2012).

As mentioned above, although some RING-type E3s act independently, they have the tendency to form homo- and heterodimers. Most RING-type E3s dimerise through their RING domain, such as RNF4 homodimers or MDM2/MDMX and BRAC1/BARD1 heterodimers (Brzovic et al., 2001; Linke et al., 2008; Liew et al., 2010). Nevertheless, there are exceptions. For instance, MARCH9 E3 ligase can form active dimers with RING-less variants (Hoer et al., 2007), whereas viral RING-type E3s MIR1 and MIR2 are believed to homodimerise via their transmembrane domain (Lehner et al., 2005). The tripartite motif (TRIM) family members in metazoans contain an additional domain termed B-box. Like the above mentioned RING domain, the B-box domain is a zinc-binding domain. However, whereas the RING domain is essential for E2 binding and E3 ligase activity, it has recently been shown that the B-box domain is involved in chain assembly rate modulation (Lazzari et al., 2019). Similarly, the U-box domain is also related to the RING domain, but unlike the B-box, it can interact with E2s. Additionally, the U-box domain has no coordinating zinc, so in order to ensure the stability of the structure, zinc-binding residues present in RING are replaced by charged and polar residues (Aravind and Koonin, 2000; Vander Kooi et al., 2006).

### Inactivating RING-Type E3s by Mutating the Zinc-Coordinating Residues

**FIGURE 3:**
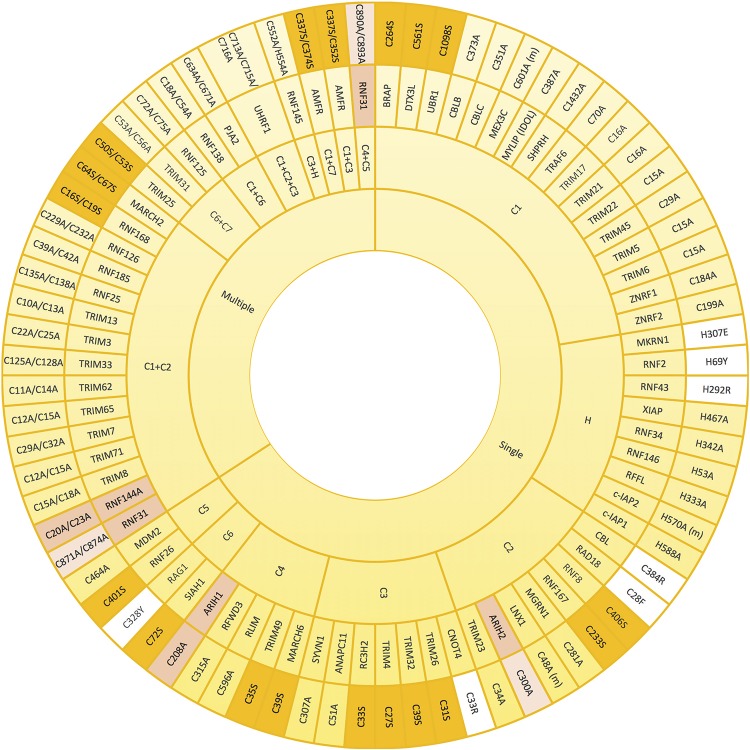
Mutations that alter zinc coordinating residues in RING domains. Wheel diagram showing the reported mutations in RING domains, classified first whether a unique (single) or various (multiple) residues were mutated simultaneously. Most of the inactive E3 enzymes have been obtained by mutating key residues into alanine (yellow). Lack of activity can also be acquired by mutations into serine (orange). Mutations into other residues have been also employed (white). Zinc coordinating residues of the first (pink) and the second (light pink) RING domains in RBR-type E3 ubiquitin ligases (pink) have also be modified in order to achieve inactivation. References to all the mutations shown in this figure are provided in Supplementary Table S1.

Since the coordination of the two atoms of zinc by the RING domain is crucial for E3 ligase activity, mutants that abolish such coordination have often been used to create ligase-dead versions of those E3 enzymes. Mutation of any of the conserved Cys and His involved in zinc binding should compromise the E3 activity, and so have all been, individually or jointly, mutated for that purpose (Figure 3). The mutated residue of choice to prevent E3 ligase activity appears the first conserved Cys (C_1_) of the RING domain, followed by the His (H), C_2_, C_3_, and C_4_. To our knowledge, C_7_ is the only key residue on the domain that has not been individually mutated for this purpose. However, it has been shown that simultaneous mutations on either C_1_+C_7_ or C_6_+C_7_ abolish the ligase activity of AMFR and some TRIM family members, respectively (Wang et al., 2014; Liu et al., 2017a; Lee et al., 2018a). As shown in Figure 3, many E3 ligases have been inactivated by simultaneous mutations on C_1_+C_2_. Less frequently, additional double mutations and even the triple C_1_+C_2_+C_3_ mutant have been efficiently applied to block the activity of distinct RING-type E3 ligases (Figure 3).

Zinc-coordinating Cys and His residues have been preferentially mutated into Ala in order to abolish the ubiquitin ligase activity of E3s (Figure 3). Nevertheless, in some cases, this type of substitution might be insufficient. In a recent research focused on studying TRIM27-dependent ubiquitination of UPS7, it was shown that a quadruple TRIM27 mutant, in which four zinc-coordinating residues of the RING domain (Cys16, Cys19, Cys31 and Cys33) were mutated into alanine, was still capable of ubiquitinating USP7. By contrast, the TRIM27 mutant, in which four zinc-binding residues of the B-box (Cys96, Cys99, His107, and Asp110) were simultaneously substituted by Ala, was incapable of ubiquitinating USP7 (not illustrated in Figure 3; Zaman et al., 2013). Moreover, it should be taken into account that in some cases a dominant negative effect may be acquired by the mutated E3 ligase. For example, CBL Cys381Ala mutant is not capable of ubiquitinating EGFR and thus, the subsequent desensitization of the receptor is abolished. However, CBL Cys381Ala mutant is still capable of interacting with EGFR, and consequently, competes with wild type CBL compromising CBL-mediated EGFR ubiquitination (Waterman et al., 1999). Similarly, the plant E3 ubiquitin ligase SINA1 mutant on the C_2_ of the RING domain Cys47Ser mutant retains dimerisation and substrate binding ability but lacks ubiquitination activity (den Herder et al., 2012).

Despite less frequently, in a number of investigations, the Cys involved in zinc coordination have also been efficiently mutated into serine. Indeed, this type of point mutation that results on E3 ligase inactivation has served to uncover, among others, the role of MDM2, RNF8, and SIAH1 RING E3s in cell cycle regulation, DNA damage response and Wnt signalling, respectively (Ji et al., 2017; Tian et al., 2017; Tripathi and Smith, 2017). Additionally, although there are fewer examples, it has been demonstrated that mutating the His into Glu, Tyr or Arg is sufficient to inactivate the ligase activity of MKRN1, RNF2, and RNF43 E3s, respectively (Xia et al., 2014; Loregger et al., 2015; Lee et al., 2018b; Figure 3). Similarly, it has been shown that mutating C_2_ of RAD18 and CBL into Phe and Arg, respectively, as well as substituting C_3_ of CNOT4 into Arg or C_6_ of RAG1 into Tyr has an inhibitory effect (Albert et al., 2002; Jones and Gellert, 2003; Williams et al., 2011; Javadi et al., 2013). It should be noted, however, that in search of structure-function relationships, the safest approach is to mutate into the smaller Ala residue (Fersht et al., 1999). Introducing larger residues might -in addition to preventing the coordination of the zinc- result in further distortions on the overall fold of the protein.

**FIGURE 4:**
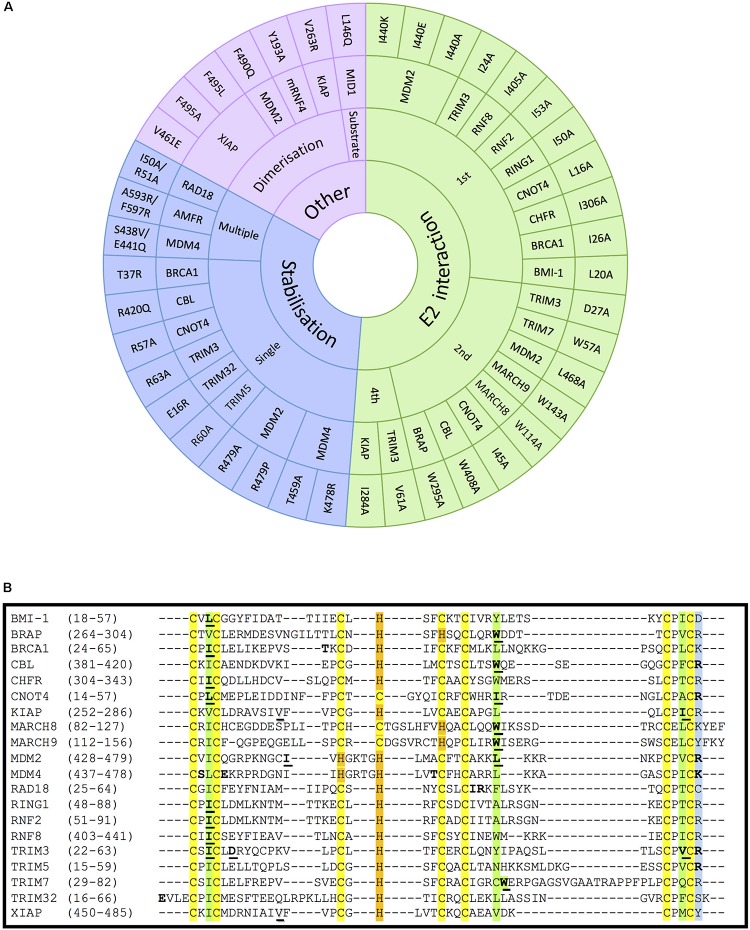
Mutations on RING- and RBR-type E3s that affect E2-interaction, domain stabilisation, protein dimerization or substrate recognition. **(A)** In RING-type E3 ubiquitin ligases, inactivation can be obtained by abolishing the interaction with E2 ubiquitin-conjugating enzymes (green). This has mostly been achieved by mutating the conserved 1st (I/L) and 2nd (W/I/L) hydrophobic residues indicated in Figure 2. Other mutations affecting the stabilisation of key residues of the domain (blue), dimerization or the interaction with a specific substrate also abolish the ligase activity (purple). For the stabilisation affecting mutations, those have been classified whether a unique (single) or various (multiple) residues were mutated simultaneously. References to all the mutations shown in this figure are provided in Supplementary Table S1. **(B)** Alignment of the RING domains of the RING-type E3 ligases involved in E2-interacting and stabilisation mutations within the RING domain. Conserved amino acids are highlighted in yellow and orange, respectively, for the Zn-coordinating Cys and His residues, and in green for the E2-interacting residues. The conserved positively charged residues at the end of the RING domain are highlighted in blue. Mutated E2-interacting residues are shown in bold and underlined. Mutated residues involved in stabilisation are shown in bold. Mutated residues involved in dimerisation are underlined and shadowed.

Especially in the absence of the molecular structure, deciding the residues that should be mutated might not always be straightforward, but appropriate sequence alignments can provide sufficient insight. For instance, TRIM37 has two adjacent Cys residues (Cys36 and Cys37) that could correspond to the C_4_ involved in zinc coordination (Supplementary Figure S1). Therefore, to ensure the inactivation of the enzyme, both Cys were simultaneously mutated (Kallijärvi et al., 2005; Wang et al., 2017). Similarly, ZNRF4 has two His nearby (His329 and His332) and in principle, either of them could be involved in coordinating zinc atoms. Once again, both His were mutated in order to obtain a catalytically inactive form of the E3 (Bist et al., 2017). Based on metal-binding studies, MDM2 His457 was initially confirmed to be the conserved His involved in zinc-coordination (Lai et al., 1998). Nevertheless, His452 is also essential, as demonstrated in auto-ubiquitination assays of this E3 ligase, with both His residues being necessary (Fang et al., 2000). It was later elucidated that His452 actually takes the place of the conserved Cys C_3_ in the zinc coordination, as illustrated in the sequence alignment in Figure 4B.

Additionally, there are few E3s bearing RING domains in which a non-conserved amino acid plays an indirect but pivotal role in the coordination of the zinc atom, and therefore, can be mutated in order to disrupt the activity of the ligase. For example, Thr455, which was originally believed to be directly involved in the zinc-coordination based on an incorrect primary sequence alignment, has been reported to abolish -upon its mutation- MDM2-dependent p53 ubiquitination (Boddy et al., 1994; Fang et al., 2000).

### Inactivating RING-Type E3s by Mutating the E2-Interacting Residues

It has previously been described that RING E3s interact with E2-Ub conjugates via their RING domain to directly transfer the ubiquitin to the substrate protein. Therefore, disrupting the interaction between E2s and RING-type E3s has also been extensively used to block, or at least reduce ubiquitination mediated by RING E3s. All three key hydrophobic residues on E3s that mediate the interaction with E2s (shown in green in Figure 2) have been recurrently mutated to compromise the activity of the E3s. As shown in Figure 4A, numerous RING-type E3 ligases have been successfully inactivated by mutating the first Ile/Leu, the second Trp/Leu or the last Ile/Val into Ala. The first Ile/Leu has been mutated in BRCA1, BMI-1, CHFR, CNOT4, RING1, RNF2, RNF8 and TRIM3 (Albert et al., 2002; Eakin et al., 2007; Alchanati et al., 2009; Kim et al., 2010; Mallette et al., 2012; Raheja et al., 2014; Liu et al., 2018; Shen et al., 2018). The second Trp/Leu was mutated abolishing ligase activity in BRAP, CBL, MARCH8, MARCH9, MDM2, and TRIM7 (Joazeiro et al., 1999; Chen et al., 2012; Hayes et al., 2012; Chakraborty et al., 2015; Fan and Wang, 2017; Tan et al., 2019). Finally, the last Ile/Val was successfully mutated in KIAP and TRIM3 (Dou et al., 2012; Raheja et al., 2014). All these hydrophobic residues are conserved as seen in Figure 4B. However, to our knowledge, no one has mutated the E2-interacting Pro (located between C6 and C7) with the aim to disrupt the association with the E2 enzyme. Given the special properties of this cyclic amino acid, one certainly would have to be weary of additional conformational effects that could be caused by its mutation to Ala. Additionally, MDM2 mutant variants Ile440Glu and Ile440Lys prevent MDM2-dependent ubiquitination of p53, by disrupting the E2–ubiquitin binding by the E3 ligase without altering its RING domain structure (Nomura et al., 2017). This residue, however, is barely conserved across the different RING domains.

However, other types of mutations have also been efficiently applied to disrupt the interaction between E2s and E3s. For instance, one of the few U-box-type E3s that has been mutated is CHIP, also known as STUB1, which was inactivated by substituting His260 into Glu (Seo et al., 2018). Likewise, the U-box domain-containing UBE4B E3 can be inactivated by mutating a Pro (Pro1140) that is conserved among U-box-type E3 ligases (Pro269 in CHIP) into Ala (not included in Figure 4; Okumura et al., 2004; Li et al., 2018).

### Inactivating RING-Type E3s by Disrupting Substrate Recognition, E3 Dimerization and Stability

Many RING-type E3 ligases possess a conserved positively charged residue (Arg or Lys) in the last position of the RING domain, which appears to be essential for the ubiquitination activity of the E3. Nevertheless, it is still controversial whether the effect of mutating this residue results from the impaired interaction with E2s or from destabilization of the RING domain (Figure 4A, included in stabilization) (Albert et al., 2002; Linke et al., 2008; Lienlaf et al., 2011; Dou et al., 2012; Raheja et al., 2014; Nomura et al., 2017). But this uncertainty is not surprising given that mutations have been generated to substitute the positively charged residue by a very diverse choice of residues (mostly to Ala, but also to Glu, Pro and even Arg, as can be seen in Figure 4A. Future studies should preferably limit the mutations to substituting the positively charged residue by Ala.

As shown in Figure 4A, a number of other single point mutations, as well as multiple point mutations, have been generated along different positions of the RING domain to compromise protein stability and hence, E3 ligase activity, but no clear pattern can be predicted based on the studies reported so far. For example, the Tyr37Ala mutant in BRCA1 lack ligase activity, being therefore incapable of reversing γ-radiation hypersensitivity of BRCA1-null human breast cancer cells (Ruffner et al., 2001). In the case of the RAD18 ligase, the Ile50Ala/Arg51Ala inactive mutant allowed to study the formation of ternary complexes with RAD6A (Masuda et al., 2012); these two residues were selected due to being highly conserved among species.

RING-type E3s that act as dimers can also be inactivated by preventing their dimerization process. For instance, mutation of Val461Glu and Val263Arg within the RING domain diminishes oligomerisation and activity of XIAP and KIAP ligases, respectively (Poyurovsky et al., 2007; Dou et al., 2012; Nakatani et al., 2013). In other cases, however, the dimerization affecting residues are immediately after the RING domain (Supplementary Figure S2), as revealed for example by the mutation Phe490Gln in MDM2 (Poyurovsky et al., 2007). Another approach consists of inactivating oligomeric E3 ligases without affecting the oligomerisation process itself. For example, RNF4 Val134Ala and Ile153Ala mutants can form dimers but are catalytically incapacitated (Liew et al., 2010; Dou et al., 2012). Similarly, other E3 ligase mutants have been shown to act in a dominant negative due to their homo-dimeric nature. For example, mutant Fbw7 has a dominant-negative effect when dimerising with wild-type Fbw7, being able to effectively bind their substrate MYC but not to ubiquitinate and degrade it (Welcker et al., 2013).

Several experiments have also been carried out mutating specific residues on E3 ligases that are critical for the interaction with a given substrate, such as Leu146Gln mutation on the B-box containing E3 MID1 that cannot associate, nor ubiquitinate its substrate PP2A alpha-2 (Du et al., 2013; Figure 4).

## Mutations on Hect Type E3 Ligases

The human HECT-type E3 family consists of 28 members that are divided into three different groups depending on their N-terminal domain architecture: (i) the *NEDD4 subfamily*, characterized by containing a C2 domain, a HECT domain and two to four WW domains, which bind to the PY motifs of target proteins (Staub et al., 1996; Kanelis et al., 2001); (ii) the *HERC subfamily*, which integrates at least one regulator chromosome condensation 1 (RCC1)-like domain (RLDs) and a reduced HECT domain; and (iii) the *other HECT subfamily*, that embrace HECT-type E3s not fitting the above mentioned two subfamilies.

Despite those differences, all HECT-type E3s share a ∼350 amino acid long HECT domain, that was first described in human papilloma virus E6 associated protein (E6AP) (Huibregtse et al., 1995). In the HECT domain, a conserved Cys forms thioester-linked-intermediate complexes with ubiquitin (Figure 1B), before being transferred and attached to the substrate through a transthiolation reaction. This conserved Cys is located in the C-terminal region of the HECT domain, while the E2 interacting site is localised in the N-terminal site (Figure 1B; Rotin and Kumar, 2009).

### Inactivating HECT-Type E3s

**FIGURE 5:**
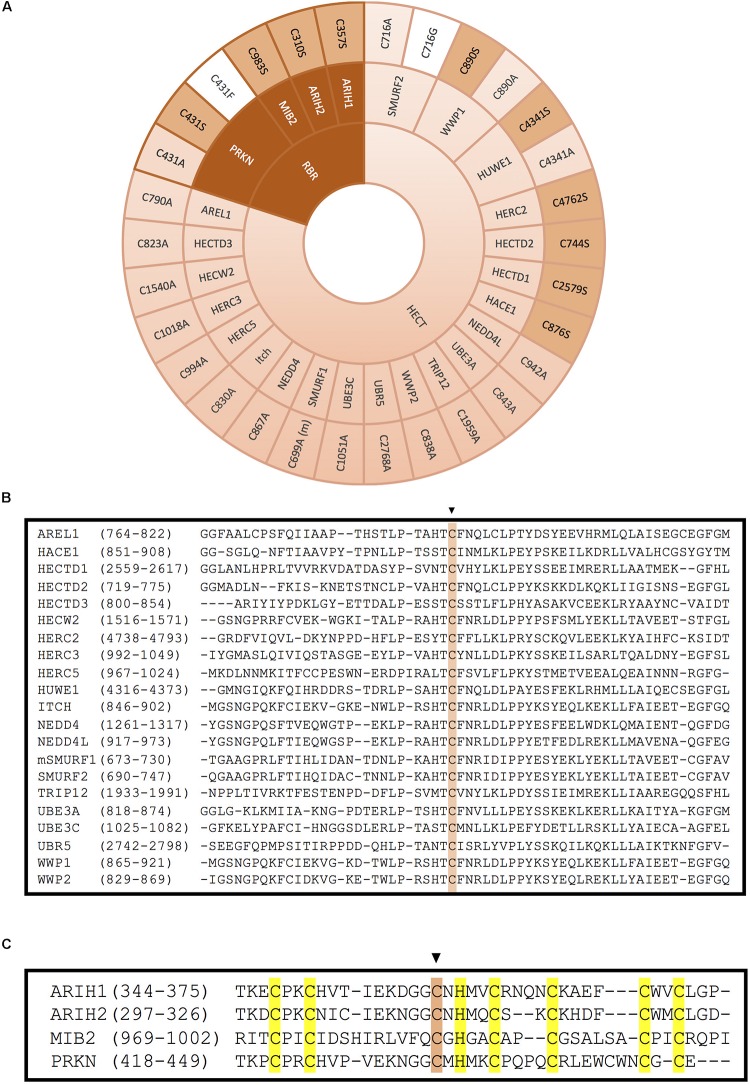
Inactivation of HECT- and RBR-type E3 ligases by mutation of the catalytic cysteine. **(A)** Mutation of the catalytic cysteine into an alanine (light pink), a serine (pink) or another residue (white) abolishes transference of ubiquitin onto the substrate. Inactivation of the catalytic cysteine of RBR-type E3 ubiquitin ligases is obtained by mutation of the third conserved cysteine in the second RING domain. (m) indicates that this mutation has been done in the mice homolog of the protein. References to all the mutations shown in this figure are provided in Supplementary Table S1. **(B)** Alignment of the HECT domains of the HECT-type E3 ligases. The conserved catalytic cysteine is highlighted in pink. **(C)** Alignment of the RING2 domains of the RBR-type E3 ligases. Conserved zinc-coordinating cysteines and histidine on the second RING domain of mutated RBR E3 ligases are highlighted in yellow and the mutated catalytic cysteine is highlighted in pink.

Given that an *active site Cys* is required for the formation of a thioester intermediate with ubiquitin, a typical approach is to mutate this specific Cys to generate ligase dead versions of HECT E3 ligases. As shown in Figure 5A, the majority of HECT-type E3 ligases have been inactivated by replacing this catalytic Cys by Ala. This approach has served to unveil, among others, the involvement of HERC3 in immune response (Hochrainer et al., 2015), the role of NEDD4L in EnaC receptor recycling (Zhou et al., 2007), and the contribution of SMURF1 to Axin degradation (Fei et al., 2013).

Less frequently, some ligase dead HECT-type E3s have been generated by substitution of the active Cys into Ser (Figure 5A). It has been reported that when the catalytic Cys of an E3 is mutated into Ser, the residue is still capable of binding through an oxyester bond with ubiquitin, but incapable to transfer it to substrates, which might result in a dominant-negative effect. In ubiquitination assays employing this type of ligase dead E3s, a stable monoubiquitinated version of the E3 has been detected (Lee et al., 2014). This approach has allowed, among other things to discover many substrates of distinct HECT-type E3 ligases. For instance, it was found that wild type version of HACE1 could ubiquitinate and target for degradation the small GTPase Rac1, but the Cys876Ser ligase dead version of the E3 ligase could not (Torrino et al., 2011). Similarly, HERC2 C4762S and HUWE C4341S mutants failed to ubiquitinate their substrates BRCA1 and N-Myc, respectively (Zhao et al., 2008; Wu et al., 2010). The sequence alignment for all the HECT domain E3 ligases illustrated in Figure 5A is shown around the catalytically active Cys in Figure 5B.

## Mutations on Rbr Type E3 Ligases

RING between RING family members contain two RING domains (RING1 and RING2) that are separated by an in-between-RING (IBR) zinc-binding domain. Morett and Bork first characterised these domains in 1999 in a sequence profile-based characterisation (Morett and Bork, 1999). In the process of confirming reports that UbcH7 could also interact with RBR E3s, they discovered that these RBR E3s act as RING/HECT hybrids. The first RING domain serves as the E2 binding platform, while the C_3_ of the second RING serves as the active site that mediates ubiquitination similarly to HECT E3 ligases (Wenzel et al., 2011; Figures 1C, 5C).

### Inactivating RBR-Type E3s

As it happens with HECT-type E3, the mutation of the catalytic Cys in the RING2 of RBR E3s results in the inactivation of these enzymes. However, unlike in HECT-type E3 ligases, in RBR E3s the active Cys has been mostly substituted by Ser, and less by Ala (Figure 5A). For example, C983S substitution in MIB2 resulted in ligase inactivation, and therefore, prevented ubiquitination of its substrate TANK-binding kinase 1 (Ye et al., 2014). Similarly, mutating the active Cys of ARIH2 (also called TRIAD1) into Ser or Ala completely abolished autoubiquitination of the RBR-type E3 ligase. Parkison disease has been shown to develop in patients carrying a Cys431Phe mutation at the catalytic Cys of the RBR-type E3 ligase PRKN; those mutants have also been characterized in the lab (Sarraf et al., 2013), in addition to the more common substitutions to Ser and Ala (Liu et al., 2017b; Xin et al., 2018).

In order to generate ligase dead versions of RBR-type E3s, it has been also shown to be plausible to preserve the active Cys, and instead mutate the zinc-coordinating residues in either of the two RING domains, substituting by Ala one or several of those key residues. For instance, ARIH1 and RNF144A have been successfully inactivated by modifying their RING1 domain (Figure 3, dark pink). Whereas mutating C4 of ARIH1 (Cys208) was sufficient to inhibit the ligase, Cys20 and Cys23 (C1+C2) were simultaneously modified to block the catalytic activity of the RBR-type E3 RNF144A (Ho et al., 2014; von Stechow et al., 2015). On the contrary, ARIH2 and RNF31 have been inactivated by mutating their RING2 domain zinc-coordinating Cys residues (Figure 3, light pink). Cells expressing an ARIH1 mutant in which the C2 of the RING2 domain was mutated into Ala (ARIH2 Cys300Ala mutant) was no longer able to ubiquitinate NLRPL3 (Kawashima et al., 2017). Similarly, Smit and co-workers generated various ligase dead versions of RNF31 by mutating simultaneously Cys871 and C874 (C1+C2) or Cys890 and Cys892 (C4+C5) of the RING2 domain (Smit et al., 2012).

## Conclusion

Mutations on E3 ligases have been associated with a number of diseases, including neurological disorders (George et al., 2018; Osinalde et al., 2019). Thus, understanding their mechanism of action, as well as identifying which substrates are regulated by each E3 at different developmental stages and cell types, will provide invaluable knowledge that might contribute to develop therapeutic strategies to treat these diseases. Generation of E3 ligase dead mutants can certainly provide crucial information for this purpose. While the use of gene silencing techniques might be more appropriate to study the phenotypes derived from the loss of function of E3 ligases, the overexpression of ligase death versions can provide information about (i) the E2 enzymes they work with, (ii) substrate recognition domains and (iii) existing mechanism that regulate their activity. Additionally, a number of biochemical experiments do benefit from comparing the ectopic expression of wild type active E3 ligases with their mutated inactive variants.

As evident from all the examples shown in this review, there are multiple options to disrupt the activity of an E3 ligase. As illustrated by the sequence alignment in Figure 4B, the first necessary step is to identify which are the key residues in our ligase of interest. This is an essential step to ensure that any mutagenesis performed has a higher chance of success in disrupting the E3 ligase activity. For example, not all cysteine residues within a RING domain are involved in zinc coordination, as can be seen in the sequence alignment of Mdm2 in Figure 4B. When this cysteine of the Mdm2 RING domain was mutated (Kostic et al., 2006) the zinc coordination was maintained and no disruption to the ubiquitination activity of Mdm2 was detectable.

It is worth mentioning that mutating key residues involved either in the coordination of the zinc ions, dimerisation, proteins stabilization or E2 interaction might not always be sufficient to abolish the catalytic activity of the E3 ligase. The resulting mutation replacing the original residue that is substituted can actually be determinant in order to have a functional effect. For instance, mutating Phe495 of XIAP into either Ala, Tyr or Trp completely prevents E3 ligase autoubiquitination. However, XIAP Phe495Leu mutants appear to be functionally wild-type like (Nakatani et al., 2013); but might not be that surprising given the partial hydrophobic similarity between those two amino acids.

As illustrated within this review, so far one of the most frequent approaches for RING E3 ligases has been to mutate the residues involved in the zinc coordination (Cys and His residues, shaded in yellow and orange, respectively, in Figure 4B). Eliminating the zinc coordination on the RING domain is well known to severely disrupt the ubiquitination activity of those E3 ligases. However, this breakdown of the global structural integrity of the RING construct might lead to a severe effect in the folding and expression levels of the E3 ligase (Chasapis et al., 2010). Therefore, for certain experiments might be more effective to generate less disruptive point mutations. For example, the mutation of the hydrophobic residues (Ile, Leu, Trp, Val, shaded in green in Figure 4B) that mediate the interaction with the E2 conjugating enzyme, as demonstrated for a number of RING E3 ligases. To our knowledge this approach has not yet been employed for the E2-interacting RING domain of RBR E3 ligases, but it should indeed be an interesting experiment to perform.

Another approach that has been used as well is to eliminate by mutagenesis the positive charge of the Lys or Arg residue located straight after the last zinc-coordinating Cys of the RING domain. It is yet unclear, however, whether the effect caused by this mutation is on the interaction with E2s or from destabilization of the RING domain.

Mutations on the active Cys of HECT- and RBR-type E3 ligases are very straight forward, as they generate, without further effect to the structure and stability of the E3, ligase-dead versions of these enzymes. Those are of good value to be used as the best control in experiments overexpressing the wild type ligase, for example, to identify substrates in an unbiased manner. Additionally, if mutating the active site Cys to Ser, the formation of an oxyester to ubiquitin can be used with the aim to obtain a dominant-negative version of the ligase; the E3 will recruit the E2 and the substrate but the ubiquitination reaction cannot proceed since the ubiquitin cannot be released once it has conjugated to the E3.

To investigate the regulation of a specific protein by a particular HECT or RBR E3, however, it might be more suitable to mutate the ligase at the substrate recognition motif. Moreover, in some cases, as is the case of some RING E3s, the inactivation of E3 enzymes is not achieved by a single point mutation, even though such residue is defined as a key amino acid involved in substrate recognition. Hence, in such situations, several residues must be simultaneously mutated in order to disrupt the E3 ligase function. The generation and usage of E3 mutants have revealed unexpected and important lessons about the complexity of this family of enzymes. Nevertheless, a complete understanding of E3 ligases still requires more research, in which the generation of novel E3 ligase mutants will undoubtedly be decisive.

## Author Contributions

CG-B designed and wrote the first draft. All authors contributed to this manuscript.

## Conflict of Interest

The authors declare that the research was conducted in the absence of any commercial or financial relationships that could be construed as a potential conflict of interest.
