# Supplementary figures and images for: How to Inactivate Human Ubiquitin E3 Ligases by Mutation
Source: Front Cell Dev Biol. 2020 Feb 4;8:39. doi: 10.3389/fcell.2020.00039 (PMC7010608; doi:10.3389/fcell.2020.00039)

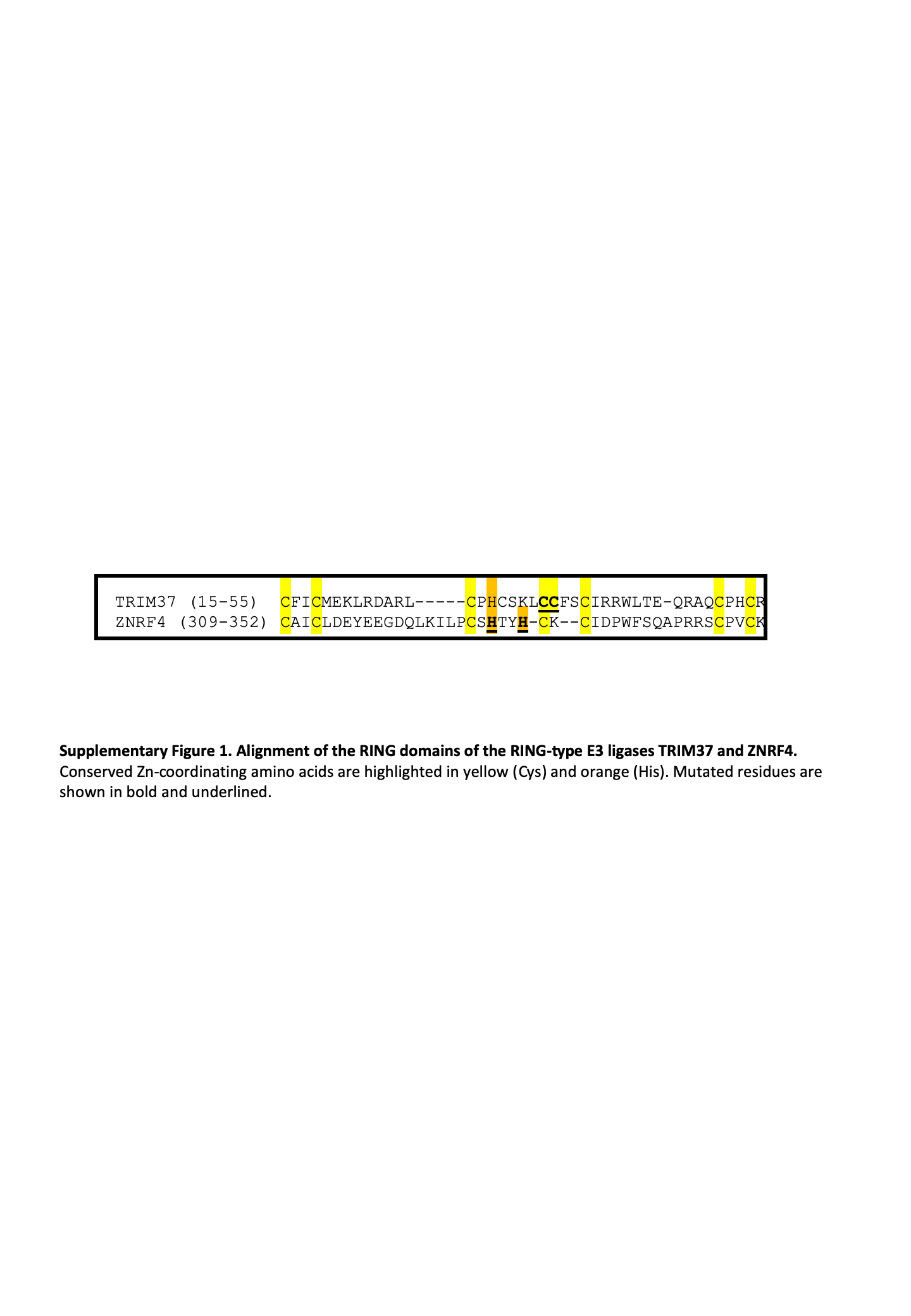

Supplement: Supplementary file 1 [file Image_1.jpg]

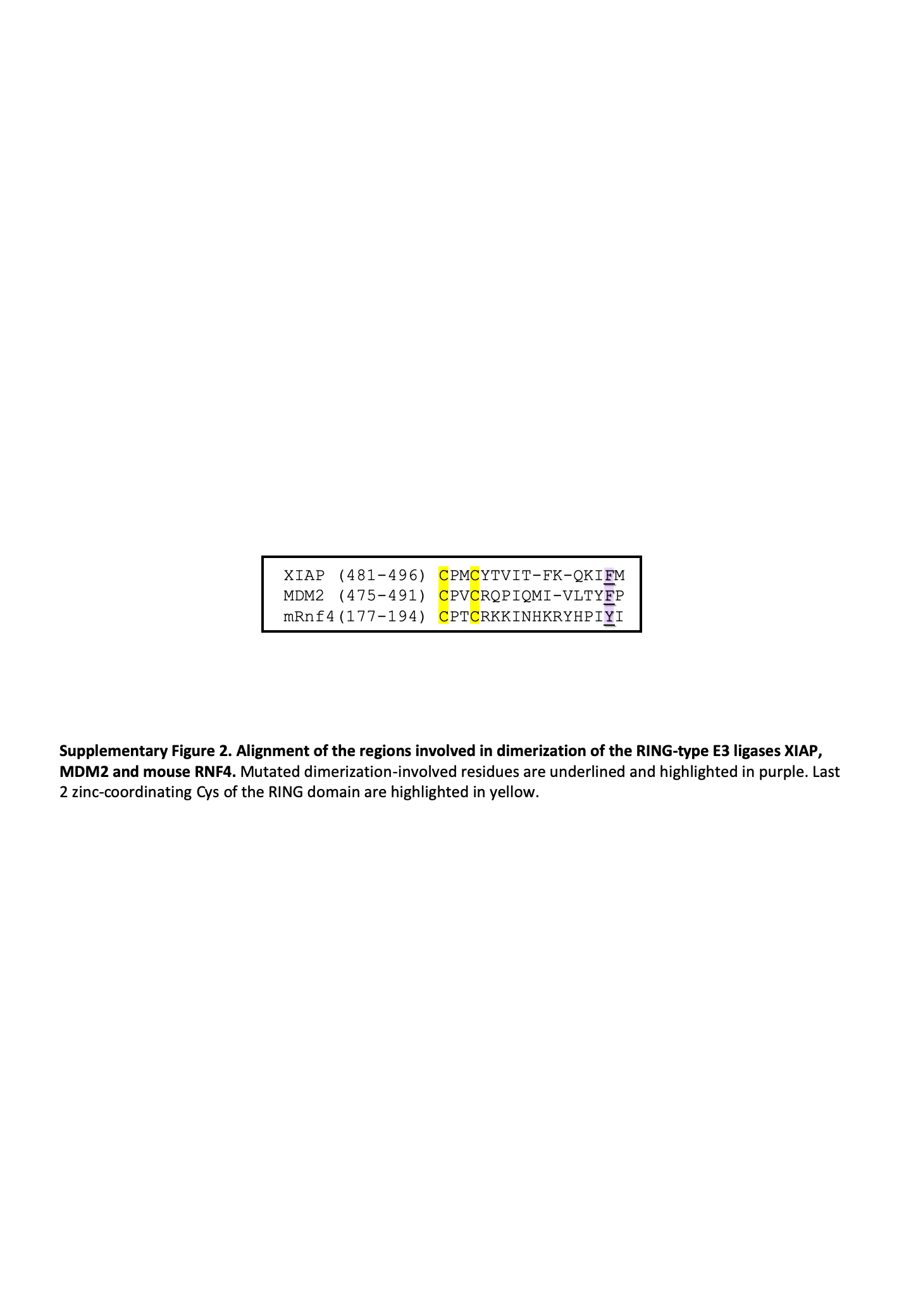

Supplement: Supplementary file 2 [file Image_2.jpg]
