# Supplementary material for: How to Inactivate Human Ubiquitin E3 Ligases by Mutation
Source: Front Cell Dev Biol. 2020 Feb 4;8:39. doi: 10.3389/fcell.2020.00039 (PMC7010608; doi:10.3389/fcell.2020.00039)
Supplement: Supplementary file 3 [file Table_1.DOCX]

Supplementary table 1. **Inactivation of E3 ubiquitin ligases by mutation**. The residues of the domain are specified in brackets. (m) indicates that the mutations were performed in the mouse version of the protein. E2: an E2-interacting residue was mutated. Zn: one of the seven cysteines or the histidine that coordinated the atoms of Zn was mutated. Catalytic cysteine: the catalytic cysteine of the HECT domain was mutated. *specifies that the catalytic cysteine is from the RING2 domain in RBR-type E3 ligases. On each reference it is specified the substrate that was analysed.

| Protein name | Uniprot Ref (human) | E3 type | Reference | Mutation | Type of mutation | Substrate |
| --- | --- | --- | --- | --- | --- | --- |
| AMFR | [Q9UKV5](http://www.uniprot.org/uniprot/Q9UKV5) | **Ring (341-379)** | (W. Liu, Shang, and Li 2014) | A593R/F597R | **E2** |  |
|  |  |  | (Q. Wang et al. 2014) | C337S/C352S; C337S/C374S (m) | **Zn** | STING |
|  |  |  | (Ying et al. 2009) | C341G | **Zn** | SOD1; Ataxin-3 |
| ANAPC11 | [Q9NYG5](http://www.uniprot.org/uniprot/Q9NYG5) | **Ring (34-77)** | (Gmachl et al. 2000) | C51A | **Zn** | Securin; cyclin B |
| AREL1 | [O15033](http://www.uniprot.org/uniprot/O15033) | **HECT (483-823)** | (J. Bin Kim et al. 2013) | C790A | **Catalytic cysteine** | SMAC; HtrA2; ARTS |
| ARIH1 | [Q9Y4X5](http://www.uniprot.org/uniprot/Q9Y4X5) | **Ring 1 (186-236) IBR (256-317) Ring 2 (344-375)** | (Ardley et al. 2001) | C208A | **Zn** |  |
|  |  |  | (Duda et al. 2013) | F430A; E431A; E503A | **OTHER** |  |
|  |  |  | (Kelsall et al. 2013) | C357S | **Catalytic cysteine*** |  |
|  |  |  | (Scott et al. 2016) | C357S | **Catalytic cysteine*** | CRL substrates |
|  |  |  | (von Stechow et al. 2015) | C208A | **Zn** | 4EHP |
|  |  |  | (Wenzel et al. 2011) | C357S | **Catalytic cysteine*** |  |
| ARIH2 | [O95376](http://www.uniprot.org/uniprot/O95376) | **Ring 1 (139-188) IBR (208-270) Ring 2 (297-326)** | (Kawashima et al. 2017) | C300A; H158A | **Zn** | NLRP3 |
|  |  |  | (Kelsall et al. 2013) | C310S | **Catalytic cysteine*** |  |
|  |  |  | (Marteijn et al. 2007) | H158A | **Zn** | ¬Gf1 |
| c-IAP1 | [Q13490](http://www.uniprot.org/uniprot/Q13490) | **Ring (571-606)** | (Blankenship et al. 2009) | H588A | **Zn** | Auto |
|  |  |  | (Conze et al. 2005) | H582A (m) | **Zn** | c-IAP2 |
|  |  |  | (Li X, Yang Y, and Ashwell JD. 2002) | H588A | **Zn** | TRAF2 |
|  |  |  | (L. Xu et al. 2007) | H588A | **Zn** | MAD1 |
|  |  |  | (Y. Zhao et al. 2007) | H588A | **Zn** | ASK1 |
| c-IAP2 | [Q13489](https://www.uniprot.org/uniprot/Q13489) | **Ring (557-592)** | (Conze et al. 2005) | H570A (m) | **Zn** | Auto |
|  |  |  | (Conze, Zhao, and Ashwell 2010) | H570A (m) | **Zn** |  |
|  |  |  | (Giardino Torchia et al. 2015) | H570A (m) | **Zn** |  |
| KIAP | [Q96CA5](https://www.uniprot.org/uniprot/Q96CA5#family_and_domains) | **Ring (252-286)** | (Dou et al. 2012) | I284A | **E2** |  |
|  |  |  |  | R286A | **OTHER** |  |
|  |  |  |  | F296H | **OTHER** |  |
|  |  |  |  | V263R | **OTHER** |  |
| BMI-1 | [P35226](https://www.uniprot.org/uniprot/P35226) | **Ring (18-57)** | (Alchanati et al. 2009) | L20A | **E2** |  |
| BRAP | [Q7Z569](https://www.uniprot.org/uniprot/Q7Z569) | **Ring (264-304)** | (Hayes et al. 2012) | W295A | **E2** | Auto; USP15 |
|  |  |  | (Shoji et al. 2017) | C264S | **Zn** |  |
| BRCA1 | [P38398](https://www.uniprot.org/uniprot/P38398) | **Ring (24-65)** | (Eakin et al. 2007) | I26A | **E2** | ERα |
|  |  |  |  | C61G; C64G | **Zn** | ERα |
|  |  |  | (Fabbro and Henderson 2008) | C61G | **Zn** |  |
|  |  |  |  | C61G | **Zn** |  |
|  |  |  | (Morris and Solomon 2004) | C61G | **Zn** |  |
|  |  |  | (Nelson and Holt 2010) | C61G | **Zn** |  |
|  |  |  | (Nishikawa et al. 2004) | C61G | **Zn** |  |
|  |  |  | (Ruffner et al. 2001) | C61G; C64G; C39Y; C24R | **Zn** |  |
|  |  |  |  | T37R | **OTHER** |  |
|  |  |  | (Sankaran et al. 2006) | I26A | **E2** |  |
|  |  |  | (Stewart et al. 2017) | L63A; K65A | **OTHER** |  |
|  |  |  |  | I26A | **E2** |  |
|  |  |  |  | C61G | **Zn** |  |
| CBL | [P22681](https://www.uniprot.org/uniprot/P22681#names_and_taxonomy) | **Ring (381-420)** | (Bulut et al. 2013) | ΔY368; ΔY371 | **OTHER** | PI3K |
|  |  |  | (Duyvestyn et al. 2014) | C379A (m) | **Zn** |  |
|  |  |  | J(Javadi et al. 2013) | C384R | **Zn** |  |
|  |  |  |  | Y371H | **OTHER** |  |
|  |  |  | (Joazeiro et al. 1999) | W408A | **E2** | RPTKs |
|  |  |  |  | C381A | **Zn** | RPTKs |
|  |  |  | (Levkowitz et al. 1999) | C381A | **Zn** | EGFR |
|  |  |  | (Lv et al. 2017) | C381A | **Zn** | JAK2 |
|  |  |  | (Miura-Shimura et al. 2003) | Y700F | **OTHER** | Vav |
|  |  |  | (Molero et al. 2006) | C379A | **Zn** |  |
|  |  |  | (Oshikawa et al. 2011) | R420Q | **OTHER** | Flt3-ITD |
|  |  |  | (Rathinam et al. 2010) | C379A | **Zn** |  |
|  |  |  | (Taylor et al. 2015) | C379A | **Zn** | FLT3 |
|  |  |  | (Thien, Walker, and Langdon 2001) | W408A; C381A; ΔC381; H398A; ΔH398 | **Zn** | EGFR |
|  |  |  |  | Y371F; ΔY368; ΔY371 | **OTHER** | EGFR |
|  |  |  | (Thien et al. 2005) | C381A | **Zn** |  |
|  |  |  | (Waterman et al. 1999) | C381A | **Zn** | EGFR |
|  |  |  | (Xiong et al. 2011) | C381A | **Zn** | CSF-1R |
| CBLB | [Q13191](https://www.uniprot.org/uniprot/Q13191#sequences) | **Ring (373-412)** | (Bachmaier et al. 2007) | C373A | **Zn** |  |
|  |  |  | (Ettenberg et al. 2001) | C373A | **Zn** |  |
|  |  |  | (Oksvold et al. 2008) | C373A | **Zn** |  |
|  |  |  | (Oshikawa et al. 2011) | C373A | **Zn** | Flt3-ITD |
|  |  |  | (Rathinam et al. 2010) | C373A | **Zn** |  |
| CBLC | [Q9ULV8](https://www.uniprot.org/uniprot/Q9ULV8#sequences) | **Ring (351-390)** | (M. Kim et al. 2004) | C351A | **Zn** | SRC |
| CHFR | [Q96EP1](https://www.uniprot.org/uniprot/Q96EP1) | **Ring (304-343)** | (J. M. Kim et al. 2010) | I306A | **E2** | HLTF |
| CNOT4 | [O95628](https://www.uniprot.org/uniprot/O95628) | **Ring (14-57)** | (Albert et al. 2002) | C17A; C33R | **Zn** |  |
|  |  |  |  | L16A; I45A | **E2** |  |
|  |  |  |  | R57A | **OTHER** |  |
| DTX3L | [Q8TDB6](https://www.uniprot.org/uniprot/Q8TDB6) | **Ring (561-600)** | (Holleman and Marchese 2014) | C561A/C596A/C599A | **Zn** | CXCR4 |
|  |  |  | (Yong Zhang et al. 2015) | C561S; C564S | **Zn** | H2BJ |
| HACE1 | [Q8IYU2](https://www.uniprot.org/uniprot/Q8IYU2) | **HECT (574-909)** | (Anglesio et al. 2004) | C876S | **Catalytic cysteine** |  |
|  |  |  | (Hollstein et al. 2015) | C876S | **Catalytic cysteine** |  |
|  |  |  | (Palicharla and Maddika 2015) | C876S | **Catalytic cysteine** | YB-1 |
|  |  |  | (Torrino et al. 2011) | C876S | **Catalytic cysteine** | Rac1 |
| HECTD1 | [Q9ULT8](https://www.uniprot.org/uniprot/Q9ULT8) | **HECT(2151-2610)** | (Sarkar and Zohn 2012) | C2579G | **Catalytic cysteine** | Hsp90 |
|  |  |  | (Sugrue et al. 2019) | C2579G | **Catalytic cysteine** | RARA |
|  |  |  | (Tran et al. 2013) | C2579G | **Catalytic cysteine** | APC |
| HECTD2 | [Q5U5R9](https://www.uniprot.org/uniprot/Q5U5R9) | **HECT (437-776)** | (Coon et al. 2015) | C744S | **Catalytic cysteine** | PIAS1 |
| HECTD3 | [Q5T447](https://www.uniprot.org/uniprot/Q5T447) | **HECT (512-857)** | (Y. Li et al. 2013) | C823A | **Catalytic cysteine** | Caspase-8 |
|  |  |  | (F. Li et al. 2018) | C823A | **Catalytic cysteine** | TRAF3 |
|  |  |  | (J. Yu et al. 2008) | C539A | **Catalytic cysteine** | TARA |
| HECW2 | [Q9P2P5](https://www.uniprot.org/uniprot/Q9P2P5) | **HECT (1237-1572)** | (K. Choi et al. 2016) | C1540A | **Catalytic cysteine** | AMOTL1 |
|  |  |  | (Krishnamoorthy, Khanna, and Parnaik 2018) | C1540A | **Catalytic cysteine** | PCNA; lamin B1 |
| HERC2 | [O95714](https://www.uniprot.org/uniprot/O95714) | **HECT (4457-4794)** | (Chan et al. 2014) | C4762S | **Catalytic cysteine** | USP33 |
|  |  |  | (Kühnle et al. 2011) | C4762S | **Catalytic cysteine** |  |
|  |  |  | (Wu et al. 2010) | C4762S | **Catalytic cysteine** | BRCA1 |
| HERC3 | [Q15034](https://www.uniprot.org/uniprot/Q15034) | **HECT (951-1050)** | (Cruz et al. 2001) | C1018A | **Catalytic cysteine** |  |
|  |  |  | (Hochrainer et al. 2015) | C1018A | **Catalytic cysteine** | RelA |
| HERC5 | [Q9UII4](https://www.uniprot.org/uniprot/Q9UII4) | **HECT (702-1024)** | (Kroismayr et al. 2004) | C994A | **Catalytic cysteine** |  |
|  |  |  | (Shi et al. 2010) | C994A | **Catalytic cysteine** | IRF3 |
|  |  |  | (Wong et al. 2006) | C994A | **Catalytic cysteine** |  |
| HUWE1 | [Q7Z6Z7](https://www.uniprot.org/uniprot/Q7Z6Z7) | **HECT (4038-4374)** | (Y.-F. Cheng, Tong, and Edge 2016) | C4341A | **Catalytic cysteine** | Atoh1 |
|  |  |  | (de Groot et al. 2014) | C4341A | **Catalytic cysteine** | Dvl |
|  |  |  | (Forget et al. 2014) | C4341A | **Catalytic cysteine** | Atoh1 |
|  |  |  | (Kurokawa et al. 2013) | C4341A | **Catalytic cysteine** | Mcl-1; PP5 |
|  |  |  | (Xiaozhen Wang et al. 2014) | C4341A | **Catalytic cysteine** | BRCA1 |
|  |  |  | (X. Zhao et al. 2008) | C4341S | **Catalytic cysteine** | N-Myc |
| Itch | [Q96J02](https://www.uniprot.org/uniprot/Q96J02) | **HECT (569-903)** | (Angers, Ramjaun, and McPherson 2004) | C830A | **Catalytic cysteine** | Endophilin A1 |
|  |  |  | (Chmura et al. 2017) | C830A | **Catalytic cysteine** | vFLIP |
|  |  |  | (Han et al. 2016) | C830A | **Catalytic cysteine** | VP40 |
|  |  |  | (Theivanthiran et al. 2015) | C830A | **Catalytic cysteine** | Tab1 |
| LNX1 | [Q8TBB1](https://www.uniprot.org/uniprot/Q8TBB1) | **Ring (41-79)** | (Lenihan, Saha, and Young 2017) | C48A (m) | **Zn** | PPFIA1; KLHL11; KIF7; ERC2 |
|  |  |  | (Nie et al. 2002) | C45A (m) | **Zn** | Numb |
|  |  |  | (Wolting et al. 2011) | C45A (m) | **Zn** | Numb |
| MARCH2 | [Q9P0N8](https://www.uniprot.org/uniprot/Q9P0N8) | **RING (56-116)** | (J. Cheng and Guggino 2013) | C64S/C67S | **Zn** | CFTR |
| MARCH5 | [Q9NX47](https://www.uniprot.org/uniprot/Q9NX47) | **Ring (6-75)** | (Z. Chen et al. 2017) | H43W; C65S; C68S | **Zn** | FUNDC1 |
|  |  |  | (Karbowski, Neutzner, and Youle 2007) | H43W; C65S; C68S | **Zn** | Drn1 |
|  |  |  | (Y. Y. Park et al. 2010) | H43W | **Zn** | Mfn1 |
|  |  |  | (Yoo et al. 2015) | H43W | **Zn** | MAVS |
| MARCH6 | [O60337](https://www.uniprot.org/uniprot/O60337) | **Ring (1-62)** | (Zattas et al. 2016) | C9A; C39S | **Zn** |  |
| MARCH8 | [Q5T0T0](https://www.uniprot.org/uniprot/Q5T0T0) | **Ring (72-133)** | (R. Chen et al. 2012) | W114A | **E2** | IL1RAP |
| MARCH9 | [Q86YJ5](https://www.uniprot.org/uniprot/Q86YJ5) | **Ring (102-162)** | (Hör et al. 2009) | W143A | **E2** | FcγRIIb; SLAM |
|  |  |  | (Tan et al. 2019) | W143A | **E2** | HLA-A2 |
| MDM2 | [Q00987](https://www.uniprot.org/uniprot/Q00987) | **Ring (438-479)** | (Bonacci et al. 2017) | C462A | **Zn** | NUB1 |
|  |  |  | (Boyd, Tsai, and Jacks 2000) | C464A | **Zn** | p53 |
|  |  |  | (Brenkman et al. 2008) | C464A | **Zn** | FOXO4 |
|  |  |  | (C. Fan and Wang 2017) | L468A | **E2** | p53 |
|  |  |  | (Fang et al. 2000) | C464A; C461S; C478S; C475G; H452A; H457S; T455A | **Zn** | p53 |
|  |  |  | (Geyer, Yu, and Maki 2000) | C464A | **Zn** | p53 |
|  |  |  | (Gopinathan et al. 2009) | C464A | **Zn** | PPARα |
|  |  |  | (He et al. 2013) | C464A | **Zn** |  |
|  |  |  | (Honda and Yasuda 2000) | C464A; C441A; C449A; C461A; C475A; C478A; C439A | **Zn** | Auto; p53 |
|  |  |  | (Honda, Tanaka, and Yasuda 1997) | C464A | **Zn** | p53 |
|  |  |  | (Inuzuka et al. 2010) | C464A | **Zn** |  |
|  |  |  | (Kannemeier, Liao, and Sun 2007) | C436L; H455S; C459S; C473G | **E2** | p53 |
|  |  |  | (Kawai, Wiederschain, and Yuan 2003) | C464A | **Zn** | p53 |
|  |  |  | (Kubbutat et al. 1999) | C464A | **Zn** | p53 |
|  |  |  | (Linke et al. 2008) | L468A; I440A; P476A | **E2** | Auto; Mdm4 |
|  |  |  |  | R479A | **OTHER** | Auto; Mdm4 |
|  |  |  | (Nomura et al. 2017) | I440E,K | **E2** | p53 |
|  |  |  |  | R479P | **OTHER** | p53 |
|  |  |  | (Pettersson et al. 2009) | C464A | **Zn** | IRF-2 |
|  |  |  | (Poyurovsky et al. 2007) | F490Q | **OTHER** |  |
|  |  |  | (Tian et al. 2017) | C462A | **Zn** | p53 |
|  |  |  | (Uchida et al. 2005) | C438A | **Zn** | pRb |
|  |  |  | (Wawrzynow et al. 2009) | C464A; C478S | **Zn** | p53 |
| MDM4 | [O15151](https://www.uniprot.org/uniprot/O15151) | **Ring (437-478)** | (Egorova and Sheng 2014) | T459A; K478R; S438V/E441Q; T459A/H462T; R453/K478R | **E2** | Auto |
| MEX3C | [Q5U5Q3](https://www.uniprot.org/uniprot/Q5U5Q3) | **Ring (608-648)** | (Kuniyoshi et al. 2014) | C601A (m) | **Zn** | RIG-I |
| MGRN1 | [O60291](https://www.uniprot.org/uniprot/O60291) | **Ring (278-317)** | (Benvegnù, Wahle, and Dotti 2017) | C278A; C281A | **Zn** | APP |
|  |  |  | (Gunn et al. 2013) | C278A; C281A | **Zn** | TSG101 |
|  |  |  | (Jiao et al. 2009) | C278A; C281A | **Zn** | TSG101 |
| MIB2 | [Q96AX9](https://www.uniprot.org/uniprot/Q96AX9) | **Ring 1 (890-925) Ring 2 (969-1002)** | (Ye et al. 2014) | C983S | **Catalytic cysteine*** | MAVS |
| MID1 | [O15344](https://www.uniprot.org/uniprot/O15344) | **Ring (10-60) B-BOX1 (115-165) B-BOX2 (172-212)** | (H. Du et al. 2013) | L146Q | **OTHER** | α4 |
| MKRN1 | [Q9UHC7](https://www.uniprot.org/uniprot/Q9UHC7) | **Ring (281-335)** | (J. H. Kim et al. 2005) | H307E | **Zn** | hTERT |
|  |  |  | (Ko et al. 2010) | H307E | **Zn** | WNVCp |
|  |  |  | (E. Lee et al. 2009) | H307E | **Zn** | p53; p21 |
|  |  |  | (M. S. Lee et al. 2018) | H307E | **Zn** | AMPK |
| MUL1 | [Q969V5](https://www.uniprot.org/uniprot/Q969V5) | **Ring (302-340)** | (Zemirli et al. 2014) | C339A | **Zn** |  |
| MYLIP (IDOL) | [Q8WY64](https://www.uniprot.org/uniprot/Q8WY64) | **Ring (387-422)** | (J. Gao et al. 2017) | C387A | **Zn** | ApoER2 |
|  |  |  | (Hong et al. 2010) | C387A | **Zn** | VLDLR; ApoER2 |
|  |  |  | (Sorrentino et al. 2011) | C387A | **Zn** | LDLR |
|  |  |  | (Zelcer et al. 2009) | C387A | **Zn** | LDLR |
| NEDD4 | [P46934](https://www.uniprot.org/uniprot/P46934) | **HECT (984-1318)** | (Q. Lin et al. 2017) | C867A | **Catalytic cysteine** | SQSTM1 |
|  |  |  | (F. Song et al. 2013) | C967S | **Catalytic cysteine** | THO |
|  |  |  | (Sugeno et al. 2014) | C867A | **Catalytic cysteine** | α-Synuclein |
|  |  |  | (Xinjiang Wang et al. 2008) | C967S | **Catalytic cysteine** | PTEN |
|  |  |  | (Zeng et al. 2014) | C867A | **Catalytic cysteine** | PTEN |
| NEDD4L | [Q96PU5](https://www.uniprot.org/uniprot/Q96PU5) | **HECT (640-974)** | (Albesa et al. 2011) | C801S | **Catalytic cysteine** | hERG1 |
|  |  |  | (Arroyo et al. 2011) | C822S (m) | **Catalytic cysteine** | NCC |
|  |  |  | (Debonneville et al. 2001) | C962A | **Catalytic cysteine** | ENaC |
|  |  |  | (Ding et al. 2013) | C821A | **Catalytic cysteine** | Dvl2 |
|  |  |  | (S. Gao et al. 2009) | C962A | **Catalytic cysteine** | Smad2; Smad3 |
|  |  |  | (Y. H. Kim et al. 2018) | C942A (m) | **Catalytic cysteine** | CRTC3 |
|  |  |  | (Palmada et al. 2004) | C938S | **Catalytic cysteine** | NaPi Iib |
|  |  |  | (D. Xu et al. 2016) | C821A | **Catalytic cysteine** | hOAT1 |
|  |  |  | (R. Zhou, Patel, and Snyder 2007) | C821A | **Catalytic cysteine** | Auto |
|  |  |  |  | C821A | **Catalytic cysteine** | α-,β-, γENaC |
| PJA2 | [O43164](https://www.uniprot.org/uniprot/O43164) | **Ring (634-675)** | (Faust et al. 2017) | C634A/C671A | **Zn** | Tat |
| PRKN | [O60260](https://www.uniprot.org/uniprot/O60260) | **Ring 0 (141-225) Ring 1 (238-293) Ring 2 ( 418-449)** | (Aguileta et al. 2015) | C431F | **Catalytic cysteine*** |  |
|  |  |  | (Ahmed et al. 2011) | G430D; T415N | **OTHER** | Arrestin-3 |
|  |  |  | (Bendikov-Bar et al. 2014) | T240R | **OTHER** | Gcase; PARIS; ARTS |
|  |  |  | (D. Chen et al. 2010) | C431F | **Catalytic cysteine*** | Bcl-2 |
|  |  |  |  | K161N; T240R; P437L | **OTHER** | Bcl-2 |
|  |  |  | (Fiesel et al. 2015) | C431S | **Catalytic cysteine*** |  |
|  |  |  | (Joch et al. 2007) | C431F | **Catalytic cysteine*** | PICK1 |
|  |  |  | (Johnson et al. 2012) | R275W; W453X | **OTHER** | Bax |
|  |  |  | (Juan Liu et al. 2017) | C431A | **Catalytic cysteine*** | HIF-1 |
|  |  |  | (Matteucci et al. 2018) | G430D | **OTHER** | MICU1 |
|  |  |  | (McWilliams et al. 2018) | C431S | **Catalytic cysteine*** |  |
|  |  |  | (Moore et al. 2008) | T240R | **OTHER** | Hsp70 |
|  |  |  | (Riley et al. 2013) | C431S, A | **Catalytic cysteine*** |  |
|  |  |  | (Sarraf et al. 2013) | C431F | **Catalytic cysteine*** |  |
|  |  |  | (P. Song et al. 2016) | C431S | **Catalytic cysteine*** | Rab7 |
|  |  |  | (Y. Wang et al. 2018) | K151E | **OTHER** | RIPK1 |
|  |  |  | (Wauer et al. 2015) | K151E | **OTHER** |  |
|  |  |  | (Xin et al. 2018) | C431S | **Catalytic cysteine*** | TRAF3 |
| RAD18 | [Q9NS91](https://www.uniprot.org/uniprot/Q9NS91) | **Ring (25-64)** | (J. Huang et al. 2009) | C28F | **Zn** |  |
|  |  |  | (Masuda et al. 2012) | I50A/R51A | **E2** |  |
|  |  |  | (Tateishi et al. 2000) | C28F | **Zn** |  |
|  |  |  | (Williams et al. 2011) | C28F | **Zn** | FANCD2 |
| RAG1 | [P15918](https://www.uniprot.org/uniprot/P15918) | **(Ring 293-332)** | (Jones and Gellert 2003) | C328Y | **Zn** | Auto |
| RC3H2 | [Q9HBD1](https://www.uniprot.org/uniprot/Q9HBD1) | **Ring (14-54)** | (Maruyama et al. 2014) | C33S | **Zn** | ASK1 |
| RFFL | [Q8WZ73](https://www.uniprot.org/uniprot/Q8WZ73) | **Ring (316-351)** | (Sakai et al. 2019) | C316A/C319A; H333A | **Zn** | Rab11 effectors |
| RFWD3 | [Q6PCD5](https://www.uniprot.org/uniprot/Q6PCD5) | **Ring (287-331)** | (Feeney et al. 2017) | C315A | **Zn** |  |
| Ring1 | [Q06587](https://www.uniprot.org/uniprot/Q06587) | **Ring (48-88)** | (Shen et al. 2018) | I50A | **E2** | p53 |
| RLIM | [Q9NVW2](https://www.uniprot.org/uniprot/Q9NVW2) | **Ring (570-611)** | (R. Gao, Wang, et al. 2016) | C596A | **Zn** | c-Myc |
| RNF125 | [Q96EQ8](https://www.uniprot.org/uniprot/Q96EQ8) | **Ring (37-76)** | (Jia et al. 2017) | C72A/C75A | **Zn** | TRIM14 |
|  |  |  | (L. Yang et al. 2015) | C72A/C75A | **Zn** | p53 |
| RNF126 | [Q9BV68](https://www.uniprot.org/uniprot/Q9BV68) | **Ring (229-270)** | (Benini et al. 2017) | C229A/C232A | **Zn** | Frataxin |
| RNF138 | [Q8WVD3](https://www.uniprot.org/uniprot/Q8WVD3) | **Ring (18-58)** | (W. Kim et al. 2018) | C18A/C54A | **Zn** | rpS3 |
| RNF144A | [P50876](https://www.uniprot.org/uniprot/P50876) | **Ring 1 (20-70) IBR (91-156) Ring 2 atypical (185-214)** | (Ye Zhang et al. 2017) | C20A/C23A | **Zn** | PARP1 |
|  |  |  | (Ho et al. 2014) | C20A/C23A | **Zn** | DNA-PKcs |
| RNF145 | [Q96MT1](https://www.uniprot.org/uniprot/Q96MT1) | **Ring (537-575)** | (Jiang et al. 2018) | C537A | **Zn** | HMGCR |
|  |  |  | (Menzies et al. 2018) | C552A/H554A | **Zn** | HMGCR |
| RNF146 | [Q9NTX7](https://www.uniprot.org/uniprot/Q9NTX7) | **Ring (37-75)** | (Callow et al. 2011) | H53A | **Zn** | Axin; Tankyrase |
| RNF152 | [Q8N8N0](https://www.uniprot.org/uniprot/Q8N8N0) | **Ring (12-55)** | (Deng et al. 2015) | 4C-->S | **Zn** | RagA GTPase |
| RNF167 | [Q9H6Y7](https://www.uniprot.org/uniprot/Q9H6Y7) | **Ring (230-272)** | (Deshar et al. 2016) | C233S | **Zn** | Arl8B |
| RNF168 | [Q8IYW5](https://www.uniprot.org/uniprot/Q8IYW5) | **Ring (16-55)** | (Pinato et al. 2009) | C16S/C19S | **Zn** | H2A; H2AX |
| RNF185 | [Q96GF1](https://www.uniprot.org/uniprot/Q96GF1) | **Ring (39-80)** | (El Khouri et al. 2013) | C39A/C42A | **Zn** | CFTR |
| RNF2 | [Q99496](https://www.uniprot.org/uniprot/Q99496) | **Ring (51-91)** | (S. Liu et al. 2018) | I53A | **E2** | H2A |
|  |  |  | (Xia et al. 2014) | H69Y | **Zn** | AMBRA1 |
| RNF220 | [Q6PDX6](https://www.uniprot.org/uniprot/Q6PDX6) | **Ring (513-553)** | (Ma et al. 2014) | W539R | **E2** | Sin3B |
| RNF25 | [Q96BH1](https://www.uniprot.org/uniprot/Q96BH1) | **Ring (134-203)** | (R. Gao, Ma, et al. 2016) | C135A/C138A | **Zn** |  |
| RNF26 | [Q9BY78](https://www.uniprot.org/uniprot/Q9BY78) | **Ring (378-425)** | (Qin et al. 2014) | C395S; C399S; C401S | **Zn** | STING |
| RNF31 | [Q96EP0](https://www.uniprot.org/uniprot/Q96EP0) | **Ring 1 (699-749)IBR (779-841) Ring 2 (871-901)** | (Smit et al. 2012) | C871A/C874A; C890A/C893A; C885A/H887A; C898A/C901A; C719A; C885A/H887A; C885S | **Zn** |  |
|  |  |  | (Zhu et al. 2018) | C871A/C874A; C890A/C893A; C885A/H887A; C898A/C901A; C719A; C885A/H887A; C885S | **Zn** | FOXP3 |
| RNF34 | [Q969K3](https://www.uniprot.org/uniprot/Q969K3) | **Ring (325-360)** | (H. Jin et al. 2014) | H342A | **Zn** | GABA_A_Rs |
|  |  |  | (Wei et al. 2018) | C656A (d) | **Zn** | PGC-1 |
|  |  |  | (R. Zhang et al. 2014) | H342A | **Zn** | NOD-1 |
| RNF4 | [P78317](https://www.uniprot.org/uniprot/P78317) | **Ring (132-177)** | (Liew et al. 2010) | M149A; D141A; V161A; V134E; S155E R181A; Y193A (m) | **OTHER** |  |
| RNF40 | [O75150](https://www.uniprot.org/uniprot/O75150) | **Ring (948-987)** | (Foglizzo, Middleton, and Day 2016) | Y999A | **OTHER** |  |
| RNF43 | [Q68DV7](https://www.uniprot.org/uniprot/Q68DV7) | **Ring (272-313)** | (Loregger et al. 2015) | H292R | **Zn** | TCF4 |
| RNF8 | [O76064](https://www.uniprot.org/uniprot/O76064) | **Ring (403-441)** | (Lu et al. 2012) | C403S | **Zn** | Nsb1 |
|  |  |  | (Mailand et al. 2007) | C403S | **Zn** | H2A; H2AX |
|  |  |  | (Mallette et al. 2012) | I405A | **E2** | JMJD2A |
|  |  |  | (Rai et al. 2011) | C406S | **Zn** | TPP1 |
|  |  |  | (Tripathi and Smith 2017) | C403S | **Zn** | TNKS1 |
| SHPRH | [Q149N8](https://www.uniprot.org/uniprot/Q149N8) | **Ring (1432-1479)** | (Motegi et al. 2006) | C1432A | **Zn** | PCNA |
| SIAH1 | [Q8IUQ4](https://www.uniprot.org/uniprot/Q8IUQ4) | **Ring (41-76)** | (Grishina et al. 2012) | C44S | **Zn** | CBP/p300 |
|  |  |  | (Ji et al. 2017) | C41S/C44S (m) | **Zn** | Axin 1 |
|  |  |  | (Se-yong Kim et al. 2009) | C41S/C44S (m) | **Zn** | HIPK2 |
|  |  |  | (S. Lee et al. 2015) | C44S | **Zn** | p34 |
|  |  |  | (M. Liu et al. 2012) | C75S | **Zn** | ELL2 |
|  |  |  | (Pietschmann et al. 2012) | C72S | **Zn** | PML-RARα |
|  |  |  | (Y. Zhou et al. 2008) | C41S/C44S (m) | **Zn** | TRB3 |
| SIAH2 | [O43255](https://www.uniprot.org/uniprot/O43255) | **Ring (80-115)** | (Habelhah et al. 2002) | H99A/C102A (m) | **Zn** | TRAF2 |
| SMURF1 | [Q9HCE7](https://www.uniprot.org/uniprot/Q9HCE7) | **HECT (420-757)** | (Fei et al. 2013) | C699A (m) | **Catalytic cysteine** | Axin |
|  |  |  | (Shan Li et al. 2010) | C699A (m) | **Catalytic cysteine** | TRAF4 |
|  |  |  | (Tajima et al. 2003) | I612A/L614A (m) | **OTHER** | Smad7 |
|  |  |  | (H.-R. Wang et al. 2006) | C699A (m) | **Catalytic cysteine** |  |
|  |  |  | (Xiangchun Wang et al. 2013) | C710A | **Catalytic cysteine** | TRAF4 |
|  |  |  | (M. Zhao et al. 2003) | C710A | **Catalytic cysteine** | Smad1; Cbfa1 |
| SMURF2 | [Q9HAU4](https://www.uniprot.org/uniprot/Q9HAU4) | **HECT (414-748)** | (Borroni et al. 2018) | C716G | **Catalytic cysteine** | Lamin A |
|  |  |  | (J. X. Du et al. 2011) | C716A | **Catalytic cysteine** | KLF5 |
|  |  |  | (Jeong et al. 2014) | C716G | **Catalytic cysteine** | YY1 |
|  |  |  | (C. Jin et al. 2009) | C716G | **Catalytic cysteine** |  |
|  |  |  | (Sewoon Kim and Jho 2010) | C716G | **Catalytic cysteine** | Axin |
|  |  |  | (Pan et al. 2014) | C716A | **Catalytic cysteine** | MAVS |
|  |  |  | (Shukla et al. 2014) | C716A | **Catalytic cysteine** | KRAS |
| STUB1 | [Q9UNE7](https://www.uniprot.org/uniprot/Q9UNE7) | **U-BOX (226-300)** | (M. Fan, Park, and Nephew 2005) | H260Q | **E2** | Erα |
|  |  |  | (J.-H. Kim et al. 2017) | H260Q | **E2** | PPARγ |
|  |  |  | (X. Li et al. 2018) | H260Q | **E2** | IRS4 |
|  |  |  |  | P269A | **E2** | IRS4 |
|  |  |  | (Seo et al. 2018) | H260Q | **E2** | SNPH |
|  |  |  | (Shimamoto et al. 2013) | H260Q | **E2** |  |
|  |  |  |  | P269A | **E2** |  |
| SYVN1 | [Q86TM6](https://www.uniprot.org/uniprot/Q86TM6) | **Ring (291-330)** | (Tanabe et al. 2012) | C307A | **Zn** | RER1 |
| TRAF6 | [Q9Y4K3](https://www.uniprot.org/uniprot/Q9Y4K3) | **Ring (70-109)** | (Y. B. Choi and Harhaj 2014) | C70A | **Zn** | Mcl-1 |
|  |  |  | (Funakoshi-Tago et al. 2009) | C70A | **Zn** |  |
|  |  |  | (Ning et al. 2008) | C70A | **Zn** | IRF7 |
|  |  |  | (W. L. Yang et al. 2009) | C70A | **Zn** | Akt |
|  |  |  | (Jiazhen Zhang et al. 2017) | C70A | **Zn** |  |
|  |  |  |  | L74H | **E2** |  |
| TRIM11 | [Q96F44](https://www.uniprot.org/uniprot/Q96F44) | **Ring (16-57) B-BOX (87-128)** | (L. Chen et al. 2018) | C16A/C19A | **Zn** |  |
|  |  |  | (T. Liu et al. 2016) | C53A7 C56A | **Zn** | AIM2 |
| TRIM13 | [O60858](https://www.uniprot.org/uniprot/O60858) | **Ring (10-58) B-BOX (89-131)** | (B. Huang et al. 2018) | C10A/C13A | **Zn** | Nur77 |
| TRIM17 | [Q9Y577](https://www.uniprot.org/uniprot/Q9Y577) | **Ring (16-66) B-BOX (94-135)** | (Lassot et al. 2010) | C16A | **Zn** |  |
| TRIM21 | [P19474](https://www.uniprot.org/uniprot/P19474) | **Ring (16-55) B-BOX (92-123)** | (Wada and Kamitani 2006) | C16A | **Zn** | p62 |
| TRIM22 | [Q8IYM9](https://www.uniprot.org/uniprot/Q8IYM9) | **Ring (15-60) B-BOX (92-133)** | (Duan et al. 2008) | C15A | **Zn** | Auto |
| TRIM23 | [P36406](https://www.uniprot.org/uniprot/P36406) | **Ring (31-76) B-BOX (122-168)** | (Arimoto et al. 2010) | C34A | **Zn** | NEMO |
|  |  |  | (Sparrer et al. 2017) | C34A | **Zn** |  |
| TRIM25 | [Q14258](https://www.uniprot.org/uniprot/Q14258) | **Ring (13-54)** | (J. M. Lee et al. 2018) | C50S/C53S | **Zn** | PPARγ |
| TRIM26 | [Q12899](https://www.uniprot.org/uniprot/Q12899) | **Ring (16-57) B-BOX (97-138)** | (Ran et al. 2016) | C31S | **Zn** |  |
| TRIM27 | [P14373](https://www.uniprot.org/uniprot/P14373) | **Ring (16-57) B-BOX (96-127)** | (Zaman et al. 2013) | C96A/C99A/H107A/D110A | **Zn** | USP7 |
|  |  |  | (Zurek et al. 2012) | C16A/C31A | **Zn** | NOD2 |
| TRIM3 | [O75382](https://www.uniprot.org/uniprot/O75382) | **Ring (22-63) B-BOX (110-151)** | (Hung et al. 2010) | C22A/C25A | **Zn** | GKAP/SAPAP1 |
|  |  |  | (Raheja et al. 2014) | C22A/C25A | **Zn** | p21 |
|  |  |  |  | I24A; L26A; D27A; V61A; R63A | **E2** | p21 |
| TRIM31 | [Q9BZY9](https://www.uniprot.org/uniprot/Q9BZY9) | **Ring (16-57) B-BOX (90-131)** | (B. Liu et al. 2017) | C53A/C56A | **Zn** | MAVS |
|  |  |  | (H. Song et al. 2016) | C16A/C36A | **Zn** | NLRP3 |
| TRIM32 | [Q13049](https://www.uniprot.org/uniprot/Q13049) | **Ring (20-65) B-BOX (103-133)** | (Fu et al. 2015) | C39S | **Zn** | PB1 |
|  |  |  | (Koliopoulos et al. 2016) | E16R | **OTHER** |  |
|  |  |  | (Ryu et al. 2011) | C23A | **Zn** | XIAP |
|  |  |  | (Jing Zhang et al. 2012) | C39S | **Zn** | STING |
| TRIM33 | [Q9UPN9](https://www.uniprot.org/uniprot/Q9UPN9) | **Ring (125-154) B-BOX1 (212-259) B-BOX2 (271-312)** | (Xue et al. 2015) | C125A/C128A | **Zn** | β-catenin |
| TRIM37 | [O94972](https://www.uniprot.org/uniprot/O94972) | **Ring (15-55) B-BOX (90-132)** | (Bhatnagar et al. 2014) | C18R | **Zn** | H2A |
|  |  |  | (Kallijärvi et al. 2005) | C35S/C36S | **Zn** |  |
|  |  |  | (W. Wang et al. 2017) | C35S/C36S; C18R | **Zn** | PEX5 |
| TRIM4 | [Q9C037](https://www.uniprot.org/uniprot/Q9C037) | **Ring (12-53) B-BOX (82-123)** | (J. Yan et al. 2014) | C27S | **Zn** | RIG-1 |
| TRIM45 | [Q9H8W5](https://www.uniprot.org/uniprot/Q9H8W5) | **Ring (29-98) B-BOX1 (130-176) B-BOX2 (186-227)** | (Jindong Zhang et al. 2017) | C29A | **Zn** | p53 |
| TRIM49 | [P0CI25](https://www.uniprot.org/uniprot/P0CI25) | **Ring (15-56) B-BOX (88-129)** | (Guimarães and Gomes 2018) | C35S | **Zn** |  |
| TRIM5 | [Q9C035](https://www.uniprot.org/uniprot/Q9C035) | **Ring (15-59) B-BOX (90-132)** | (Lienlaf et al. 2011) | R60A | **E2** | Auto |
|  |  |  | (Yamauchi et al. 2008) | C15A | **Zn** |  |
|  |  |  | (Yudina et al. 2015) | Y63E; I77R | **OTHER** |  |
| TRIM50 | [Q86XT4](https://www.uniprot.org/uniprot/Q86XT4) | **Ring (16-57) B-BOX (84-125)** | (Fusco et al. 2012) | C52X (m) | **Zn** |  |
| TRIM6 | [Q9C030](https://www.uniprot.org/uniprot/Q9C030) | **Ring (15-60) B-BOX (92-133)** | (Bharaj et al. 2017) | C15A | **Zn** | VP35 |
| TRIM62 | [Q9BVG3](https://www.uniprot.org/uniprot/Q9BVG3) | **Ring (11-54) B-BOX (88-128)** | (Zhifang Cao et al. 2015) | C11A/C14A | **Zn** | CARD9 |
|  |  |  | (F. Huang et al. 2013) | C11A | **Zn** |  |
| TRIM65 | [Q6PJ69](https://www.uniprot.org/uniprot/Q6PJ69) | **Ring (12-51) B-BOX (90-137)** | (Shitao Li et al. 2014) | C12A/C15A | **Zn** | TNRC6 |
| TRIM7 | [Q9C029](https://www.uniprot.org/uniprot/Q9C029) | **Ring (29-82) B-BOX (125-166)** | (Chakraborty et al. 2015) | C29A/C32A | **Zn** | RACO-1 |
|  |  |  |  | W57A | **E2** | RACO-1 |
| TRIM71 | [Q2Q1W2](https://www.uniprot.org/uniprot/Q2Q1W2) | **Ring (12-95) B-BOX 1 (194-241) B-BOX2 (273-314)** | (Yin et al. 2016) | C12A/C15A | **Zn** | Lin28B |
| TRIM8 | [Q9BZR9](https://www.uniprot.org/uniprot/Q9BZR9) | **Ring (15-56) B-BOX1 (92-132) B-BOX2 (140-182)** | (F.-J. Yan et al. 2017) | C15A/C18A | **Zn** | TAK1 |
| TRIP12 | [Q14669](https://www.uniprot.org/uniprot/Q14669) | **HECT (1885-1992)** | (Hanoun et al. 2014) | C1959A | **Catalytic cysteine** | PTF1a |
|  |  |  | (Y. Park, Yoon, and Yoon 2009) | C1972S | **Catalytic cysteine** |  |
| UBE3A | [Q05086](https://www.uniprot.org/uniprot/Q05086) | **HECT (776-875)** | (Chhabra et al. 2017) | C843A | **Catalytic cysteine** | G-CSFR |
|  |  |  | (Harlalka et al. 2013) | C820A | **Catalytic cysteine** |  |
|  |  |  | (Kumar, Talis, and Howley 1999) | C833A | **Catalytic cysteine** | HHR23 |
|  |  |  | (S. Y. Lee et al. 2014) | C941S (d) | **Catalytic cysteine** | Rpn10 |
|  |  |  | (Mortensen et al. 2015) | C820A | **Catalytic cysteine** |  |
|  |  |  | (Munakata et al. 2007) | C840A | **Catalytic cysteine** | pRb |
|  |  |  | (Pal et al. 2013) | C843A | **Catalytic cysteine** | C/EBPα |
|  |  |  | (Y. Yang et al. 2007) | C833A | **Catalytic cysteine** | TH1 |
| UBE3C | [Q15386](https://www.uniprot.org/uniprot/Q15386) | **HECT (744-1083)** | (Chu et al. 2013) | C1051A | **Catalytic cysteine** |  |
|  |  |  | (Y. Yu and Hayward 2010) | C1051A | **Catalytic cysteine** | IRF3; IRF7 |
| UBE4B | [O95155](https://www.uniprot.org/uniprot/O95155) | **U-BOX (1227-1300)** | (Okumura et al. 2004) | P1140A (m) | **OTHER** | FEZ1 |
| UBR1 | [Q8IWV7](https://www.uniprot.org/uniprot/Q8IWV7) | **Ring (1098-1201)** | (Sasaki et al. 2006) | C1098S | **Zn** | c-FOS |
| UBR5 | [O95071](https://www.uniprot.org/uniprot/O95071) | **HECT (2462-2799)** | (T. Zhang et al. 2014) | C2768A | **Catalytic cysteine** | ATMIN |
| UHRF1 | [Q96T88](https://www.uniprot.org/uniprot/Q96T88) | **Ring (724-763)** | (Nishiyama et al. 2013) | C713A/C715A/C716A (m) | **Zn** | H3 |
|  |  |  | (H. Zhang et al. 2016) | H754A | **Zn** | RIF-1 |
| WWP1 | [Q9H0M0](https://www.uniprot.org/uniprot/Q9H0M0) | **HECT (588-922)** | (Han et al. 2017) | C890A | **Catalytic cysteine** | VP40 |
|  |  |  | (Heidecker et al. 2007) | C890S | **Catalytic cysteine** | Gag |
|  |  |  | (Laine and Ronai 2007) | C883A | **Catalytic cysteine** | p53 |
|  |  |  | (L. Lin et al. 2016) | C886S (m) | **Catalytic cysteine** | Htt (160Q) |
|  |  |  | (Zaarour et al. 2012) | C890A | **Catalytic cysteine** | Ezrin |
|  |  |  | (Z. Zhou, Liu, and Chen 2012) | C890A | **Catalytic cysteine** |  |
| WWP2 | [O00308](https://www.uniprot.org/uniprot/O00308) | **HECT (536-870)** | (Jung et al. 2014) | C838A | **Catalytic cysteine** | Notch3 |
|  |  |  | (Luo et al. 2014) | C838A | **Catalytic cysteine** | SRG3 |
|  |  |  | (Nakamura et al. 2011) | C838A | **Catalytic cysteine** | Sox9 |
|  |  |  | (H. M. Xu et al. 2004) | C838A | **Catalytic cysteine** | oct-04 |
| XIAP | [P98170](https://www.uniprot.org/uniprot/P98170) | **Ring (450-485)** | (Zipeng Cao et al. 2013) | H467A | **Zn** | Cyclin D1 |
|  |  |  | (Jinyi Liu et al. 2012) | H467A | **Zn** |  |
|  |  |  | (Nakatani et al. 2013) | F495A; F495L; V461E | **OTHER** |  |
|  |  |  | (Q. Yang 2004) | H467A | **Zn** | Auto |
| ZNRF1 | [Q8ND25](https://www.uniprot.org/uniprot/Q8ND25) | **Ring (184-225)** | (Toshiyuki and Milbrandt 2003) | C184A | **Zn** |  |
| ZNRF2 | [Q8NHG8](https://www.uniprot.org/uniprot/Q8NHG8) | **Ring (199-240)** | (Toshiyuki and Milbrandt 2003) | C199A | **Zn** |  |
| ZNRF4 | [Q8WWF5](https://www.uniprot.org/uniprot/Q8WWF5) | **Ring (309-352)** | (Bist et al. 2017) | H329W/H332W | **Zn** | RIP2 |

Aguileta, Miguel A, Jelena Korac, Thomas M Durcan, Michael Haber, Kalle Gehring, Suzanne Elsasser, Oliver Waidmann, Edward A Fon, and Koraljka Husnjak. 2015. “The E3 Ubiquitin Ligase Parkin Is Recruited to the 26 S Proteasome via the Proteasomal Ubiquitin Receptor Rpn13 *” 290 (12): 7492–7505. https://doi.org/10.1074/jbc.M114.614925.

Ahmed, M Rafiuddin, Xuanzhi Zhan, Xiufeng Song, Seunghyi Kook, Vsevolod V Gurevich, and Eugenia V Gurevich. 2011. “Ubiquitin Ligase Parkin Promotes Mdm2-Arrestin Interaction but Inhibits Arrestin Ubiquitination.” *Biochemistry* 50 (18): 3749–63. https://doi.org/10.1021/bi200175q.

Albert, Thomas K, Hiroyuki Hanzawa, Yvonne I A Legtenberg, Marjolein J de Ruwe, Fiona A J van den Heuvel, Martine A Collart, Rolf Boelens, and H Th Marc Timmers. 2002. “Identification of a Ubiquitin-Protein Ligase Subunit within the CCR4-NOT Transcription Repressor Complex.” *The EMBO Journal* 21 (3): 355–64. https://doi.org/10.1093/emboj/21.3.355.

Albesa, Maxime, Liliana Sintra Grilo, Bruno Gavillet, and Hugues Abriel. 2011. “Nedd4-2-Dependent Ubiquitylation and Regulation of the Cardiac Potassium Channel HERG1.” *Journal of Molecular and Cellular Cardiology* 51 (1): 90–98. https://doi.org/10.1016/j.yjmcc.2011.03.015.

Alchanati, Iris, Carmit Teicher, Galit Cohen, Vivian Shemesh, Haim M. Barr, Philippe Nakache, Danny Ben-Avraham, et al. 2009. “The E3 Ubiquitin-Ligase Bmi1/Ring1A Controls the Proteasomal Degradation of Top2α Cleavage Complex - A Potentially New Drug Target.” *PLoS ONE*. https://doi.org/10.1371/journal.pone.0008104.

Angers, Annie, Antoine R Ramjaun, and Peter S McPherson. 2004. “The HECT Domain Ligase Itch Ubiquitinates Endophilin and Localizes to the Trans-Golgi Network and Endosomal System.” *The Journal of Biological Chemistry* 279 (12): 11471–79. https://doi.org/10.1074/jbc.M309934200.

Anglesio, Michael S, Valentina Evdokimova, Nataliya Melnyk, Liyong Zhang, Conrad V Fernandez, Paul E Grundy, Stephen Leach, et al. 2004. “Differential Expression of a Novel Ankyrin Containing E3 Ubiquitin-Protein Ligase, Hace1, in Sporadic Wilms’ Tumor versus Normal Kidney.” *Human Molecular Genetics* 13 (18): 2061–74. https://doi.org/10.1093/hmg/ddh215.

Ardley, H C, N G Tan, S A Rose, A F Markham, and P A Robinson. 2001. “Features of the Parkin/Ariadne-like Ubiquitin Ligase, HHARI, That Regulate Its Interaction with the Ubiquitin-Conjugating Enzyme, Ubch7.” *The Journal of Biological Chemistry* 276 (22): 19640–47. https://doi.org/10.1074/jbc.M011028200.

Arimoto, Kei-ichiro, Kenji Funami, Yasushi Saeki, Keiji Tanaka, Katsuya Okawa, Osamu Takeuchi, Shizuo Akira, Yoshiki Murakami, and Kunitada Shimotohno. 2010. “Polyubiquitin Conjugation to NEMO by Triparite Motif Protein 23 (TRIM23) Is Critical in Antiviral Defense.” *Proceedings of the National Academy of Sciences of the United States of America* 107 (36): 15856–61. https://doi.org/10.1073/pnas.1004621107.

Arroyo, Juan Pablo, Dagmara Lagnaz, Caroline Ronzaud, Norma Vázquez, Benjamin S Ko, Lauren Moddes, Dorothée Ruffieux-Daidié, et al. 2011. “Nedd4-2 Modulates Renal Na+-Cl- Cotransporter via the Aldosterone-SGK1-Nedd4-2 Pathway.” *Journal of the American Society of Nephrology : JASN* 22 (9): 1707–19. https://doi.org/10.1681/ASN.2011020132.

Bachmaier, Kurt, Sophie Toya, Xiaopei Gao, Thomas Triantafillou, Sean Garrean, Gye Young Park, Randall S Frey, et al. 2007. “E3 Ubiquitin Ligase Cblb Regulates the Acute Inflammatory Response Underlying Lung Injury.” *Nature Medicine* 13 (8): 920–26. https://doi.org/10.1038/nm1607.

Bendikov-Bar, Inna, Debora Rapaport, Sarit Larisch, and Mia Horowitz. 2014. “Parkin-Mediated Ubiquitination of Mutant Glucocerebrosidase Leads to Competition with Its Substrates PARIS and ARTS.” *Orphanet Journal of Rare Diseases* 9 (June): 86. https://doi.org/10.1186/1750-1172-9-86.

Benini, Monica, Silvia Fortuni, Ivano Condò, Giulia Alfedi, Florence Malisan, Nicola Toschi, Dario Serio, et al. 2017. “E3 Ligase RNF126 Directly Ubiquitinates Frataxin, Promoting Its Degradation: Identification of a Potential Therapeutic Target for Friedreich Ataxia.” *Cell Reports* 18 (8): 2007–17. https://doi.org/10.1016/j.celrep.2017.01.079.

Benvegnù, Stefano, Tina Wahle, and Carlos G Dotti. 2017. “E3 Ligase Mahogunin (MGRN1) Influences Amyloid Precursor Protein Maturation and Secretion.” *Oncotarget* 8 (52): 89439–50. https://doi.org/10.18632/oncotarget.20143.

Bharaj, Preeti, Colm Atkins, Priya Luthra, Maria Isabel Giraldo, Brian E Dawes, Lisa Miorin, Jeffrey R Johnson, et al. 2017. “The Host E3-Ubiquitin Ligase TRIM6 Ubiquitinates the Ebola Virus VP35 Protein and Promotes Virus Replication.” *Journal of Virology* 91 (18). https://doi.org/10.1128/JVI.00833-17.

Bhatnagar, Sanchita, Claude Gazin, Lynn Chamberlain, Jianhong Ou, Xiaochun Zhu, Jogender S Tushir, Ching-Man Virbasius, et al. 2014. “TRIM37 Is a New Histone H2A Ubiquitin Ligase and Breast Cancer Oncoprotein.” *Nature* 516 (7529): 116–20. https://doi.org/10.1038/nature13955.

Bist, Pradeep, Wan Shoo Cheong, Aylwin Ng, Neha Dikshit, Bae-Hoon Kim, Niyas Kudukkil Pulloor, Hanif Javanmard Khameneh, et al. 2017. “E3 Ubiquitin Ligase ZNRF4 Negatively Regulates NOD2 Signalling and Induces Tolerance to MDP.” *Nature Communications* 8: 15865. https://doi.org/10.1038/ncomms15865.

Blankenship, John W, Eugene Varfolomeev, Tatiana Goncharov, Anna V Fedorova, Donald S Kirkpatrick, Anita Izrael-Tomasevic, Lilian Phu, et al. 2009. “Ubiquitin Binding Modulates IAP Antagonist-Stimulated Proteasomal Degradation of c-IAP1 and c-IAP2.” *The Biochemical Journal* 417 (1): 149–60. https://doi.org/10.1042/BJ20081885.

Bonacci, Thomas, Stéphane Audebert, Luc Camoin, Emilie Baudelet, Juan-Lucio Iovanna, and Philippe Soubeyran. 2017. “Regulation of NUB1 Activity through Non-Proteolytic Mdm2-Mediated Ubiquitination.” *PloS One* 12 (1): e0169988. https://doi.org/10.1371/journal.pone.0169988.

Borroni, Aurora Paola, Andrea Emanuelli, Pooja Anil Shah, Nataša Ilić, Liat Apel-Sarid, Biagio Paolini, Dhanoop Manikoth Ayyathan, Praveen Koganti, Gal Levy-Cohen, and Michael Blank. 2018. “Smurf2 Regulates Stability and the Autophagic-Lysosomal Turnover of Lamin A and Its Disease-Associated Form Progerin.” *Aging Cell* 17 (2). https://doi.org/10.1111/acel.12732.

Boyd, S D, K Y Tsai, and T Jacks. 2000. “An Intact HDM2 RING-Finger Domain Is Required for Nuclear Exclusion of P53.” *Nature Cell Biology* 2 (9): 563–68. https://doi.org/10.1038/35023500.

Brenkman, Arjan B, Peter L J de Keizer, Niels J F van den Broek, A G Jochemsen, and Boudewijn M Th Burgering. 2008. “Mdm2 Induces Mono-Ubiquitination of FOXO4.” *PloS One* 3 (7): e2819. https://doi.org/10.1371/journal.pone.0002819.

Bulut, Gamze B, Rita Sulahian, Huiyu Yao, and Lily Jun-shen Huang. 2013. “Cbl Ubiquitination of P85 Is Essential for Epo-Induced EpoR Endocytosis.” *Blood* 122 (24): 3964–72. https://doi.org/10.1182/blood-2013-05-506212.

Callow, Marinella G, Hoanh Tran, Lilian Phu, Ted Lau, James Lee, Wendy N Sandoval, Peter S Liu, et al. 2011. “Ubiquitin Ligase RNF146 Regulates Tankyrase and Axin to Promote Wnt Signaling.” *PloS One* 6 (7): e22595. https://doi.org/10.1371/journal.pone.0022595.

Cao, Zhifang, Kara L Conway, Robert J Heath, Jason S Rush, Elizaveta S Leshchiner, Zaida G Ramirez-Ortiz, Natalia B Nedelsky, et al. 2015. “Ubiquitin Ligase TRIM62 Regulates CARD9-Mediated Anti-Fungal Immunity and Intestinal Inflammation.” *Immunity* 43 (4): 715–26. https://doi.org/10.1016/j.immuni.2015.10.005.

Cao, Zipeng, Ruowen Zhang, Jingxia Li, Haishan Huang, Dongyun Zhang, Jingjie Zhang, Jimin Gao, Jingyuan Chen, and Chuanshu Huang. 2013. “X-Linked Inhibitor of Apoptosis Protein (XIAP) Regulation of Cyclin D1 Protein Expression and Cancer Cell Anchorage-Independent Growth via Its E3 Ligase-Mediated Protein Phosphatase 2A/c-Jun Axis.” *The Journal of Biological Chemistry* 288 (28): 20238–47. https://doi.org/10.1074/jbc.M112.448365.

Chakraborty, Atanu, Markus E Diefenbacher, Anastasia Mylona, Olivier Kassel, and Axel Behrens. 2015. “The E3 Ubiquitin Ligase Trim7 Mediates C-Jun/AP-1 Activation by Ras Signalling.” *Nature Communications* 6 (April): 6782. https://doi.org/10.1038/ncomms7782.

Chan, Nickie C., Willem Den Besten, Michael J. Sweredoski, Sonja Hess, Raymond J. Deshaies, and David C. Chan. 2014. “Degradation of the Deubiquitinating Enzyme USP33 Is Mediated by P97 and the Ubiquitin Ligase HERC2.” *Journal of Biological Chemistry* 289 (28): 19789 –19798. https://doi.org/10.1074/jbc.M114.569392.

Chen, Dong, Feng Gao, Bin Li, Hongfeng Wang, Yuxia Xu, Cuiqing Zhu, and Guanghui Wang. 2010. “Parkin Mono-Ubiquitinates Bcl-2 and Regulates Autophagy.” *The Journal of Biological Chemistry* 285 (49): 38214–23. https://doi.org/10.1074/jbc.M110.101469.

Chen, Liang, Guixin Zhu, Eleanor M Johns, and Xiaolu Yang. 2018. “TRIM11 Activates the Proteasome and Promotes Overall Protein Degradation by Regulating USP14.” *Nature Communications* 9 (1): 1223. https://doi.org/10.1038/s41467-018-03499-z.

Chen, Rui, Mi Li, Yu Zhang, Qian Zhou, and Hong-Bing Shu. 2012. “The E3 Ubiquitin Ligase MARCH8 Negatively Regulates IL-1β-Induced NF-ΚB Activation by Targeting the IL1RAP Coreceptor for Ubiquitination and Degradation.” *Proceedings of the National Academy of Sciences of the United States of America* 109 (35): 14128–33. https://doi.org/10.1073/pnas.1205246109.

Chen, Ziheng, Lei Liu, Qi Cheng, Yanjun Li, Hao Wu, Weilin Zhang, Yueying Wang, et al. 2017. “Mitochondrial E3 Ligase MARCH5 Regulates FUNDC1 to Fine-Tune Hypoxic Mitophagy.” *EMBO Reports* 18 (3): 495–509. https://doi.org/10.15252/embr.201643309.

Cheng, Jie, and William Guggino. 2013. “Ubiquitination and Degradation of CFTR by the E3 Ubiquitin Ligase MARCH2 through Its Association with Adaptor Proteins CAL and STX6.” *PloS One* 8 (6): e68001. https://doi.org/10.1371/journal.pone.0068001.

Cheng, Yen-Fu, Mingjie Tong, and Albert S B Edge. 2016. “Destabilization of Atoh1 by E3 Ubiquitin Ligase Huwe1 and Casein Kinase 1 Is Essential for Normal Sensory Hair Cell Development.” *The Journal of Biological Chemistry* 291 (40): 21096–109. https://doi.org/10.1074/jbc.M116.722124.

Chhabra, Stuti, Yogesh Kumar, Gatha Thacker, Isha Kapoor, and Savita Lochab. 2017. “BBA - Molecular Cell Research E6AP Inhibits G-CSFR Turnover and Functions by Promoting Its Ubiquitin- Dependent Proteasome Degradation.” *BBA - Molecular Cell Research* 1864 (10): 1545–53. https://doi.org/10.1016/j.bbamcr.2017.05.026.

Chmura, Jennifer C, Kevin Herold, Ayana Ruffin, Trudymae Atuobi, Yetunde Fabiyi, Ashley E Mitchell, Young Bong Choi, and Elana S Ehrlich. 2017. “The Itch Ubiquitin Ligase Is Required for KSHV RTA Induced VFLIP Degradation.” *Virology* 501: 119–26. https://doi.org/10.1016/j.virol.2016.11.016.

Choi, Kyu-sung, Hyun-jung Choi, Jin-kyu Lee, Suhjean Im, Haiying Zhang, Yoonjeong Jeong, Jeong Ae, In-kyu Lee, Young-myeong Kim, and Young-guen Kwon. 2016. “The Endothelial E3 Ligase HECW2 Promotes Endothelial Cell Junctions by Increasing AMOTL1 Protein Stability via K63-Linked Ubiquitination.” *Cellular Signalling* 28 (11): 1642–51. https://doi.org/10.1016/j.cellsig.2016.07.015.

Choi, Young Bong, and Edward William Harhaj. 2014. “HTLV-1 Tax Stabilizes MCL-1 via TRAF6-Dependent K63- Linked Polyubiquitination to Promote Cell Survival and Transformation” 10 (10). https://doi.org/10.1371/journal.ppat.1004458.

Chu, Bernard W., Kyle M. Kovary, Johan Guillaume, Ling Chun Chen, Mary N. Teruel, and Thomas J. Wandless. 2013. “The E3 Ubiquitin Ligase UBE3C Enhances Proteasome Processivity by Ubiquitinating Partially Proteolyzed Substrates.” *Journal of Biological Chemistry* 288 (48): 34575–87. https://doi.org/10.1074/jbc.M113.499350.

Conze, Dietrich B, Lori Albert, David A Ferrick, David V Goeddel, Wen-Chen Yeh, Tak Mak, and Jonathan D Ashwell. 2005. “Posttranscriptional Downregulation of C-IAP2 by the Ubiquitin Protein Ligase c-IAP1 in Vivo.” *Molecular and Cellular Biology* 25 (8): 3348–56. https://doi.org/10.1128/MCB.25.8.3348-3356.2005.

Conze, Dietrich B, Yongge Zhao, and Jonathan D Ashwell. 2010. “Non-Canonical NF-ΚB Activation and Abnormal B Cell Accumulation in Mice Expressing Ubiquitin Protein Ligase-Inactive c-IAP2.” *PLoS Biology* 8 (10): e1000518. https://doi.org/10.1371/journal.pbio.1000518.

Coon, Tiffany A, Alison C McKelvey, Travis Lear, Shristi Rajbhandari, Sarah R Dunn, William Connelly, Joe Y Zhao, et al. 2015. “The Proinflammatory Role of HECTD2 in Innate Immunity and Experimental Lung Injury.” *Science Translational Medicine* 7 (295): 295ra109. https://doi.org/10.1126/scitranslmed.aab3881.

Cruz, C, F Ventura, R Bartrons, and J L Rosa. 2001. “HERC3 Binding to and Regulation by Ubiquitin.” *FEBS Letters* 488 (1–2): 74–80. https://doi.org/10.1016/s0014-5793(00)02371-1.

Debonneville, C, S Y Flores, E Kamynina, P J Plant, C Tauxe, M A Thomas, C Münster, et al. 2001. “Phosphorylation of Nedd4-2 by Sgk1 Regulates Epithelial Na(+) Channel Cell Surface Expression.” *The EMBO Journal* 20 (24): 7052–59. https://doi.org/10.1093/emboj/20.24.7052.

Deng, Lu, Cong Jiang, Lei Chen, Jiali Jin, Jie Wei, Linlin Zhao, Minghui Chen, et al. 2015. “The Ubiquitination of Rag A GTPase by RNF152 Negatively Regulates MTORC1 Activation.” *Molecular Cell* 58 (5): 804–18. https://doi.org/10.1016/j.molcel.2015.03.033.

Deshar, Rakesh, Song Moon, Wonjin Yoo, Eun-Bee Cho, Sungjoo K Yoon, and Jong-Bok Yoon. 2016. “RNF167 Targets Arl8B for Degradation to Regulate Lysosome Positioning and Endocytic Trafficking.” *The FEBS Journal* 283 (24): 4583–99. https://doi.org/10.1111/febs.13947.

Ding, Yi, Yan Zhang, Chao Xu, Qing-Hua Tao, and Ye-Guang Chen. 2013. “HECT Domain-Containing E3 Ubiquitin Ligase NEDD4L Negatively Regulates Wnt Signaling by Targeting Dishevelled for Proteasomal Degradation.” *The Journal of Biological Chemistry* 288 (12): 8289–98. https://doi.org/10.1074/jbc.M112.433185.

Dou, Hao, Lori Buetow, Gary J Sibbet, Kenneth Cameron, and Danny T Huang. 2012. “BIRC7-E2 Ubiquitin Conjugate Structure Reveals the Mechanism of Ubiquitin Transfer by a RING Dimer.” *Nature Structural & Molecular Biology* 19 (9): 876–83. https://doi.org/10.1038/nsmb.2379.

Du, Haijuan, Yongzhao Huang, Manar Zaghlula, Erica Walters, Timothy C Cox, and Michael A Massiah. 2013. “The MID1 E3 Ligase Catalyzes the Polyubiquitination of Alpha4 (Α4), a Regulatory Subunit of Protein Phosphatase 2A (PP2A): Novel Insights into MID1-Mediated Regulation of PP2A.” *The Journal of Biological Chemistry* 288 (29): 21341–50. https://doi.org/10.1074/jbc.M113.481093.

Du, James X., Engda G. Hagos, Mandayam O. Nandan, Agnieszka B. Bialkowska, Bing Yu, and Vincent W. Yang. 2011. “The E3 Ubiquitin Ligase SMAD Ubiquitination Regulatory Factor 2 Negatively Regulates Krüppel-like Factor 5 Protein.” *Journal of Biological Chemistry* 286 (46): 40354–64. https://doi.org/10.1074/jbc.M111.258707.

Duan, Zhijian, Bo Gao, Wei Xu, and Sidong Xiong. 2008. “Identification of TRIM22 as a RING Finger E3 Ubiquitin Ligase.” *Biochemical and Biophysical Research Communications* 374 (3): 502–6. https://doi.org/10.1016/j.bbrc.2008.07.070.

Duda, David M., Jennifer L. Olszewski, Jonathan P. Schuermann, Igor Kurinov, Darcie J. Miller, Amanda Nourse, Arno F. Alpi, and Brenda A. Schulman. 2013. “Structure of HHARI, a RING-IBR-RING Ubiquitin Ligase: Autoinhibition of an Ariadne-Family E3 and Insights into Ligation Mechanism.” *Structure* 21 (6): 1030–41. https://doi.org/10.1016/j.str.2013.04.019.

Duyvestyn, Johanna M, Samuel J Taylor, Samantha A Dagger, Marlene Orandle, Herbert C Morse, Christine B F Thien, and Wallace Y Langdon. 2014. “Dasatinib Targets B-Lineage Cells but Does Not Provide an Effective Therapy for Myeloproliferative Disease in c-Cbl RING Finger Mutant Mice.” *PloS One* 9 (4): e94717. https://doi.org/10.1371/journal.pone.0094717.

Eakin, Catherine M, Michael J Maccoss, Gregory L Finney, and Rachel E Klevit. 2007. “Estrogen Receptor Alpha Is a Putative Substrate for the BRCA1 Ubiquitin Ligase.” *Proceedings of the National Academy of Sciences of the United States of America* 104 (14): 5794–99. https://doi.org/10.1073/pnas.0610887104.

Egorova, Olga, and Yi Sheng. 2014. “Biochemical and Biophysical Research Communications A Site-Directed Mutagenesis Study of the MdmX RING Domain.” *Biochemical and Biophysical Research Communications* 447 (4): 696–701. https://doi.org/10.1016/j.bbrc.2014.04.065.

Ettenberg, Seth A., Alessandra Magnifico, Mauricio Cuello, Marion M. Nau, Yaffa R. Rubinstein, Yosef Yarden, Allan M. Weissman, and Stan Lipkowitz. 2001. “Cbl-b-Dependent Coordinated Degradation of the Epidermal Growth Factor Receptor Signaling Complex.” *Journal of Biological Chemistry* 276 (29): 27677–84. https://doi.org/10.1074/jbc.M102641200.

Fabbro, Megan, and Beric R Henderson. 2008. “BARD1 Regulates BRCA1-Mediated Transactivation of the P21WAF1/CIP1 and Gadd45 Promoters.” *Cancer Letters* 263 (2): 189–96. https://doi.org/10.1016/j.canlet.2008.01.001.

Fan, Chuandong, and Xinjiang Wang. 2017. “Mdm2 Splice Isoforms Regulate the P53 / Mdm2 / Mdm4 Regulatory Circuit via RING Domain-Mediated Ubiquitination of P53 and Mdm4.” *Cell Cycle* 16 (7): 660–64. https://doi.org/10.1080/15384101.2017.1288327.

Fan, Meiyun, Annie Park, and Kenneth P Nephew. 2005. “CHIP (Carboxyl Terminus of Hsc70-Interacting Protein) Promotes Basal and Geldanamycin-Induced Degradation of Estrogen Receptor-Alpha.” *Molecular Endocrinology (Baltimore, Md.)* 19 (12): 2901–14. https://doi.org/10.1210/me.2005-0111.

Fang, S, J P Jensen, R L Ludwig, K H Vousden, and A M Weissman. 2000. “Mdm2 Is a RING Finger-Dependent Ubiquitin Protein Ligase for Itself and P53.” *The Journal of Biological Chemistry* 275 (12): 8945–51. https://doi.org/10.1074/jbc.275.12.8945.

Faust, Tyler B, Yang Li, Gwendolyn M Jang, Jeffrey R Johnson, Shumin Yang, Amit Weiss, Nevan J Krogan, and Alan D Frankel. 2017. “PJA2 Ubiquitinates the HIV-1 Tat Protein with Atypical Chain Linkages to Activate Viral Transcription.” *Scientific Reports* 7: 45394. https://doi.org/10.1038/srep45394.

Feeney, Laura, Ivan M. Muñoz, Christophe Lachaud, Rachel Toth, Paul L. Appleton, Detlev Schindler, and John Rouse. 2017. “RPA-Mediated Recruitment of the E3 Ligase RFWD3 Is Vital for Interstrand Crosslink Repair and Human Health.” *Molecular Cell* 66 (5): 610–21. https://doi.org/10.1016/j.molcel.2017.04.021.

Fei, C., Z. Li, C. Li, Y. Chen, Z. Chen, X. He, L. Mao, X. Wang, R. Zeng, and L. Li. 2013. “Smurf1-Mediated Lys29-Linked Nonproteolytic Polyubiquitination of Axin Negatively Regulates Wnt/ -Catenin Signaling.” *Molecular and Cellular Biology* 33 (20): 4095–4105. https://doi.org/10.1128/MCB.00418-13.

Fiesel, Fabienne C., Thomas R. Caulfield, Elisabeth L. Moussaud-Lamodière, Kotaro Ogaki, Daniel F.A.R. Dourado, Samuel C. Flores, Owen A. Ross, and Wolfdieter Springer. 2015. “Structural and Functional Impact of Parkinson Disease-Associated Mutations in the E3 Ubiquitin Ligase Parkin.” *Human Mutation* 36 (8): 774–86. https://doi.org/10.1002/humu.22808.

Foglizzo, Martina, Adam J Middleton, and Catherine L Day. 2016. “Structure and Function of the RING Domains of RNF20 and RNF40 , Dimeric E3 Ligases That Monoubiquitylate Histone H2B.” *Journal of Molecular Biology* 428 (20): 4073–86. https://doi.org/10.1016/j.jmb.2016.07.025.

Forget, Antoine, Laure Bihannic, Sara Maria Cigna, Coralie Lefevre, Marc Remke, Monia Barnat, Sophie Dodier, et al. 2014. “Shh Signaling Protects Atoh1 from Degradation Mediated by the E3 Ubiquitin Ligase Huwe1 in Neural Precursors.” *Developmental Cell* 29 (6): 649–61. https://doi.org/10.1016/j.devcel.2014.05.014.

Fu, Bishi, Lingyan Wang, Hao Ding, Jens C Schwamborn, Shitao Li, and Martin E Dorf. 2015. “TRIM32 Senses and Restricts Influenza A Virus by Ubiquitination of PB1 Polymerase.” *PLoS Pathogens* 11 (6): e1004960. https://doi.org/10.1371/journal.ppat.1004960.

Funakoshi-Tago, Megumi, Noriyuki Kamada, Taeko Shimizu, Yusuke Hashiguchi, Kenji Tago, Yoshiko Sonoda, and Tadashi Kasahara. 2009. “TRAF6 Negatively Regulates TNFα-Induced NF-ΚB Activation.” *Cytokine* 45 (2): 72–79. https://doi.org/10.1016/j.cyto.2008.10.010.

Fusco, Carmela, Lucia Micale, Mikhail Egorov, Maria Monti, Ester Valentina D’Addetta, Bartolomeo Augello, Flora Cozzolino, et al. 2012. “The E3-Ubiquitin Ligase TRIM50 Interacts with HDAC6 and P62, and Promotes the Sequestration and Clearance of Ubiquitinated Proteins into the Aggresome.” *PloS One* 7 (7): e40440. https://doi.org/10.1371/journal.pone.0040440.

Gao, Jie, Mate Marosi, Jinkuk Choi, Jennifer M Achiro, Sangmok Kim, Sandy Li, Klara Otis, Kelsey C Martin, Carlos Portera-Cailliau, and Peter Tontonoz. 2017. “The E3 Ubiquitin Ligase IDOL Regulates Synaptic ApoER2 Levels and Is Important for Plasticity and Learning.” *ELife* 6 (11): e29178. https://doi.org/10.7554/eLife.29178.

Gao, Rui, Lin-Qiang Ma, Xiaogang Du, Ting-Ting Zhang, Liang Zhao, Luhong Liu, Jing-Crystal Liu, Fengjin Guo, Zhi Cheng, and Huizhe Huang. 2016. “Rnf25/AO7 Positively Regulates Wnt Signaling via Disrupting Nkd1-Axin Inhibitory Complex Independent of Its Ubiquitin Ligase Activity.” *Oncotarget* 7 (17): 23850–59. https://doi.org/10.18632/oncotarget.8126.

Gao, Rui, Lan Wang, Hao Cai, Jingjing Zhu, and Long Yu. 2016. “E3 Ubiquitin Ligase RLIM Negatively Regulates C-Myc Transcriptional Activity and Restrains Cell Proliferation.” *PloS One* 11 (9): e0164086. https://doi.org/10.1371/journal.pone.0164086.

Gao, Sheng, Claudio Alarcón, Gopal Sapkota, Sadia Rahman, Pan-Yu Chen, Nina Goerner, Maria J Macias, Hediye Erdjument-Bromage, Paul Tempst, and Joan Massagué. 2009. “Ubiquitin Ligase Nedd4L Targets Activated Smad2/3 to Limit TGF-Beta Signaling.” *Molecular Cell* 36 (3): 457–68. https://doi.org/10.1016/j.molcel.2009.09.043.

Geyer, R K, Z K Yu, and C G Maki. 2000. “The MDM2 RING-Finger Domain Is Required to Promote P53 Nuclear Export.” *Nature Cell Biology* 2 (9): 569–73. https://doi.org/10.1038/35023507.

Giardino Torchia, Maria Letizia, Ivana Munitic, Ehydel Castro, Jasmin Herz, Dorian B McGavern, and Jonathan D Ashwell. 2015. “C-IAP Ubiquitin Protein Ligase Activity Is Required for 4-1BB Signaling and CD8(+) Memory T-Cell Survival.” *European Journal of Immunology* 45 (9): 2672–82. https://doi.org/10.1002/eji.201445342.

Gmachl, M, C Gieffers, A V Podtelejnikov, M Mann, and J M Peters. 2000. “The RING-H2 Finger Protein APC11 and the E2 Enzyme UBC4 Are Sufficient to Ubiquitinate Substrates of the Anaphase-Promoting Complex.” *Proceedings of the National Academy of Sciences of the United States of America* 97 (16): 8973–78. https://doi.org/10.1073/pnas.97.16.8973.

Gopinathan, Lakshmi, Daniel B Hannon, Jeffrey M Peters, and John P Vanden Heuvel. 2009. “Regulation of Peroxisome Proliferator-Activated Receptor-Alpha by MDM2.” *Toxicological Sciences* 108 (1): 48–58. https://doi.org/10.1093/toxsci/kfn260.

Grishina, Inna, Katherina Debus, Carmen García-limones, Constanze Schneider, Amit Shresta, Carlos García, Marco A Calzado, and M Lienhard Schmitz. 2012. “Biochimica et Biophysica Acta SIAH-Mediated Ubiquitination and Degradation of Acetyl-Transferases Regulate the P53 Response and Protein Acetylation.” *BBA - Molecular Cell Research* 1823 (12): 2287–96. https://doi.org/10.1016/j.bbamcr.2012.09.011.

Groot, Reinoud E A de, Ranjani S Ganji, Ondrej Bernatik, Bethan Lloyd-Lewis, Katja Seipel, Kateřina Šedová, Zbyněk Zdráhal, et al. 2014. “Huwe1-Mediated Ubiquitylation of Dishevelled Defines a Negative Feedback Loop in the Wnt Signaling Pathway.” *Science Signaling* 7 (317): 1–11. https://doi.org/10.1126/scisignal.2004985.

Guimarães, Dimitrius Santiago, and Marcelo Damário Gomes. 2018. “Expression, Purification, and Characterization of the TRIM49 Protein.” *Protein Expression and Purification* 143: 57–61. https://doi.org/10.1016/j.pep.2017.10.014.

Gunn, Teresa M, Derek Silvius, Pooneh Bagher, Kaihua Sun, and Katherine K Walker. 2013. “MGRN1-Dependent Pigment-Type Switching Requires Its Ubiquitination Activity but Not Its Interaction with TSG101 or NEDD4.” *Pigment Cell & Melanoma Research* 26 (2): 263–68. https://doi.org/10.1111/pcmr.12059.

Habelhah, Hasem, Ian J Frew, Aaron Laine, Peter W Janes, Frederic Relaix, David Sassoon, David D L Bowtell, and Ze’ev Ronai. 2002. “Stress-Induced Decrease in TRAF2 Stability Is Mediated by Siah2.” *The EMBO Journal* 21 (21): 5756–65. https://doi.org/10.1093/emboj/cdf576.

Han, Ziying, Cari A Sagum, Mark T Bedford, Sachdev S Sidhu, Marius Sudol, and Ronald N Harty. 2016. “ITCH E3 Ubiquitin Ligase Interacts with Ebola Virus VP40 To Regulate Budding.” *Journal of Virology* 90 (20): 9163–71. https://doi.org/10.1128/JVI.01078-16.

Han, Ziying, Cari A Sagum, Fumio Takizawa, Gordon Ruthel, Corbett T Berry, Jing Kong, J Oriol Sunyer, et al. 2017. “Ubiquitin Ligase WWP1 Interacts with Ebola Virus VP40 To Regulate Egress.” *Journal of Virology* 91 (20). https://doi.org/10.1128/JVI.00812-17.

Hanoun, Naïma, Samuel Fritsch, Odile Gayet, Véronique Gigoux, Pierre Cordelier, Nelson Dusetti, Jérôme Torrisani, and Marlène Dufresne. 2014. “The E3 Ubiquitin Ligase Thyroid Hormone Receptor-Interacting Protein 12 Targets Pancreas Transcription Factor 1a for Proteasomal Degradation.” *The Journal of Biological Chemistry* 289 (51): 35593–604. https://doi.org/10.1074/jbc.M114.620104.

Harlalka, Gaurav V, Emma L Baple, Harold Cross, Simone Kühnle, Monica Cubillos-Rojas, Konstantin Matentzoglu, Michael A Patton, et al. 2013. “Mutation of HERC2 Causes Developmental Delay with Angelman-like Features.” *Journal of Medical Genetics* 50 (2): 65–73. https://doi.org/10.1136/jmedgenet-2012-101367.

Hayes, Sebastian D., Han Liu, Ewan MacDonald, Christopher M. Sanderson, Judy M. Coulson, Michael J. Clague, and Sylvie Urbé. 2012. “Direct and Indirect Control of Mitogen-Activated Protein Kinase Pathway-Associated Components, BRAP/IMP E3 Ubiquitin Ligase and CRAF/RAF1 Kinase, by the Deubiquitylating Enzyme USP15.” *Journal of Biological Chemistry* 287 (51): 43007–18. https://doi.org/10.1074/jbc.M112.386938.

He, Ying, Guili Lian, Shuyong Lin, Zhiyun Ye, and Qinxi Li. 2013. “MDM2 Inhibits Axin-Induced P53 Activation Independently of Its E3 Ligase Activity.” *PloS One* 8 (6): e67529. https://doi.org/10.1371/journal.pone.0067529.

Heidecker, Gisela, Patricia A Lloyd, Ferri Soheilian, Kunio Nagashima, and David Derse. 2007. “The Role of WWP1-Gag Interaction and Gag Ubiquitination in Assembly and Release of Human T-Cell Leukemia Virus Type 1.” *Journal of Virology* 81 (18): 9769–77. https://doi.org/10.1128/JVI.00642-07.

Ho, Shiuh-Rong, Christina S Mahanic, Yu-Ju Lee, and Weei-Chin Lin. 2014. “RNF144A, an E3 Ubiquitin Ligase for DNA-PKcs, Promotes Apoptosis during DNA Damage.” *Proceedings of the National Academy of Sciences of the United States of America* 111 (26): E2646-55. https://doi.org/10.1073/pnas.1323107111.

Hochrainer, Karin, Nadja Pejanovic, Victoria A Olaseun, Sheng Zhang, Costantino Iadecola, and Josef Anrather. 2015. “The Ubiquitin Ligase HERC3 Attenuates NF-ΚB-Dependent Transcription Independently of Its Enzymatic Activity by Delivering the RelA Subunit for Degradation.” *Nucleic Acids Research* 43 (20): 9889–9904. https://doi.org/10.1093/nar/gkv1064.

Holleman, Justine, and Adriano Marchese. 2014. “The Ubiquitin Ligase Deltex-3l Regulates Endosomal Sorting of the G Protein-Coupled Receptor CXCR4.” *Molecular Biology of the Cell* 25 (12): 1892–1904. https://doi.org/10.1091/mbc.E13-10-0612.

Hollstein, Ronja, David A Parry, Lisa Nalbach, Clare V Logan, Tim M Strom, Verity L Hartill, Ian M Carr, et al. 2015. “HACE1 Deficiency Causes an Autosomal Recessive Neurodevelopmental Syndrome.” *Journal of Medical Genetics* 52 (12): 797–803. https://doi.org/10.1136/jmedgenet-2015-103344.

Honda, R, H Tanaka, and H Yasuda. 1997. “Oncoprotein MDM2 Is a Ubiquitin Ligase E3 for Tumor Suppressor P53.” *FEBS Letters* 420 (1): 25–27. https://doi.org/10.1016/s0014-5793(97)01480-4.

Honda, R, and H Yasuda. 2000. “Activity of MDM2, a Ubiquitin Ligase, toward P53 or Itself Is Dependent on the RING Finger Domain of the Ligase.” *Oncogene* 19 (11): 1473–76. https://doi.org/10.1038/sj.onc.1203464.

Hong, Cynthia, Sarah Duit, Pilvi Jalonen, Ruud Out, Lilith Scheer, Vincenzo Sorrentino, Rima Boyadjian, et al. 2010. “The E3 Ubiquitin Ligase IDOL Induces the Degradation of the Low Density Lipoprotein Receptor Family Members VLDLR and ApoER2.” *The Journal of Biological Chemistry* 285 (26): 19720–26. https://doi.org/10.1074/jbc.M110.123729.

Hör, Simon, Tamar Ziv, Arie Admon, and Paul J Lehner. 2009. “Stable Isotope Labeling by Amino Acids in Cell Culture and Differential Plasma Membrane Proteome Quantitation Identify New Substrates for the MARCH9 Transmembrane E3 Ligase.” *Molecular & Cellular Proteomics : MCP* 8 (8): 1959–71. https://doi.org/10.1074/mcp.M900174-MCP200.

Huang, Bin, Han Zhong Pei, Hyeun-Wook Chang, and Suk-Hwan Baek. 2018. “The E3 Ubiquitin Ligase Trim13 Regulates Nur77 Stability via Casein Kinase 2α.” *Scientific Reports* 8 (1): 13895. https://doi.org/10.1038/s41598-018-32391-5.

Huang, Fang, Han Xiao, Bin-lian Sun, and Rong-ge Yang. 2013. “Biochemical and Biophysical Research Communications Characterization of TRIM62 as a RING Finger E3 Ubiquitin Ligase and Its Subcellular Localization.” *Biochemical and Biophysical Research Communications* 432 (2): 208–13. https://doi.org/10.1016/j.bbrc.2013.02.012.

Huang, Jun, Michael S Y Huen, Hongtae Kim, Charles Chung Yun Leung, J N Mark Glover, Xiaochun Yu, and Junjie Chen. 2009. “RAD18 Transmits DNA Damage Signalling to Elicit Homologous Recombination Repair.” *Nature Cell Biology* 11 (5): 592–603. https://doi.org/10.1038/ncb1865.

Hung, Albert Y, Clifford C Sung, Ilana L Brito, and Morgan Sheng. 2010. “Degradation of Postsynaptic Scaffold GKAP and Regulation of Dendritic Spine Morphology by the TRIM3 Ubiquitin Ligase in Rat Hippocampal Neurons.” *PloS One* 5 (3): e9842. https://doi.org/10.1371/journal.pone.0009842.

Inuzuka, Hiroyuki, Hidefumi Fukushima, Shavali Shaik, and Wenyi Wei. 2010. “Novel Insights into the Molecular Mechanisms Governing Mdm2 Ubiquitination and Destruction.” *Oncotarget* 1 (7): 685–90. https://doi.org/10.18632/oncotarget.101011.

Javadi, Mojib, Terri D. Richmond, Kai Huang, and Dwayne L. Barber. 2013. “CBL Linker Region and RING Finger Mutations Lead to Enhanced Granulocyte-Macrophage Colony-Stimulating Factor (GM-CSF) Signaling via Elevated Levels of JAK2 and LYN.” *Journal of Biological Chemistry* 288 (27): 19459–70. https://doi.org/10.1074/jbc.M113.475087.

Jeong, Hyung Min, Sung Ho Lee, Jinah Yum, Chang Yeol Yeo, and Kwang Youl Lee. 2014. “Smurf2 Regulates the Degradation of YY1.” *Biochimica et Biophysica Acta - Molecular Cell Research* 1843 (9): 2005–11. https://doi.org/10.1016/j.bbamcr.2014.04.023.

Ji, Lei, Bo Jiang, Xiaomo Jiang, Olga Charlat, Amy Chen, Craig Mickanin, Andreas Bauer, Wenqing Xu, Xiaoxue Yan, and Feng Cong. 2017. “The SIAH E3 Ubiquitin Ligases Promote Wnt/β-Catenin Signaling through Mediating Wnt-Induced Axin Degradation.” *Genes & Development* 31 (9): 904–15. https://doi.org/10.1101/gad.300053.117.

Jia, Xue, Hongli Zhou, Chao Wu, Qiankun Wu, Shichao Ma, Congwen Wei, Ye Cao, et al. 2017. “The Ubiquitin Ligase RNF125 Targets Innate Immune Adaptor Protein TRIM14 for Ubiquitination and Degradation.” *Journal of Immunology* 198 (12): 4652–58. https://doi.org/10.4049/jimmunol.1601322.

Jiang, Lu-Yi, Wei Jiang, Na Tian, Yan-Ni Xiong, Jie Liu, Jian Wei, Kai-Yue Wu, Jie Luo, Xiong-Jie Shi, and Bao-Liang Song. 2018. “Ring Finger Protein 145 (RNF145) Is a Ubiquitin Ligase for Sterol-Induced Degradation of HMG-CoA Reductase.” *The Journal of Biological Chemistry* 293 (11): 4047–55. https://doi.org/10.1074/jbc.RA117.001260.

Jiao, Jian, Kaihua Sun, Will P Walker, Pooneh Bagher, Christina D Cota, and Teresa M Gunn. 2009. “Abnormal Regulation of TSG101 in Mice with Spongiform Neurodegeneration.” *Biochimica et Biophysica Acta* 1792 (10): 1027–35. https://doi.org/10.1016/j.bbadis.2009.08.009.

Jin, Chaoyang, Yu An Yang, Miriam R. Anver, Nicole Morris, Xiangchun Wang, and Ying E. Zhang. 2009. “Smad Ubiquitination Regulatory Factor 2 Promotes Metastasis of Breast Cancer Cells by Enhancing Migration and Invasiveness.” *Cancer Research* 69 (3): 735–40. https://doi.org/10.1158/0008-5472.CAN-08-1463.

Jin, Hongbing, Tzu-Ting Chiou, David R Serwanski, Celia P Miralles, Noelia Pinal, and Angel L De Blas. 2014. “Ring Finger Protein 34 (RNF34) Interacts with and Promotes γ-Aminobutyric Acid Type-A Receptor Degradation via Ubiquitination of the Γ2 Subunit.” *The Journal of Biological Chemistry* 289 (42): 29420–36. https://doi.org/10.1074/jbc.M114.603068.

Joazeiro, C A, S S Wing, H Huang, J D Leverson, T Hunter, and Y C Liu. 1999. “The Tyrosine Kinase Negative Regulator C-Cbl as a RING-Type, E2-Dependent Ubiquitin-Protein Ligase.” *Science* 286 (5438): 309–12. https://doi.org/10.1126/science.286.5438.309.

Joch, Monica, Ariel R Ase, Carol X-Q Chen, Penny A MacDonald, Maria Kontogiannea, Amadou T Corera, Alexis Brice, Philippe Séguéla, and Edward A Fon. 2007. “Parkin-Mediated Monoubiquitination of the PDZ Protein PICK1 Regulates the Activity of Acid-Sensing Ion Channels.” *Molecular Biology of the Cell* 18 (8): 3105–18. https://doi.org/10.1091/mbc.e05-11-1027.

Johnson, Bethann N, Alison K Berger, Giuseppe P Cortese, and Matthew J Lavoie. 2012. “The Ubiquitin E3 Ligase Parkin Regulates the Proapoptotic Function of Bax.” *Proceedings of the National Academy of Sciences of the United States of America* 109 (16): 6283–88. https://doi.org/10.1073/pnas.1113248109.

Jones, Jessica M, and Martin Gellert. 2003. “Autoubiquitylation of the V(D)J Recombinase Protein RAG1.” *Proceedings of the National Academy of Sciences of the United States of America* 100 (26): 15446–51. https://doi.org/10.1073/pnas.2637012100.

Jung, Jin-Gyoung, Alexander Stoeck, Bin Guan, Ren-Chin Wu, Heng Zhu, Seth Blackshaw, Ie-Ming Shih, and Tian-Li Wang. 2014. “Notch3 Interactome Analysis Identified WWP2 as a Negative Regulator of Notch3 Signaling in Ovarian Cancer.” *PLoS Genetics* 10 (10): e1004751. https://doi.org/10.1371/journal.pgen.1004751.

Kallijärvi, Jukka, Ulla Lahtinen, Riikka Hämäläinen, Marita Lipsanen-Nyman, Jorma J Palvimo, and Anna-Elina Lehesjoki. 2005. “TRIM37 Defective in Mulibrey Nanism Is a Novel RING Finger Ubiquitin E3 Ligase.” *Experimental Cell Research* 308 (1): 146–55. https://doi.org/10.1016/j.yexcr.2005.04.001.

Kannemeier, Christian, Rong Liao, and Peiqing Sun. 2007. “The RING Finger Domain of MDM2 Is Essential for MDM2-Mediated TGF-Beta Resistance.” *Molecular Biology of the Cell* 18 (6): 2367–77. https://doi.org/10.1091/mbc.e06-09-0844.

Karbowski, Mariusz, Albert Neutzner, and Richard J Youle. 2007. “The Mitochondrial E3 Ubiquitin Ligase MARCH5 Is Required for Drp1 Dependent Mitochondrial Division.” *The Journal of Cell Biology* 178 (1): 71–84. https://doi.org/10.1083/jcb.200611064.

Kawai, Hidehiko, Dmitri Wiederschain, and Zhi-Min Yuan. 2003. “Critical Contribution of the MDM2 Acidic Domain to P53 Ubiquitination.” *Molecular and Cellular Biology* 23 (14): 4939–47. https://doi.org/10.1128/mcb.23.14.4939-4947.2003.

Kawashima, Akira, Tadayoshi Karasawa, Kenji Tago, Hiroaki Kimura, Ryo Kamata, Fumitake Usui-Kawanishi, Sachiko Watanabe, et al. 2017. “ARIH2 Ubiquitinates NLRP3 and Negatively Regulates NLRP3 Inflammasome Activation in Macrophages.” *Journal of Immunology* 199 (10): 3614–22. https://doi.org/10.4049/jimmunol.1700184.

Kelsall, Ian R., David M. Duda, Jennifer L. Olszewski, Kay Hofmann, Axel Knebel, Frédéric Langevin, Nicola Wood, Melanie Wightman, Brenda A. Schulman, and Arno F. Alpi. 2013. “TRIAD1 and HHARI Bind to and Are Activated by Distinct Neddylated Cullin-RING Ligase Complexes.” *EMBO Journal* 32 (21): 2848–60. https://doi.org/10.1038/emboj.2013.209.

Khouri, Elma El, Gwenaëlle Le Pavec, Michel B. Toledano, and Agnès Delaunay-Moisan. 2013. “RNF185 Is a Novel E3 Ligase of Endoplasmic Reticulum-Associated Degradation (ERAD) That Targets Cystic Fibrosis Transmembrane Conductance Regulator (CFTR).” *Journal of Biological Chemistry* 288 (43): 31177–91. https://doi.org/10.1074/jbc.M113.470500.

Kim, Joo Mi, Eun Nae Cho, Young Eun Kwon, Sung Jun Bae, Myungjin Kim, and Jae Hong Seol. 2010. “CHFR Functions as a Ubiquitin Ligase for HLTF to Regulate Its Stability and Functions.” *Biochemical and Biophysical Research Communications* 395 (4): 515–20. https://doi.org/10.1016/j.bbrc.2010.04.052.

Kim, Jun Hyun, Sun-Mi Park, Mi Ran Kang, Sue-Young Oh, Tae H Lee, Mark T Muller, and In Kwon Chung. 2005. “Ubiquitin Ligase MKRN1 Modulates Telomere Length Homeostasis through a Proteolysis of HTERT.” *Genes & Development* 19 (7): 776–81. https://doi.org/10.1101/gad.1289405.

Kim, Jung-Hoon, Soyeon Shin, Jinho Seo, Eun-Woo Lee, Manhyung Jeong, Min-Sik Lee, Hyun-Ji Han, and Jaewhan Song. 2017. “C-Terminus of HSC70-Interacting Protein (CHIP) Inhibits Adipocyte Differentiation via Ubiquitin- and Proteasome-Mediated Degradation of PPARγ.” *Scientific Reports* 7: 40023. https://doi.org/10.1038/srep40023.

Kim, Jung Bin, So Youn Kim, Byeong Mo Kim, Hunjin Lee, Insook Kim, Jeanho Yun, Yejin Jo, et al. 2013. “Identification of a Novel Anti-Apoptotic E3 Ubiquitin Ligase That Ubiquitinates Antagonists of Inhibitor of Apoptosis Proteins SMAC, HtrA2, and ARTS.” *Journal of Biological Chemistry* 288 (17): 12014–21. https://doi.org/10.1074/jbc.M112.436113.

Kim, Minsoo, Tohru Tezuka, Keiji Tanaka, and Tadashi Yamamoto. 2004. “Cbl-c Suppresses v-Src-Induced Transformation through Ubiquitin-Dependent Protein Degradation.” *Oncogene* 23 (9): 1645–55. https://doi.org/10.1038/sj.onc.1207298.

Kim, Se-yong, Dong Wook Choi, Eun-a Kim, and Cheol Yong Choi. 2009. “Stabilization of HIPK2 by Escape from Proteasomal Degradation Mediated by the E3 Ubiquitin Ligase Siah1.” *Cancer Letters* 279 (2): 177–84. https://doi.org/10.1016/j.canlet.2009.01.036.

Kim, Sewoon, and Eek-hoon Jho. 2010. “The Protein Stability of Axin, a Negative Regulator of Wnt Signaling, Is Regulated by Smad Ubiquitination Regulatory Factor 2 (Smurf2).” *The Journal of Biological Chemistry* 285 (47): 36420–26. https://doi.org/10.1074/jbc.M110.137471.

Kim, Wanyeon, Hyesook Youn, Sungmin Lee, Eungi Kim, Daehoon Kim, Jung Sub Lee, Jae-myung Lee, and Buhyun Youn. 2018. “RNF138-Mediated Ubiquitination of RpS3 Is Required for Resistance of Glioblastoma Cells to Radiation-Induced Apoptosis.” *Nature Publishing Group* 50 (1): e434-14. https://doi.org/10.1038/emm.2017.247.

Kim, Yo Han, Hanju Yoo, A. Reum Hong, Minseo Kwon, Sang Wook Kang, Kyunggon Kim, and Youngsup Song. 2018. “NEDD4L Limits CAMP Signaling through Ubiquitination of CREB-Regulated Transcription Coactivator 3.” *FASEB Journal* 32 (7): 4053–62. https://doi.org/10.1096/fj.201701406R.

Ko, Aram, Eun-Woo Lee, Jung-Yong Yeh, Mi-Ran Yang, Wonkyung Oh, Jin-San Moon, and Jaewhan Song. 2010. “MKRN1 Induces Degradation of West Nile Virus Capsid Protein by Functioning as an E3 Ligase.” *Journal of Virology* 84 (1): 426–36. https://doi.org/10.1128/JVI.00725-09.

Koliopoulos, Marios G, Diego Esposito, Evangelos Christodoulou, Ian A Taylor, and Katrin Rittinger. 2016. “Functional Role of TRIM E3 Ligase Oligomerization and Regulation of Catalytic Activity.” *The EMBO Journal* 35 (11): 1204–18. https://doi.org/10.15252/embj.201593741.

Krishnamoorthy, Vidhya, Richa Khanna, and Veena K Parnaik. 2018. “E3 Ubiquitin Ligase HECW2 Targets PCNA and Lamin B1.” *Biochimica et Biophysica Acta. Molecular Cell Research* 1865 (8): 1088–1104. https://doi.org/10.1016/j.bbamcr.2018.05.008.

Kroismayr, Renate, Ulrike Baranyi, Christian Stehlik, Andrea Dorfleutner, Bernd R Binder, and Joachim Lipp. 2004. “HERC5, a HECT E3 Ubiquitin Ligase Tightly Regulated in LPS Activated Endothelial Cells.” *Journal of Cell Science* 117 (Pt 20): 4749–56. https://doi.org/10.1242/jcs.01338.

Kubbutat, M H, R L Ludwig, A J Levine, and K H Vousden. 1999. “Analysis of the Degradation Function of Mdm2.” *Cell Growth & Differentiation* 10 (2): 87–92.

Kühnle, Simone, Ulrike Kogel, Sandra Glockzin, Andreas Marquardt, Aaron Ciechanover, Konstantin Matentzoglu, and Martin Scheffner. 2011. “Physical and Functional Interaction of the HECT Ubiquitin-Protein Ligases E6AP and HERC2.” *Journal of Biological Chemistry* 286 (22): 19410–16. https://doi.org/10.1074/jbc.M110.205211.

Kumar, Sushant, Andrea L. Talis, and Peter M. Howley. 1999. “Identification of HHR23A as a Substrate for E6-Associated Protein- Mediated Ubiquitination.” *Journal of Biological Chemistry* 274 (26): 18785–92. https://doi.org/10.1074/jbc.274.26.18785.

Kuniyoshi, Kanako, Osamu Takeuchi, Surya Pandey, Takashi Satoh, Hidenori Iwasaki, Shizuo Akira, and Taro Kawai. 2014. “Pivotal Role of RNA-Binding E3 Ubiquitin Ligase MEX3C in RIG-I-Mediated Antiviral Innate Immunity.” *Proceedings of the National Academy of Sciences of the United States of America* 111 (15): 5646–51. https://doi.org/10.1073/pnas.1401674111.

Kurokawa, Manabu, Jiyeon Kim, Joseph Geradts, Kenkyo Matsuura, Liu Liu, Xu Ran, Wenle Xia, et al. 2013. “A Network of Substrates of the E3 Ubiquitin Ligases MDM2 and HUWE1 Control Apoptosis Independently of P53.” *Science Signaling* 6 (274): ra32 LP-ra32. https://doi.org/10.1126/scisignal.2003741.

Laine, A., and Z. Ronai. 2007. “Regulation of P53 Localization and Transcription by the HECT Domain E3 Ligase WWP1.” *Oncogene* 26 (10): 1477–83. https://doi.org/10.1038/sj.onc.1209924.

Lassot, I., I. Robbins, M. Kristiansen, R. Rahmeh, F. Jaudon, M. M. Magiera, S. Mora, et al. 2010. “Trim17, a Novel E3 Ubiquitin-Ligase, Initiates Neuronal Apoptosis.” *Cell Death and Differentiation* 17 (12): 1928–41. https://doi.org/10.1038/cdd.2010.73.

Lee, Eun-woo, Min-sik Lee, Suzanne Camus, Jaewang Ghim, Mi-ran Yang, Wonkyung Oh, Nam-chul Ha, David P Lane, and Jaewhan Song. 2009. “Differential Regulation of P53 and P21 by MKRN1 E3 Ligase Controls Cell Cycle Arrest and Apoptosis.” *The EMBO Journal* 28 (14): 2100–2113. https://doi.org/10.1038/emboj.2009.164.

Lee, Jae Min, Sun Sil Choi, Yo Han Lee, Keon Woo Khim, Sora Yoon, Byung-Gyu Kim, Dougu Nam, Pann-Ghill Suh, Kyungjae Myung, and Jang Hyun Choi. 2018. “The E3 Ubiquitin Ligase TRIM25 Regulates Adipocyte Differentiation via Proteasome-Mediated Degradation of PPARγ.” *Experimental & Molecular Medicine* 50 (10): 135. https://doi.org/10.1038/s12276-018-0162-6.

Lee, Min Sik, Hyun Ji Han, Su Yeon Han, Il Young Kim, Sehyun Chae, Choong Sil Lee, Sung Eun Kim, et al. 2018. “Loss of the E3 Ubiquitin Ligase MKRN1 Represses Diet-Induced Metabolic Syndrome through AMPK Activation.” *Nature Communications* 9 (1): 3404. https://doi.org/10.1038/s41467-018-05721-4.

Lee, So Young, Juanma Ramirez, Maribel Franco, Benoît Lectez, Monika Gonzalez, Rosa Barrio, and Ugo Mayor. 2014. “Ube3a, the E3 Ubiquitin Ligase Causing Angelman Syndrome and Linked to Autism, Regulates Protein Homeostasis through the Proteasomal Shuttle Rpn10.” *Cellular and Molecular Life Sciences* 71 (14): 2747–58. https://doi.org/10.1007/s00018-013-1526-7.

Lee, Soonduck, Jinsun Kim, Samil Jung, Chengping Li, Young Yang, Keun Il Kim, Jong Seok Lim, Yonghwan Kim, Choong Il Cheon, and Myeong Sok Lee. 2015. “SIAH1-Induced P34 SEI-1 Polyubiquitination/Degradation Mediates P53 Preferential Vitamin C Cytotoxicity.” *International Journal of Oncology* 46 (3): 1377–84. https://doi.org/10.3892/ijo.2015.2840.

Lenihan, Joan A., Orthis Saha, and Paul W. Young. 2017. “Proteomic Analysis Reveals Novel Ligands and Substrates for LNX1 E3 Ubiquitin Ligase.” *PLoS ONE* 12 (11). https://doi.org/10.1371/journal.pone.0187352.

Levkowitz, Gil, Hadassa Waterman, Seth A. Ettenberg, Menachem Katz, Alexander Y. Tsygankov, Iris Alroy, Sara Lavi, et al. 1999. “Ubiquitin Ligase Activity and Tyrosine Phosphorylation Underlie Suppression of Growth Factor Signaling by C-Cbl/Sli-1.” *Molecular Cell* 4 (6): 1029–40. https://doi.org/10.1016/S1097-2765(00)80231-2.

Li, Fubing, Yang Li, Huichun Liang, Tao Xu, Yanjie Kong, Maobo Huang, Ji Xiao, et al. 2018. “HECTD3 Mediates TRAF3 Polyubiquitination and Type I Interferon Induction during Bacterial Infection.” *Journal of Clinical Investigation* 128 (9): 4148–62. https://doi.org/10.1172/JCI120406.

Li, Shan, Kefeng Lu, Jian Wang, Liguo An, Guiwen Yang, Hui Chen, Yu Cui, et al. 2010. “Ubiquitin Ligase Smurf1 Targets TRAF Family Proteins for Ubiquitination and Degradation.” *Molecular and Cellular Biochemistry* 338 (1–2): 11–17. https://doi.org/10.1007/s11010-009-0315-y.

Li, Shitao, Lingyan Wang, Bishi Fu, Michael A Berman, Alos Diallo, and Martin E Dorf. 2014. “TRIM65 Regulates MicroRNA Activity by Ubiquitination of TNRC6.” *Proceedings of the National Academy of Sciences of the United States of America* 111 (19): 6970–75. https://doi.org/10.1073/pnas.1322545111.

Li X, Yang Y, and Ashwell JD. 2002. “TNF-RII and c-IAP1 Mediate Ubiquitination and Degradation of TRAF2.” *Nature* 416 (1991): 345–47. http://www.ncbi.nlm.nih.gov/sites/pubmed.

Li, Xinchun, Li Zhong, Zhuo Wang, Huiming Chen, Dan Liao, Ruhua Zhang, Hongyu Zhang, and Tiebang Kang. 2018. “Phosphorylation of IRS4 by CK1γ2 Promotes Its Degradation by CHIP through the Ubiquitin/Lysosome Pathway.” *Theranostics* 8 (13): 3643–53. https://doi.org/10.7150/thno.26021.

Li, Y., Y. Kong, Z. Zhou, H. Chen, Z. Wang, Y. C. Hsieh, D. Zhao, et al. 2013. “The HECTD3 E3 Ubiquitin Ligase Facilitates Cancer Cell Survival by Promoting K63-Linked Polyubiquitination of Caspase-8.” *Cell Death and Disease* 4 (11). https://doi.org/10.1038/cddis.2013.464.

Lienlaf, M., F. Hayashi, F. Di Nunzio, N. Tochio, T. Kigawa, S. Yokoyama, and F. Diaz-Griffero. 2011. “Contribution of E3-Ubiquitin Ligase Activity to HIV-1 Restriction by TRIM5αrh: Structure of the RING Domain of TRIM5.” *Journal of Virology* 85 (17): 8725–37. https://doi.org/10.1128/jvi.00497-11.

Liew, Chu Wai, Huaiyu Sun, Tony Hunter, and Catherine L Day. 2010. “RING Domain Dimerization Is Essential for RNF4 Function.” *The Biochemical Journal* 431 (1): 23–29. https://doi.org/10.1042/BJ20100957.

Lin, Li, Zhenzhen Jin, Huiping Tan, Qiaoqiao Xu, Ting Peng, and He Li. 2016. “Atypical Ubiquitination by E3 Ligase WWP1 Inhibits the Proteasome-Mediated Degradation of Mutant Huntingtin.” *Brain Research* 1643 (July): 103–12. https://doi.org/10.1016/j.brainres.2016.03.027.

Lin, Qiong, Qian Dai, Hongxia Meng, Aiqin Sun, Jing Wei, Ke Peng, Chandra Childress, Miao Chen, Genbao Shao, and Wannian Yang. 2017. “The HECT E3 Ubiquitin Ligase NEDD4 Interacts with and Ubiquitylates SQSTM1 for Inclusion Body Autophagy.” *Journal of Cell Science* 130 (22): 3839–50. https://doi.org/10.1242/jcs.207068.

Linke, K, P D Mace, C A Smith, D L Vaux, J Silke, and C L Day. 2008. “Structure of the MDM2/MDMX RING Domain Heterodimer Reveals Dimerization Is Required for Their Ubiquitylation in Trans.” *Cell Death and Differentiation* 15 (5): 841–48. https://doi.org/10.1038/sj.cdd.4402309.

Liu, Bingyu, Meng Zhang, Honglei Chu, Honghai Zhang, Haifeng Wu, Guanhua Song, Peng Wang, et al. 2017. “The Ubiquitin E3 Ligase TRIM31 Promotes Aggregation and Activation of the Signaling Adaptor MAVS through Lys63-Linked Polyubiquitination.” *Nature Immunology* 18 (2): 214–24. https://doi.org/10.1038/ni.3641.

Liu, Jinyi, Dongyun Zhang, Wenjing Luo, Jianxiu Yu, Jingxia Li, Yonghui Yu, Xinhai Zhang, Jingyuan Chen, Xue Ru Wu, and Chuanshu Huang. 2012. “E3 Ligase Activity of XIAP RING Domain Is Required for XIAP-Mediated Cancer Cell Migration, but Not for Its RhoGDI Binding Activity.” *PLoS ONE* 7 (4). https://doi.org/10.1371/journal.pone.0035682.

Liu, Juan, Cen Zhang, Yuhan Zhao, Xuetian Yue, Hao Wu, Shan Huang, James Chen, et al. 2017. “Parkin Targets HIF-1α for Ubiquitination and Degradation to Inhibit Breast Tumor Progression.” *Nature Communications* 8 (1): 1823. https://doi.org/10.1038/s41467-017-01947-w.

Liu, Min, Joanne Hsu, Caleb Chan, Zichong Li, and Qiang Zhou. 2012. “The Ubiquitin Ligase Siah1 Controls ELL2 Stability and Formation of Super Elongation Complexes to Modulate Gene Transcription.” *Molecular Cell* 46 (3): 325–34. https://doi.org/10.1016/j.molcel.2012.03.007.

Liu, Shuo, Minghong Jiang, Wendie Wang, Wei Liu, Xiaoqi Song, Zhongfei Ma, Shikun Zhang, Lun Liu, Yin Liu, and Xuetao Cao. 2018. “Nuclear RNF2 Inhibits Interferon Function by Promoting K33-Linked STAT1 Disassociation from DNA.” *Nature Immunology* 19 (1): 41–52. https://doi.org/10.1038/s41590-017-0003-0.

Liu, Tao, Qin Tang, Kunpeng Liu, Huishan Wang, Rong-fu Wang, Jun Cui, Tao Liu, et al. 2016. “TRIM11 Suppresses AIM2 Inflammasome by Degrading AIM2 via P62-Dependent Selective Article TRIM11 Suppresses AIM2 Inflammasome by Degrading AIM2 via P62-Dependent Selective Autophagy.” *Cell Reports* 16 (7): 1988–2002. https://doi.org/10.1016/j.celrep.2016.07.019.

Liu, Weixiao, Yongliang Shang, and Wei Li. 2014. “Gp78 Elongates of Polyubiquitin Chains from the Distal End through the Cooperation of Its G2BR and CUE Domains.” *Scientific Reports* 4: 7138. https://doi.org/10.1038/srep07138.

Loregger, Anke, Martina Grandl, Raquel Mejías-Luque, Michael Allgäuer, Kathrin Degenhart, Verena Haselmann, Christina Oikonomou, et al. 2015. “The E3 Ligase RNF43 Inhibits Wnt Signaling Downstream of Mutated β-Catenin by Sequestering TCF4 to the Nuclear Membrane.” *Science Signaling* 8 (393): 1–13. https://doi.org/10.1126/scisignal.aac6757.

Lu, Chi Sheng, Lan N. Truong, Aaron Aslanian, Linda Z. Shi, Yongjiang Li, Patty Yi Hwa Hwang, Kwi Hye Koh, et al. 2012. “The RING Finger Protein RNF8 Ubiquitinates Nbs1 to Promote DNA Double-Strand Break Repair by Homologous Recombination.” *Journal of Biological Chemistry* 287 (52): 43984–94. https://doi.org/10.1074/jbc.M112.421545.

Luo, Xinlong, Beibei Wang, Fan Tang, Junmei Zhang, Yingming Zhao, Hui Li, and Ying Jin. 2014. “Wwp2 Targets SRG3, a Scaffold Protein of the SWI/SNF-like BAF Complex, for Ubiquitination and Degradation.” *Biochemical and Biophysical Research Communications* 443 (3): 1048–53. https://doi.org/10.1016/j.bbrc.2013.12.089.

Lv, Kaosheng, Jing Jiang, Ryan Donaghy, Christopher R. Riling, Ying Cheng, Vemika Chandra, Krasimira Rozenova, et al. 2017. “CBL Family E3 Ubiquitin Ligases Control JAK2 Ubiquitination and Stability in Hematopoietic Stem Cells and Myeloid Malignancies.” *Genes and Development* 31 (10): 1007–23. https://doi.org/10.1101/gad.297135.117.

Ma, P., X. Yang, Q. Kong, C. Li, S. Yang, Y. Li, and B. Mao. 2014. “The Ubiquitin Ligase RNF220 Enhances Canonical Wnt Signaling through USP7-Mediated Deubiquitination of β-Catenin.” *Molecular and Cellular Biology* 34 (23): 4355–66. https://doi.org/10.1128/mcb.00731-14.

Mailand, Niels, Simon Bekker-Jensen, Helene Faustrup, Fredrik Melander, Jiri Bartek, Claudia Lukas, and Jiri Lukas. 2007. “RNF8 Ubiquitylates Histones at DNA Double-Strand Breaks and Promotes Assembly of Repair Proteins.” *Cell* 131 (5): 887–900. https://doi.org/10.1016/j.cell.2007.09.040.

Mallette, Frédérick A, Francesca Mattiroli, Gaofeng Cui, Leah C Young, Michael J Hendzel, Georges Mer, Titia K Sixma, and Stéphane Richard. 2012. “RNF8- and RNF168-Dependent Degradation of KDM4A/JMJD2A Triggers 53BP1 Recruitment to DNA Damage Sites.” *The EMBO Journal* 31 (8): 1865–78. https://doi.org/10.1038/emboj.2012.47.

Marteijn, Jurgen A.F., Laurens T. Van Der Meer, Liesbeth Van Emst, Simon Van Reijmersdal, Willemijn Wissink, Theo De Witte, Joop H. Jansen, and Bert A. Van Der Reijden. 2007. “Gfi1 Ubiquitination and Proteasomal Degradation Is Inhibited by the Ubiquitin Ligase Triad1.” *Blood* 110 (9): 3128–35. https://doi.org/10.1182/blood-2006-11-058602.

Maruyama, Takeshi, Toshihiro Araki, Yosuke Kawarazaki, Isao Naguro, Susanne Heynen, Pedro Aza-Blanc, Ze’ev Ronai, Atsushi Matsuzawa, and Hidenori Ichijo. 2014. “Roquin-2 Promotes Ubiquitin-Mediated Degradation of ASK1 to Regulate Stress Responses.” *Science Signaling* 7 (309): ra8. https://doi.org/10.1126/scisignal.2004822.

Masuda, Yuji, Miki Suzuki, Hidehiko Kawai, Fumio Suzuki, and Kenji Kamiya. 2012. “Asymmetric Nature of Two Subunits of RAD18, a RING-Type Ubiquitin Ligase E3, in the Human RAD6A-RAD18 Ternary Complex.” *Nucleic Acids Research* 40 (3): 1065–76. https://doi.org/10.1093/nar/gkr805.

Matteucci, Alessandra, Maria Patron, Denis Vecellio Reane, Stefano Gastaldello, Salvatore Amoroso, Rosario Rizzuto, Marisa Brini, Anna Raffaello, and Tito Calì. 2018. “Parkin-Dependent Regulation of the MCU Complex Component MICU1.” *Scientific Reports* 8 (1): 14199. https://doi.org/10.1038/s41598-018-32551-7.

McWilliams, Thomas G., Erica Barini, Risto Pohjolan-Pirhonen, Simon P. Brooks, François Singh, Sophie Burel, Kristin Balk, et al. 2018. “Phosphorylation of Parkin at Serine 65 Is Essential for Its Activation in Vivo.” *Open Biology* 8 (11): pii. https://doi.org/10.1098/rsob.180108.

Menzies, Sam A., Norbert Volkmar, Dick J.H. van den Boomen, Richard T. Timms, Anna S. Dickson, James A. Nathan, and Paul J. Lehner. 2018. “The Sterol-Responsive RNF145 E3 Ubiquitin Ligase Mediates the Degradation of HMG-CoA Reductase Together with Gp78 and Hrd1.” *ELife* 7 (December). https://doi.org/10.7554/eLife.40009.

Miura-Shimura, Yuko, Lei Duan, Navin L Rao, Alagarsamy L Reddi, Hideki Shimura, Rob Rottapel, Brain J Druker, Alexander Tsygankov, Vimla Band, and Hamid Band. 2003. “Cbl-Mediated Ubiquitinylation and Negative Regulation of Vav.” *The Journal of Biological Chemistry* 278 (40): 38495–504. https://doi.org/10.1074/jbc.M305656200.

Molero, Juan C., Nigel Turner, Christine B.F. Thien, Wallace Y. Langdon, David E. James, and Gregory J. Cooney. 2006. “Genetic Ablation of the C-Cbl Ubiquitin Ligase Domain Results in Increased Energy Expenditure and Improved Insulin Action.” *Diabetes* 55 (12): 3411–17. https://doi.org/10.2337/db06-0955.

Moore, Darren J., Andrew B. West, Dustin A. Dikeman, Valina L. Dawson, and Ted M. Dawson. 2008. “Parkin Mediates the Degradation-Independent Ubiquitination of Hsp70.” *Journal of Neurochemistry* 105 (5): 1806–19. https://doi.org/10.1111/j.1471-4159.2008.05261.x.

Morris, Joanna R., and Ellen Solomon. 2004. “BRCA1: BARD1 Induces the Formation of Conjugated Ubiquitin Structures, Dependent on K6 of Ubiquitin, in Cells during DNA Replication and Repair.” *Human Molecular Genetics* 13 (8): 807–17. https://doi.org/10.1093/hmg/ddh095.

Mortensen, Franziska, Daniel Schneider, Tanja Barbic, Anna Sladewska-Marquardt, Simone Kühnle, Andreas Marx, and Martin Scheffner. 2015. “Role of Ubiquitin and the HPV E6 Oncoprotein in E6AP-Mediated Ubiquitination.” *Proceedings of the National Academy of Sciences of the United States of America* 112 (32): 9872–77. https://doi.org/10.1073/pnas.1505923112.

Motegi, Akira, Raman Sood, Helen Moinova, Sanford D. Markowitz, Pu Paul Liu, and Kyungjae Myung. 2006. “Human SHPRH Suppresses Genomic Instability through Proliferating Cell Nuclear Antigen Polyubiquitination.” *Journal of Cell Biology* 175 (5): 703–8. https://doi.org/10.1083/jcb.200606145.

Munakata, Tsubasa, Yuqiong Liang, Seungtaek Kim, David R. McGivern, Jon Huibregtse, Akio Nomoto, and Stanley M. Lemon. 2007. “Hepatitis C Virus Induces E6AP-Dependent Degradation of the Retinoblastoma Protein.” *PLoS Pathogens* 3 (9): 1335–47. https://doi.org/10.1371/journal.ppat.0030139.

Nakamura, Yukio, Koji Yamamoto, Xinjun He, Bungo Otsuki, Youngwoo Kim, Hiroki Murao, Tsunemitsu Soeda, et al. 2011. “Wwp2 Is Essential for Palatogenesis Mediated by the Interaction between Sox9 and Mediator Subunit 25.” *Nature Communications* 2 (1): 251. https://doi.org/10.1038/ncomms1242.

Nakatani, Yoshio, Torsten Kleffmann, Katrin Linke, Stephen M Condon, Mark G Hinds, and Catherine L Day. 2013. “Regulation of Ubiquitin Transfer by XIAP, a Dimeric RING E3 Ligase.” *The Biochemical Journal* 450 (3): 629–38. https://doi.org/10.1042/BJ20121702.

Nelson, Andrew C., and Jeffrey T. Holt. 2010. “Impact of RING and BRCT Domain Mutations on BRCA1 Protein Stability, Localization and Recruitment to DNA Damage.” *Radiation Research* 174 (1): 1–13. https://doi.org/10.1667/rr1290.1.

Nie, Jing, Melanie A. McGill, Matt Dermer, Sascha E. Dho, Cheryl D. Wolting, and C. Jane McGlade. 2002. “LNX Functions as a RING Type E3 Ubiquitin Ligase That Targets the Cell Fate Determinant Numb for Ubiquitin-Dependent Degradation.” *EMBO Journal* 21 (1–2): 93–102. https://doi.org/10.1093/emboj/21.1.93.

Ning, S., A. D. Campos, B. G. Darnay, G. L. Bentz, and J. S. Pagano. 2008. “TRAF6 and the Three C-Terminal Lysine Sites on IRF7 Are Required for Its Ubiquitination-Mediated Activation by the Tumor Necrosis Factor Receptor Family Member Latent Membrane Protein 1.” *Molecular and Cellular Biology* 28 (20): 6536–46. https://doi.org/10.1128/mcb.00785-08.

Nishikawa, Hiroyuki, Seido Ooka, Ko Sato, Kei Arima, Joji Okamoto, Rachel E Klevit, Mamoru Fukuda, and Tomohiko Ohta. 2004. “Mass Spectrometric and Mutational Analyses Reveal Lys-6-Linked Polyubiquitin Chains Catalyzed by BRCA1-BARD1 Ubiquitin Ligase.” *The Journal of Biological Chemistry* 279 (6): 3916–24. https://doi.org/10.1074/jbc.M308540200.

Nishiyama, Atsuya, Luna Yamaguchi, Jafar Sharif, Yoshikazu Johmura, Takeshi Kawamura, Keiko Nakanishi, Shintaro Shimamura, et al. 2013. “Uhrf1-Dependent H3K23 Ubiquitylation Couples Maintenance DNA Methylation and Replication.” *Nature* 502 (7470): 249–53. https://doi.org/10.1038/nature12488.

Nomura, Koji, Marta Klejnot, Dominika Kowalczyk, Andreas K. Hock, Gary J. Sibbet, Karen H. Vousden, and Danny T. Huang. 2017. “Structural Analysis of MDM2 RING Separates Degradation from Regulation of P53 Transcription Activity.” *Nature Structural and Molecular Biology* 24 (7): 578–87. https://doi.org/10.1038/nsmb.3414.

Oksvold, Morten P., Samantha A. Dagger, Christine B.F. Thien, and Wallace Y. Langdon. 2008. “The Cbl-b RING Finger Domain Has a Limited Role in Regulating Inflammatory Cytokine Production by IgE-Activated Mast Cells.” *Molecular Immunology* 45 (4): 925–36. https://doi.org/10.1016/j.molimm.2007.08.002.

Okumura, Fumihiko, Shigetsugu Hatakeyama, Masaki Matsumoto, Takumi Kamura, and Keiichi I Nakayama. 2004. “Functional Regulation of FEZ1 by the U-Box-Type Ubiquitin Ligase E4B Contributes to Neuritogenesis.” *The Journal of Biological Chemistry* 279 (51): 53533–43. https://doi.org/10.1074/jbc.M402916200.

Oshikawa, Gaku, Toshikage Nagao, Nan Wu, Tetsuya Kurosu, and Osamu Miura. 2011. “C-Cbl and Cbl-b Ligases Mediate 17-Allylaminodemethoxygeldanamycin-Induced Degradation of Autophosphorylated Flt3 Kinase with Internal Tandem Duplication through the Ubiquitin Proteasome Pathway.” *Journal of Biological Chemistry* 286 (35): 30263–73. https://doi.org/10.1074/jbc.M111.232348.

Pal, P, S Lochab, J K Kanaujiya, I Kapoor, S Sanyal, G Behre, and A K Trivedi. 2013. “E6AP , an E3 Ubiquitin Ligase Negatively Regulates Granulopoiesis by Targeting Transcription Factor C / EBP a for Ubiquitin-Mediated Proteasome Degradation.” *Cell Death and Disease* 4 (4): e590-12. https://doi.org/10.1038/cddis.2013.120.

Palicharla, Vivek Reddy, and Subbareddy Maddika. 2015. “HACE1 Mediated K27 Ubiquitin Linkage Leads to YB-1 Protein Secretion.” *Cellular Signalling* 27 (12): 2355–62. https://doi.org/10.1016/j.cellsig.2015.09.001.

Palmada, M., M. Dieter, A. Speil, C. Böhmer, A. F. Mack, H. J. Wagner, K. Klingel, et al. 2004. “Regulation of Intestinal Phosphate Cotransporter NaPi IIb by Ubiquitin Ligase Nedd4-2 and by Serum- and Glucocorticoid-Dependent Kinase 1.” *American Journal of Physiology - Gastrointestinal and Liver Physiology* 287 (1 50-1). https://doi.org/10.1152/ajpgi.00121.2003.

Pan, Yu, Rui Li, Jun-Ling Meng, He-Ting Mao, Yu Zhang, and Jun Zhang. 2014. “Smurf2 Negatively Modulates RIG-I–Dependent Antiviral Response by Targeting VISA/MAVS for Ubiquitination and Degradation.” *The Journal of Immunology* 192 (10): 4758–64. https://doi.org/10.4049/jimmunol.1302632.

Park, Yong Yea, Seungmin Lee, Mariusz Karbowski, Albert Neutzner, Richard J. Youle, and Hyeseong Cho. 2010. “Loss of MARCH5 Mitochondrial E3 Ubiquitin Ligase Induces Cellular Senescence through Dynamin-Related Protein 1 and Mitofusin 1.” *Journal of Cell Science* 123 (4): 619–26. https://doi.org/10.1242/jcs.061481.

Park, Yoon, Sungjoo Kim Yoon, and Jong Bok Yoon. 2009. “The HECT Domain of TRIP12 Ubiquitinates Substrates of the Ubiquitin Fusion Degradation Pathway.” *Journal of Biological Chemistry* 284 (3): 1540–49. https://doi.org/10.1074/jbc.M807554200.

Pettersson, Susanne, Michael Kelleher, Emmanuelle Pion, Maura Wallace, and Kathryn L. Ball. 2009. “Role of Mdm2 Acid Domain Interactions in Recognition and Ubiquitination of the Transcription Factor IRF-2.” *Biochemical Journal* 418 (3): 575–85. https://doi.org/10.1042/BJ20082087.

Pietschmann, Kristin, Marc Buchwald, Sylvia Müller, Shirley K Knauer, Manfred Kögl, Thorsten Heinzel, and Oliver H Krämer. 2012. “The International Journal of Biochemistry Differential Regulation of PML – RAR ␣ Stability by the Ubiquitin Ligases.” *International Journal of Biochemistry and Cell Biology* 44 (1): 132–38. https://doi.org/10.1016/j.biocel.2011.10.008.

Pinato, Sabrina, Cristina Scandiuzzi, Nadia Arnaudo, Elisabetta Citterio, Giovanni Gaudino, and Lorenza Penengo. 2009. “RNF168, a New RING Finger, MIU-Containing Protein That Modifies Chromatin by Ubiquitination of Histones H2A and H2AX.” *BMC Molecular Biology* 10 (June). https://doi.org/10.1186/1471-2199-10-55.

Poyurovsky, Masha V., Christina Priest, Alex Kentsis, Katherine L.B. Borden, Zhen Qiang Pan, Nikola Pavletich, and Carol Prives. 2007. “The Mdm2 RING Domain C-Terminus Is Required for Supramolecular Assembly and Ubiquitin Ligase Activity.” *EMBO Journal* 26 (1): 90–101. https://doi.org/10.1038/sj.emboj.7601465.

Qin, Yue, Mao-Tian Zhou, Ming-Ming Hu, Yun-Hong Hu, Jing Zhang, Lin Guo, Bo Zhong, and Hong-Bing Shu. 2014. “RNF26 Temporally Regulates Virus-Triggered Type I Interferon Induction by Two Distinct Mechanisms.” *PLoS Pathogens* 10 (9): e1004358. https://doi.org/10.1371/journal.ppat.1004358.

Raheja, Radhika, Yuhui Liu, Ellen Hukkelhoven, Nancy Yeh, and Andrew Koff. 2014. “The Ability of TRIM3 to Induce Growth Arrest Depends on RING-Dependent E3 Ligase Activity.” *Biochemical Journal* 458 (3): 537–45. https://doi.org/10.1042/BJ20131288.

Rai, Rekha, Ju Mei Li, Hong Zheng, Gabriel Tsz Mei Lok, Yu Deng, Michael S.Y. Huen, Junjie Chen, Jianping Jin, and Sandy Chang. 2011. “The E3 Ubiquitin Ligase Rnf8 Stabilizes Tpp1 to Promote Telomere End Protection.” *Nature Structural and Molecular Biology* 18 (12): 1400–1407. https://doi.org/10.1038/nsmb.2172.

Ran, Yong, Jing Zhang, Li Li Liu, Zhao Yi Pan, Ying Nie, Hong Yan Zhang, and Yan Yi Wang. 2016. “Autoubiquitination of TRIM26 Links TBK1 to NEMO in RLR-Mediated Innate Antiviral Immune Response.” *Journal of Molecular Cell Biology* 8 (1): 31–43. https://doi.org/10.1093/jmcb/mjv068.

Rathinam, Chozhavendan, Christine B F Thien, Richard A Flavell, and Wallace Y Langdon. 2010. “Article Myeloid Leukemia Development in C-Cbl RING Finger Mutant Mice Is Dependent on FLT3 Signaling.” *Cancer Cell* 18 (4): 341–52. https://doi.org/10.1016/j.ccr.2010.09.008.

Riley, B E, J C Lougheed, K Callaway, M Velasquez, E Brecht, L Nguyen, T Shaler, et al. 2013. “Structure and Function of Parkin E3 Ubiquitin Ligase Reveals Aspects of RING and HECT Ligases.” *Nature Communications* 4 (May): 1–9. https://doi.org/10.1038/ncomms2982.

Ruffner, H, C A Joazeiro, D Hemmati, T Hunter, and I M Verma. 2001. “Cancer-Predisposing Mutations within the RING Domain of BRCA1: Loss of Ubiquitin Protein Ligase Activity and Protection from Radiation Hypersensitivity.” *Proceedings of the National Academy of Sciences of the United States of America* 98 (9): 5134–39. https://doi.org/10.1073/pnas.081068398.

Ryu, Yeung Sook, Younglang Lee, Keun Woo Lee, Chae Young Hwang, Jin Soo Maeng, Jeong Hoon Kim, Yeon Soo Seo, Kwan Hee You, Byeongwoon Song, and Ki Sun Kwon. 2011. “TRIM32 Protein Sensitizes Cells to Tumor Necrosis Factor (TNFα)-Induced Apoptosis via Its RING Domain-Dependent E3 Ligase Activity against X-Linked Inhibitor of Apoptosis (XIAP).” *Journal of Biological Chemistry* 286 (29): 25729–38. https://doi.org/10.1074/jbc.M111.241893.

Sakai, Ryohei, Ryosuke Fukuda, Shin Unida, Misaki Aki, Yuji Ono, Akinori Endo, Satoshi Kusumi, et al. 2019. “The Integral Function of the Endocytic Recycling Compartment Is Regulated by RFFL-Mediated Ubiquitylation of Rab11 Effectors.” *Journal of Cell Science* 132 (3). https://doi.org/10.1242/jcs.228007.

Sankaran, Satish, Lea M. Starita, Amanda M. Simons, and Jeffrey D. Parvin. 2006. “Identification of Domains of BRCA1 Critical for the Ubiquitin-Dependent Inhibition of Centrosome Function.” *Cancer Research* 66 (8): 4100–4107. https://doi.org/10.1158/0008-5472.CAN-05-4430.

Sarkar, Anjali A., and Irene E. Zohn. 2012. “Hectd1 Regulates Intracellular Localization and Secretion of Hsp90 to Control Cellular Behavior of the Cranial Mesenchyme.” *Journal of Cell Biology* 196 (6): 789–800. https://doi.org/10.1083/jcb.201105101.

Sarraf, Shireen A., Malavika Raman, Virginia Guarani-Pereira, Mathew E. Sowa, Edward L. Huttlin, Steven P. Gygi, and J. Wade Harper. 2013. “Landscape of the PARKIN-Dependent Ubiquitylome in Response to Mitochondrial Depolarization.” *Nature* 496 (7445): 372–76. https://doi.org/10.1038/nature12043.

Sasaki, Takanori, Hirotada Kojima, Rikiya Kishimoto, Ayu Ikeda, Hiroyuki Kunimoto, and Koich Nakajima. 2006. “Spatiotemporal Regulation of C-Fos by ERK5 and the E3 Ubiquitin Ligase UBR1, and Its Biological Role.” *Molecular Cell* 24 (1): 63–75. https://doi.org/10.1016/j.molcel.2006.08.005.

Scott, Daniel C, David Y Rhee, David M Duda, Ian R Kelsall, Jennifer L Olszewski, Joao A Paulo, Annemieke de Jong, et al. 2016. “Two Distinct Types of E3 Ligases Work in Unison to Regulate Substrate Ubiquitylation.” *Cell* 166 (5): 1198–1214.e24. https://doi.org/10.1016/j.cell.2016.07.027.

Seo, Jae Ho, Ekta Agarwal, Kelly G. Bryant, M. Cecilia Caino, Eui Tae Kim, Andrew V. Kossenkov, Hsin Yao Tang, et al. 2018. “Syntaphilin Ubiquitination Regulates Mitochondrial Dynamics and Tumor Cell Movements.” *Cancer Research* 78 (15): 4215–28. https://doi.org/10.1158/0008-5472.CAN-18-0595.

Shen, Jiajia, Pengyu Li, Xuejing Shao, Yang Yang, Xiujun Liu, Min Feng, Qiang Yu, Ronggui Hu, and Zhen Wang. 2018. “The E3 Ligase Ring1 Targets P53 for Degradation and Promotes Cancer Cell Proliferation and Survival.” *Cancer Research* 78 (2): 359–71. https://doi.org/10.1158/0008-5472.CAN-17-1805.

Shi, H. X., K. Yang, X. Liu, X. Y. Liu, B. Wei, Y. F. Shan, L. H. Zhu, and C. Wang. 2010. “Positive Regulation of Interferon Regulatory Factor 3 Activation by Herc5 via ISG15 Modification.” *Molecular and Cellular Biology* 30 (10): 2424–36. https://doi.org/10.1128/mcb.01466-09.

Shimamoto, Seiko, Yasuo Kubota, Fuminori Yamaguchi, Hiroshi Tokumitsu, and Ryoji Kobayashi. 2013. “Ca 2+ /S100 Proteins Act as Upstream Regulators of the Chaperone-Associated Ubiquitin Ligase Chip (c Terminus of Hsc70-Interacting Protein).” *Journal of Biological Chemistry* 288 (10): 7158–68. https://doi.org/10.1074/jbc.M112.436758.

Shoji, Shisako, Kazuharu Hanada, Noboru Ohsawa, and Mikako Shirouzu. 2017. “Central Catalytic Domain of BRAP (RNF52) Recognizes the Types of Ubiquitin Chains and Utilizes Oligo-Ubiquitin for Ubiquitylation.” *Biochemical Journal* 474 (18): 3207–26. https://doi.org/10.1042/bcj20161104.

Shukla, Shirish, Uday Sankar Allam, Aarif Ahsan, Guoan Chen, Pranathi Meda Krishnamurthy, Katherine Marsh, Matthew Rumschlag, et al. 2014. “KRAS Protein Stability Is Regulated through SMURF2: UBCH5 Complex-Mediated β-TrCP1 Degradation.” *Neoplasia (United States)* 16 (2): 115–28. https://doi.org/10.1593/neo.14184.

Smit, Judith J, Davide Monteferrario, Sylvie M Noordermeer, Willem J Van, Bert A Van Der Reijden, and Titia K Sixma. 2012. “The E3 Ligase HOIP Specifies Linear Ubiquitin Chain Assembly through Its RING-IBR-RING Domain and the Unique LDD Extension.” *The EMBO Journal* 31 (19): 3833–44. https://doi.org/10.1038/emboj.2012.217.

Song, Fei, Chuandong Fan, Xinjiang Wang, and David W. Goodrich. 2013. “The Thoc1 Encoded Ribonucleoprotein Is a Substrate for the NEDD4-1 E3 Ubiquitin Protein Ligase.” *PLoS ONE* 8 (2). https://doi.org/10.1371/journal.pone.0057995.

Song, Hui, Bingyu Liu, Wanwan Huai, Zhongxia Yu, Wenwen Wang, Jing Zhao, Lihui Han, et al. 2016. “The E3 Ubiquitin Ligase TRIM31 Attenuates NLRP3 Inflammasome Activation by Promoting Proteasomal Degradation of NLRP3.” *Nature Communications* 7 (December). https://doi.org/10.1038/ncomms13727.

Song, Pingping, Katarina Trajkovic, Taiji Tsunemi, and Dimitri Krainc. 2016. “Parkin Modulates Endosomal Organization and Function of the Endo-Lysosomal Pathway.” *Journal of Neuroscience* 36 (8): 2425–37. https://doi.org/10.1523/JNEUROSCI.2569-15.2016.

Sorrentino, Vincenzo, Lilith Scheer, Ana Santos, Eric Reits, Boris Bleijlevens, and Noam Zelcer. 2011. “Distinct Functional Domains Contribute to Degradation of the Low Density Lipoprotein Receptor (LDLR) by the E3 Ubiquitin Ligase Inducible Degrader of the LDLR (IDOL).” *Journal of Biological Chemistry* 286 (34): 30190–99. https://doi.org/10.1074/jbc.M111.249557.

Sparrer, Konstantin M.J., Sebastian Gableske, Matthew A. Zurenski, Zachary M. Parker, Florian Full, Gavin J. Baumgart, Jiro Kato, et al. 2017. “TRIM23 Mediates Virus-Induced Autophagy via Activation of TBK1.” *Nature Microbiology* 2 (11): 1543–57. https://doi.org/10.1038/s41564-017-0017-2.

Stechow, Louise von, Dimitris Typas, Jordi Carreras Puigvert, Laurens Oort, Ramakrishnaiah Siddappa, Alex Pines, Harry Vrieling, Bob van de Water, Leon H. F. Mullenders, and Erik H. J. Danen. 2015. “The E3 Ubiquitin Ligase ARIH1 Protects against Genotoxic Stress by Initiating a 4EHP-Mediated MRNA Translation Arrest.” *Molecular and Cellular Biology* 35 (7): 1254–68. https://doi.org/10.1128/mcb.01152-14.

Stewart, Mikaela D, Emily D Duncan, Ernesto Coronado, Paul A DaRosa, Jonathan N Pruneda, Peter S Brzovic, and Rachel E Klevit. 2017. “Tuning BRCA1 and BARD1 Activity to Investigate RING Ubiquitin Ligase Mechanisms.” *Protein Science : A Publication of the Protein Society* 26 (3): 475–83. https://doi.org/10.1002/pro.3091.

Sugeno, Naoto, Takafumi Hasegawa, Nobuyuki Tanaka, Mitsunori Fukuda, Koichi Wakabayashi, Ryuji Oshima, Masashi Konno, et al. 2014. “Lys-63-Linked Ubiquitination by E3 Ubiquitin Ligase Nedd4-1 Facilitates Endosomal Sequestration of Internalized α-Synuclein.” *Journal of Biological Chemistry* 289 (26): 18137–51. https://doi.org/10.1074/jbc.M113.529461.

Sugrue, Kelsey F., Anjali A. Sarkar, Linda Leatherbury, and Irene E. Zohn. 2019. “The Ubiquitin Ligase HECTD1 Promotes Retinoic Acid Signaling Required for Development of the Aortic Arch.” *DMM Disease Models and Mechanisms* 12 (1). https://doi.org/10.1242/dmm.036491.

Tajima, Yoshitaka, Kouichiro Goto, Minoru Yoshida, Kenichi Shinomiya, Toshihiro Sekimoto, Yoshihiro Yoneda, Kohei Miyazono, and Takeshi Imamura. 2003. “Chromosomal Region Maintenance 1 (CRM1)-Dependent Nuclear Export of Smad Ubiquitin Regulatory Factor 1 (Smurf1) Is Essential for Negative Regulation of Transforming Growth Factor-β Signaling by Smad7.” *Journal of Biological Chemistry* 278 (12): 10716–21. https://doi.org/10.1074/jbc.M212663200.

Tan, Cyrus, Eamon F X Byrne, Casey Ah-Cann, Melissa J Call, and Matthew E Call. 2019. “A Serine in the First Transmembrane Domain of the Human E3 Ubiquitin Ligase MARCH9 Is Critical for Down-Regulation of Its Protein Substrates.” *The Journal of Biological Chemistry* 294 (7): 2470–85. https://doi.org/10.1074/jbc.RA118.004836.

Tanabe, Chiaki, Tomoji Maeda, Kun Zou, Junjun Liu, Shuyu Liu, Toshihiro Nakajima, and Hiroto Komano. 2012. “The Ubiquitin Ligase Synoviolin Up-Regulates Amyloid β Production by Targeting a Negative Regulator of γ-Secretase, Rer1, for Degradation.” *Journal of Biological Chemistry* 287 (53): 44203–11. https://doi.org/10.1074/jbc.M112.365296.

Tateishi, Satoshi, Oshiyuki Sakuraba, Sadaharu Masuyama, Rokazu Inoue, and Masaru Yamaizumi. 2000. “Dysfunction of Human Rad18 Results in Defective Postreplication Repair and Hypersensitivity to Multiple Mutagens.” *Proceedings of the National Academy of Sciences of the United States of America* 97 (14): 7927–32. https://doi.org/10.1073/pnas.97.14.7927.

Taylor, Samuel J, Christine B F Thien, Samantha A Dagger, Johanna M Duyvestyn, Carolyn S Grove, Benjamin H Lee, D Gary Gilliland, and Wallace Y Langdon. 2015. “Loss of C-Cbl E3 Ubiquitin Ligase Activity Enhances the Development of Myeloid Leukemia in FLT3-ITD Mutant Mice.” *Experimental Hematology* 43 (3): 191–206.e1. https://doi.org/10.1016/j.exphem.2014.11.009.

Theivanthiran, Balamayooran, Mahesh Kathania, Minghui Zeng, Esperanza Anguiano, Venkatesha Basrur, Travis Vandergriff, Virginia Pascual, Wei Zen Wei, Ramin Massoumi, and K. Venuprasad. 2015. “The E3 Ubiquitin Ligase Itch Inhibits P38α Signaling and Skin Inflammation through the Ubiquitylation of Tab1.” *Science Signaling* 8 (365). https://doi.org/10.1126/scisignal.2005903.

Thien, Christine B.F., Frøydis D. Blystad, Yifan Zhan, Andrew M. Lew, Valentina Voigt, Christopher E. Andoniou, and Wallace Y. Langdon. 2005. “Loss of C-Cbl RING Finger Function Results in High-Intensity TCR Signaling and Thymic Deletion.” *EMBO Journal* 24 (21): 3807–19. https://doi.org/10.1038/sj.emboj.7600841.

Thien, Christine B.F., Francesca Walker, and Wallace Y. Langdon. 2001. “RING Finger Mutations That Abolish C-Cbl-Directed Polyubiquitination and Downregulation of the EGF Receptor Are Insufficient for Cell Transformation.” *Molecular Cell* 7 (2): 355–65. https://doi.org/10.1016/S1097-2765(01)00183-6.

Tian, Hui, Nicole R. Tackmann, Aiwen Jin, Junnian Zheng, and Yanping Zhang. 2017. “Inactivation of the MDM2 RING Domain Enhances P53 Transcriptional Activity in Mice.” *Journal of Biological Chemistry* 292 (52): 21614–22. https://doi.org/10.1074/jbc.RA117.000122.

Torrino, Stéphanie, Orane Visvikis, Anne Doye, Laurent Boyer, Caroline Stefani, Patrick Munro, Jacques Bertoglio, Gérard Gacon, Amel Mettouchi, and Emmanuel Lemichez. 2011. “The E3 Ubiquitin-Ligase HACE1 Catalyzes the Ubiquitylation of Active Rac1.” *Developmental Cell* 21 (5): 959–65. https://doi.org/10.1016/j.devcel.2011.08.015.

Toshiyuki, Araki, and Jeffrey Milbrandt. 2003. “ZNRF Proteins Constitute a Family of Presynaptic E3 Ubiquitin Ligases.” *The Journal of Neuroscience : The Official Journal of the Society for Neuroscience* 23 (28): 9385–94.

Tran, Hoanh, Daisy Bustos, Ronald Yeh, Bonnee Rubinfeld, Cynthia Lam, Stephanie Shriver, Inna Zilberleyb, et al. 2013. “HectDI E3 Ligase Modifies Adenomatous Polyposis Coli (APC) with Polyubiquitin to Promote the APC-Axin Interaction.” *Journal of Biological Chemistry* 288 (6): 3753–67. https://doi.org/10.1074/jbc.M112.415240.

Tripathi, Ekta, and Susan Smith. 2017. “Cell Cycle‐regulated Ubiquitination of Tankyrase 1 by RNF8 and ABRO1/BRCC36 Controls the Timing of Sister Telomere Resolution.” *The EMBO Journal* 36 (4): 503–19. https://doi.org/10.15252/embj.201695135.

Uchida, Chiharu, Seiichi Miwa, Kyoko Kitagawa, Takayuki Hattori, Tomoyasu Isobe, Sunao Otani, Toshiaki Oda, et al. 2005. “Enhanced Mdm2 Activity Inhibits PRB Function via Ubiquitin-Dependent Degradation.” *EMBO Journal* 24 (1): 160–69. https://doi.org/10.1038/sj.emboj.7600486.

Wada, Keiji, and Tetsu Kamitani. 2006. “Autoantigen Ro52 Is an E3 Ubiquitin Ligase.” *Biochemical and Biophysical Research Communications* 339 (1): 415–21. https://doi.org/10.1016/j.bbrc.2005.11.029.

Wang, Hong-Rui, Abiodun A Ogunjimi, Yue Zhang, Barish Ozdamar, Rohit Bose, and Jeffrey L Wrana. 2006. “Degradation of RhoA by Smurf1 Ubiquitin Ligase.” *Methods in Enzymology* 406: 437–47. https://doi.org/10.1016/S0076-6879(06)06032-0.

Wang, Qiang, Xing Liu, Ye Cui, Yijun Tang, Wei Chen, Senlin Li, Huansha Yu, Youdong Pan, and Chen Wang. 2014. “The E3 Ubiquitin Ligase AMFR and INSIG1 Bridge the Activation of TBK1 Kinase by Modifying the Adaptor STING.” *Immunity* 41 (6): 919–33. https://doi.org/10.1016/j.immuni.2014.11.011.

Wang, Wei, Zhi-Jie Xia, Jean-Claude Farré, and Suresh Subramani. 2017. “TRIM37, a Novel E3 Ligase for PEX5-Mediated Peroxisomal Matrix Protein Import.” *The Journal of Cell Biology* 216 (9): 2843–58. https://doi.org/10.1083/jcb.201611170.

Wang, Xiangchun, Chaoyang Jin, Yi Tang, Liu Ya Tang, and Ying E. Zhang. 2013. “Ubiquitination of Tumor Necrosis Factor Receptor-Associated Factor 4 (TRAF4) by Smad Ubiquitination Regulatory Factor 1 (Smurf1) Regulates Motility of Breast Epithelial and Cancer Cells.” *Journal of Biological Chemistry* 288 (30): 21784–92. https://doi.org/10.1074/jbc.M113.472704.

Wang, Xiaozhen, Guang Lu, Li Li, Juan Yi, Kaowen Yan, Yaqing Wang, Baili Zhu, et al. 2014. “HUWE1 Interacts with BRCA1 and Promotes Its Degradation in the Ubiquitin-Proteasome Pathway.” *Biochemical and Biophysical Research Communications* 444 (4): 549–54. https://doi.org/10.1016/j.bbrc.2014.01.075.

Wang, Xinjiang, Yuji Shi, Junru Wang, Guochang Huang, and Xuejun Jiang. 2008. “Crucial Role of the C-Terminus of PTEN in Antagonizing NEDD4-1-Mediated PTEN Ubiquitination and Degradation.” *Biochemical Journal* 414 (2): 221–29. https://doi.org/10.1042/BJ20080674.

Wang, Yu, Bing Shan, Yaosi Liang, Huiting Wei, and Junying Yuan. 2018. “Parkin Regulates NF-ΚB by Mediating Site-Specific Ubiquitination of RIPK1.” *Cell Death and Disease* 9 (7): 732. https://doi.org/10.1038/s41419-018-0770-z.

Waterman, Hadassa, Gil Levkowitz, Iris Alroy, and Yosef Yarden. 1999. “The RING Finger of C-Cbl Mediates Desensitization of the Epidermal Growth Factor Receptor.” *Journal of Biological Chemistry* 274 (32): 22151–54. https://doi.org/10.1074/jbc.274.32.22151.

Wauer, Tobias, Michal Simicek, Alexander Schubert, and David Komander. 2015. “Mechanism of Phospho-Ubiquitin-Induced PARKIN Activation.” *Nature* 524 (7565): 370–74. https://doi.org/10.1038/nature14879.

Wawrzynow, Bartosz, Susanne Pettersson, Alicja Zylicz, Janice Bramham, Erin Worrall, Ted R. Hupp, and Kathryn L. Ball. 2009. “A Function for the RING Finger Domain in the Allosteric Control of MDM2 Conformation and Activity.” *Journal of Biological Chemistry* 284 (17): 11517–30. https://doi.org/10.1074/jbc.M809294200.

Wei, Ping, Jihui Guo, Wen Xue, Yun Zhao, Jinbo Yang, and Jiwu Wang. 2018. “RNF34 Modulates the Mitochondrial Biogenesis and Exercise Capacity in Muscle and Lipid Metabolism through Ubiquitination of PGC-1 in Drosophila.” *Acta Biochimica et Biophysica Sinica* 50 (10): 1038–46. https://doi.org/10.1093/abbs/gmy106.

Wenzel, Dawn M, Alexei Lissounov, Peter S Brzovic, and Rachel E Klevit. 2011. “UBCH7 Reactivity Profile Reveals Parkin and HHARI to Be RING/HECT Hybrids.” *Nature* 474 (7349): 105–8. https://doi.org/10.1038/nature09966.

Williams, Stacy A., Simonne Longerich, Patrick Sung, Cyrus Vaziri, and Gary M. Kupfer. 2011. “The E3 Ubiquitin Ligase RAD18 Regulates Ubiquitylation and Chromatin Loading of FANCD2 and FANCI.” *Blood* 117 (19): 5078–87. https://doi.org/10.1182/blood-2010-10-311761.

Wolting, Cheryl D., Emily K. Griffiths, Renu Sarao, Brittany C. Prevost, Leanne E. Wybenga-Groot, and C. Jane McGlade. 2011. “Biochemical and Computational Analysis of LNX1 Interacting Proteins.” *PLoS ONE* 6 (11). https://doi.org/10.1371/journal.pone.0026248.

Wong, Joyce Jing Yi, Yuh Fen Pung, Newman Siu Kwan Sze, and Keh Chuang Chin. 2006. “HERC5 Is an IFN-Induced HECT-Type E3 Protein Ligase That Mediates Type I IFN-Induced ISGylation of Protein Targets.” *Proceedings of the National Academy of Sciences of the United States of America* 103 (28): 10735–40. https://doi.org/10.1073/pnas.0600397103.

Wu, Wenwen, Ko Sato, Ayaka Koike, Hiroyuki Nishikawa, Hirotaka Koizumi, Ashok R Venkitaraman, and Tomohiko Ohta. 2010. “HERC2 Is an E3 Ligase That Targets BRCA1 for Degradation.” *Cancer Research* 70 (15): 6384–92. https://doi.org/10.1158/0008-5472.CAN-10-1304.

Xia, Pengyan, Shuo Wang, Guanling Huang, Ying Du, Pingping Zhu, Man Li, and Zusen Fan. 2014. “RNF2 Is Recruited by WASH to Ubiquitinate AMBRA1 Leading to Downregulation of Autophagy.” *Nature Publishing Group* 24 (8): 943–58. https://doi.org/10.1038/cr.2014.85.

Xin, Di, Haiyan Gu, Enping Liu, and Qinmiao Sun. 2018. “Parkin Negatively Regulates the Antiviral Signaling Pathway by Targeting TRAF3 for Degradation.” *Journal of Biological Chemistry* 293 (31): 11996–10. https://doi.org/10.1074/jbc.RA117.001201.

Xiong, Ying, Da Song, Yunfei Cai, Wenfeng Yu, Yee Guide Yeung, and E. Richard Stanley. 2011. “A CSF-1 Receptor Phosphotyrosine 559 Signaling Pathway Regulates Receptor Ubiquitination and Tyrosine Phosphorylation.” *Journal of Biological Chemistry* 286 (2): 952–60. https://doi.org/10.1074/jbc.M110.166702.

Xu, Da, Haoxun Wang, Qiang Zhang, and Guofeng You. 2016. “Nedd4-2 but Not Nedd4-1 Is Critical for Protein Kinase C-Regulated Ubiquitination, Expression, and Transport Activity of Human Organic Anion Transporter 1.” *American Journal of Physiology-Renal Physiology* 310 (9): 821–31. https://doi.org/10.1152/ajprenal.00522.2015.

Xu, Hui Ming, Bing Liao, Qian Jun Zhang, Bei Bei Wang, Hui Li, Xiao Min Zhong, Hui Zhen Sheng, Ying Xin Zhao, Ying Ming Zhao, and Ying Jin. 2004. “Wwp2, An E3 Ubiquitin Ligase That Targets Transcription Factor Oct-4 for Ubiquitination.” *Journal of Biological Chemistry* 279 (22): 23495–503. https://doi.org/10.1074/jbc.M400516200.

Xu, Lei, Jidong Zhu, Xiaofang Hu, Hong Zhu, Hyoung Tae Kim, Joshua LaBaer, Alfred Goldberg, and Junying Yuan. 2007. “C-IAP1 Cooperates with Myc by Acting as a Ubiquitin Ligase for Mad1.” *Molecular Cell* 28 (5): 914–22. https://doi.org/10.1016/j.molcel.2007.10.027.

Xue, Jianfei, Yaohui Chen, Yamei Wu, Zhongyong Wang, Aidong Zhou, Sicong Zhang, Kangyu Lin, et al. 2015. “Tumour Suppressor TRIM33 Targets Nuclear β-Catenin Degradation.” *Nature Communications* 6: 6156. https://doi.org/10.1038/ncomms7156.

Yamauchi, Keiko, Keiji Wada, Kunikazu Tanji, Makoto Tanaka, and Tetsu Kamitani. 2008. “Ubiquitination of E3 Ubiquitin Ligase TRIM5α and Its Potential Role.” *FEBS Journal* 275 (7): 1540–55. https://doi.org/10.1111/j.1742-4658.2008.06313.x.

Yan, Feng-Juan, Xiao-Jing Zhang, Wen-Xin Wang, Yan-Xiao Ji, Pi-Xiao Wang, Yang Yang, Jun Gong, et al. 2017. “The E3 Ligase Tripartite Motif 8 Targets TAK1 to Promote Insulin Resistance and Steatohepatitis.” *Hepatology (Baltimore, Md.)* 65 (5): 1492–1511. https://doi.org/10.1002/hep.28971.

Yan, Jie, Qi Li, Ai Ping Mao, Ming Ming Hu, and Hong Bing Shu. 2014. “TRIM4 Modulates Type i Interferon Induction and Cellular Antiviral Response by Targeting RIG-I for K63-Linked Ubiquitination.” *Journal of Molecular Cell Biology* 6 (2): 154–63. https://doi.org/10.1093/jmcb/mju005.

Yang, Liuzhong, Bing Zhou, Xiaorui Li, Zhihong Lu, Weiwei Li, Xiaoqing Huo, and Zhanhui Miao. 2015. “RNF125 Is a Ubiquitin-Protein Ligase That Promotes P53 Degradation.” *Cellular Physiology and Biochemistry*. https://doi.org/10.1159/000369691.

Yang, Qi-heng. 2004. “Smac / DIABLO Selectively Reduces the Levels of c-IAP1 and c-IAP2 but Not That of XIAP and Livin in HeLa Cells *” 279 (17): 16963–70. https://doi.org/10.1074/jbc.M401253200.

Yang, Wei Lei, Jing Wang, Chia Hsin Chan, Szu Wei Lee, Alejandro D. Campos, Betty Lamothe, Lana Hur, et al. 2009. “The E3 Ligase TRAF6 Regulates Akt Ubiquitination and Activation.” *Science*. https://doi.org/10.1126/science.1175065.

Yang, Yanzhong, Weicheng Liu, Weiying Zou, Hanzhou Wang, and Hongliang Zong. 2007. “Ubiquitin-Dependent Proteolysis of Trihydrophobin 1 ( TH1 ) by the Human Papilloma Virus E6-Associated Protein ( E6-AP )” 180: 167–80. https://doi.org/10.1002/jcb.21164.

Ye, Jung Sook, Nari Kim, Kyoung Jin Lee, Young Ran Nam, Uk Lee, and Chul Hyun Joo. 2014. “Lysine 63-Linked TANK-Binding Kinase 1 Ubiquitination by Mindbomb E3 Ubiquitin Protein Ligase 2 Is Mediated by the Mitochondrial Antiviral Signaling Protein.” *Journal of Virology* 88 (21): 12765–76. https://doi.org/10.1128/JVI.02037-14.

Yin, Jinlong, Tae-Hoon Kim, Nayun Park, Daye Shin, Hae In Choi, Sungchan Cho, Jong Bae Park, and Jong Heon Kim. 2016. “TRIM71 Suppresses Tumorigenesis via Modulation of Lin28B-Let-7-HMGA2 Signaling.” *Oncotarget* 7 (48): 79854–68. https://doi.org/10.18632/oncotarget.13036.

Ying, Zheng, Hongfeng Wang, Huadong Fan, Xiaodong Zhu, Jiawei Zhou, Erkang Fei, and Guanghui Wang. 2009. “Gp78 , an ER Associated E3 , Promotes SOD1 and Ataxin-3 Degradation.” *Human Molecular Genetics* 18 (22): 4268–81. https://doi.org/10.1093/hmg/ddp380.

Yoo, Young Suk, Yong Yea Park, Jae Hoon Kim, Hyeseon Cho, Song Hee Kim, Ho Soo Lee, Tae Hwan Kim, et al. 2015. “The Mitochondrial Ubiquitin Ligase MARCH5 Resolves MAVS Aggregates during Antiviral Signalling.” *Nature Communications* 6 (August). https://doi.org/10.1038/ncomms8910.

Yu, Jian, Jianping Lan, Yuanyuan Zhu, Xiaoxiao Li, Xiaoyu Lai, and Yu Xue. 2008. “The E3 Ubiquitin Ligase HECTD3 Regulates Ubiquitination and Degradation of Tara.” *Biochemical and Biophysical Research Communications* 367: 805–12. https://doi.org/10.1016/j.bbrc.2008.01.022.

Yu, Yanxing, and Gary S. Hayward. 2010. “The Ubiquitin E3 Ligase RAUL Negatively Regulates Type I Interferon through Ubiquitination of the Transcription Factors IRF7 and IRF3.” *Immunity* 33 (6): 863–77. https://doi.org/10.1016/j.immuni.2010.11.027.

Yudina, Zinaida, Amanda Roa, Rory Johnson, Nikolaos Biris, Daniel A. de Souza Aranha Vieira, Vladislav Tsiperson, Natalia Reszka, et al. 2015. “RING Dimerization Links Higher-Order Assembly of TRIM5α to Synthesis of K63-Linked Polyubiquitin.” *Cell Reports* 12 (5): 788–97. https://doi.org/10.1016/j.celrep.2015.06.072.

Zaarour, Rania F, Dafne Chirivino, Laurence Del Maestro, Laurent Daviet, Azeddine Atfi, Daniel Louvard, and Monique Arpin. 2012. “Ezrin Ubiquitylation by the E3 Ubiquitin Ligase , WWP1 , and Consequent Regulation of Hepatocyte Growth Factor Receptor Activity.” *PLoS ONE* 7 (5): 1–10. https://doi.org/10.1371/journal.pone.0037490.

Zaman, M. M.-U., T. Nomura, T. Takagi, T. Okamura, W. Jin, T. Shinagawa, Y. Tanaka, and S. Ishii. 2013. “Ubiquitination-Deubiquitination by the TRIM27-USP7 Complex Regulates Tumor Necrosis Factor Alpha-Induced Apoptosis.” *Molecular and Cellular Biology* 33 (24): 4971–84. https://doi.org/10.1128/mcb.00465-13.

Zattas, Dimitrios, Jason M Berk, Stefan G Kreft, and Mark Hochstrasser. 2016. “A Conserved C-Terminal Element in the Yeast Doa10 and Human MARCH6 Ubiquitin Ligases Required for Selective Substrate Degradation.” *Journal of Biological Chemistry* 291 (23): 12105–18. https://doi.org/10.1074/jbc.M116.726877.

Zelcer, Noam, Cynthia Hong, Rima Boyadjian, and Peter Tontonoz. 2009. “LXR Regulates Cholesterol Uptake through Idol-Dependent Ubiquitination of the LDL Receptor.” *Science* 325 (5936): 100–104. https://doi.org/10.1126/science.1168974.

Zemirli, Naima, Marie Pourcelot, Gorbatchev Ambroise, Emeline Hatchi, and Damien Arnoult. 2014. “Mitochondrial Hyperfusion Promotes NF- j B Activation via the Mitochondrial E3 Ligase MULAN.” *FEBS Journal* 281 (14): 3095–3112. https://doi.org/10.1111/febs.12846.

Zeng, Taoling, Qun Wang, Jieying Fu, Qi Lin, Jing Bi, Weichao Ding, Yikai Qiao, et al. 2014. “Impeded Nedd4-1-Mediated Ras Degradation Underlies Ras-Driven Tumorigenesis.” *Cell Reports* 7 (3): 871–82. https://doi.org/10.1016/j.celrep.2014.03.045.

Zhang, Haoxing, Hailong Liu, Yali Chen, Xu Yang, Panfei Wang, Tongzheng Liu, Min Deng, et al. 2016. “A Cell Cycle-Dependent BRCA1-UHRF1 Cascade Regulates DNA Double-Strand Break Repair Pathway Choice.” *Nature Communications* 7: 10201. https://doi.org/10.1038/ncomms10201.

Zhang, Jiazhen, Thomas Macartney, Mark Peggie, and Philip Cohen. 2017. “Interleukin-1 and TRAF6-Dependent Activation of TAK1 in the Absence of TAB2 and TAB3.” *The Biochemical Journal* 474 (13): 2235–48. https://doi.org/10.1042/BCJ20170288.

Zhang, Jindong, Chuanxia Zhang, Jun Cui, Jiayu Ou, Jing Han, Yunfei Qin, Feng Zhi, and Rong-fu Wang. 2017. “TRIM45 Functions as a Tumor Suppressor in the Brain via Its E3 Ligase Activity by Stabilizing P53 through K63-Linked Ubiquitination.” *Nature Publishing Group*, no. 185: 1–11. https://doi.org/10.1038/cddis.2017.149.

Zhang, Jing, Ming-ming Hu, Yan-yi Wang, and Hong-bing Shu. 2012. “TRIM32 Protein Modulates Type I Interferon Induction and Cellular Antiviral Response by Targeting MITA / STING Protein.” *Journal of Biological Chemistry* 287 (34): 28646–55. https://doi.org/10.1074/jbc.M112.362608.

Zhang, Rui, Jian Zhao, Yuhua Song, Xu Wang, Lili Wang, Jian Xu, Chun Song, and Fang Liu. 2014. “The E3 Ligase RNF34 Is a Novel Negative Regulator of the NOD1 Pathway.” *Cellular Physiology and Biochemistry* 33 (6): 1954–62. https://doi.org/10.1159/000362972.

Zhang, Tianyi, Janet Cronshaw, Nnennaya Kanu, Ambrosius P. Snijders, and Axel Behrens. 2014. “UBR5-Mediated Ubiquitination of ATMIN Is Required for Ionizing Radiation-Induced ATM Signaling and Function.” *Proceedings of the National Academy of Sciences of the United States of America* 111 (33): 12091–96. https://doi.org/10.1073/pnas.1400230111.

Zhang, Ye, Xiao-hong Liao, Hong-yan Xie, and Zhi-min Shao. 2017. “RBR-Type E3 Ubiquitin Ligase RNF144A Targets PARP1 for Ubiquitin-Dependent Degradation and Regulates PARP Inhibitor Sensitivity in Breast Cancer Cells.” *Oncotarget* 8 (55): 94505–18.

Zhang, Yong, Dailing Mao, William T. Roswit, Xiaohua Jin, Anand C. Patel, Dhara A. Patel, Eugene Agapov, et al. 2015. “PARP9-DTX3L Ubiquitin Ligase Targets Host Histone H2BJ and Viral 3C Protease to Enhance Interferon Signaling and Control Viral Infection.” *Nature Immunology* 16 (12): 1215–27. https://doi.org/10.1038/ni.3279.

Zhao, Ming, Mei Qiao, Babatunde O Oyajobi, Gregory R Mundy, and Di Chen. 2003. “E3 Ubiquitin Ligase Smurf1 Mediates Core-Binding Factor Alpha1/Runx2 Degradation and Plays A Specific Role in Osteoblast Differentiation.” *Journal of Biological Chemistry* 278 (30): 27939–44. https://doi.org/10.1074/jbc.M304132200.

Zhao, Xudong, Julian Ik-Tsen Heng, Daniele Guardavaccaro, Richeng Jiang, Michele Pagano, Francois Guillemot, Antonio Iavarone, and Anna Lasorella. 2008. “The HECT-Domain Ubiquitin Ligase Huwe1 Controls Neural Differentiation and Proliferation by Destabilizing the N-Myc Oncoprotein.” *Nature Cell Biology* 10 (6): 643–53. https://doi.org/10.1038/ncb1727.

Zhao, Yongge, Dietrich B Conze, John A Hanover, and Jonathan D Ashwell. 2007. “Tumor Necrosis Factor Receptor 2 Signaling Induces Selective C-IAP1-Dependent ASK1 Ubiquitination and Terminates Mitogen-Activated Protein Kinase Signaling.” *Journal of Biological Chemistry* 282 (11): 7777–82. https://doi.org/10.1074/jbc.M609146200.

Zhou, Ruifeng, Saumil V Patel, and Peter M Snyder. 2007. “Nedd4-2 Catalyzes Ubiquitination and Degradation of Cell Surface ENaC.” *Journal of Biological Chemistry* 282 (28): 20207–12. https://doi.org/10.1074/jbc.M611329200.

Zhou, Ying, Lu Li, Qiongming Liu, Guichun Xing, Xuezhang Kuai, Jing Sun, Xiushan Yin, Jian Wang, Lingqiang Zhang, and Fuchu He. 2008. “E3 Ubiquitin Ligase SIAH1 Mediates Ubiquitination and Degradation of TRB3.” *Cellular Signalling* 20: 942–48. https://doi.org/10.1016/j.cellsig.2008.01.010.

Zhou, Zhongmei, Rong Liu, and Ceshi Chen. 2012. “The WWP1 Ubiquitin E3 Ligase Increases TRAIL Resistance in Breast Cancer.” *International Journal of Cancer* 130 (7): 1504–10. https://doi.org/10.1002/ijc.26122.

Zhu, Fuxiang, Gang Yi, Xu Liu, Fangming Zhu, Anna Zhao, Aiting Wang, Ruihong Zhu, et al. 2018. “Ring Finger Protein 31-Mediated Atypical Ubiquitination Stabilizes Forkhead Box P3 and Thereby Stimulates Regulatory T-Cell Function.” *The Journal of Biological Chemistry* 293 (52): 20099–111. https://doi.org/10.1074/jbc.RA118.005802.

Zurek, Birte, Ida Schoultz, Andreas Neerincx, Luisa M Napolitano, Katharina Birkner, Eveline Bennek, Gernot Sellge, et al. 2012. “TRIM27 Negatively Regulates NOD2 by Ubiquitination and Proteasomal Degradation.” *PLoS ONE* 7 (7): e41255. https://doi.org/10.1371/journal.pone.0041255.
